# Past SARS-CoV-2 infection protection against re-infection: a systematic review and meta-analysis

**DOI:** 10.1016/S0140-6736(22)02465-5

**Published:** 2023-03-11

**Authors:** COVID-19 Forecasting Team, Caroline Stein, Hasan Nassereldine, Reed J D Sorensen, Joanne O Amlag, Catherine Bisignano, Sam Byrne, Emma Castro, Kaleb Coberly, James K Collins, Jeremy Dalos, Farah Daoud, Amanda Deen, Emmanuela Gakidou, John R Giles, Erin N Hulland, Bethany M Huntley, Kasey E Kinzel, Rafael Lozano, Ali H Mokdad, Tom Pham, David M Pigott, Robert C Reiner Jr., Theo Vos, Simon I Hay, Christopher J L Murray, Stephen S Lim

## Abstract

**Background:**

Understanding the level and characteristics of protection from past SARS-CoV-2 infection against subsequent re-infection, symptomatic COVID-19 disease, and severe disease is essential for predicting future potential disease burden, for designing policies that restrict travel or access to venues where there is a high risk of transmission, and for informing choices about when to receive vaccine doses. We aimed to systematically synthesise studies to estimate protection from past infection by variant, and where data allow, by time since infection.

**Methods:**

In this systematic review and meta-analysis, we identified, reviewed, and extracted from the scientific literature retrospective and prospective cohort studies and test-negative case-control studies published from inception up to Sept 31, 2022, that estimated the reduction in risk of COVID-19 among individuals with a past SARS-CoV-2 infection in comparison to those without a previous infection. We meta-analysed the effectiveness of past infection by outcome (infection, symptomatic disease, and severe disease), variant, and time since infection. We ran a Bayesian meta-regression to estimate the pooled estimates of protection. Risk-of-bias assessment was evaluated using the National Institutes of Health quality-assessment tools. The systematic review was PRISMA compliant and was registered with PROSPERO (number CRD42022303850).

**Findings:**

We identified a total of 65 studies from 19 different countries. Our meta-analyses showed that protection from past infection and any symptomatic disease was high for ancestral, alpha, beta, and delta variants, but was substantially lower for the omicron BA.1 variant. Pooled effectiveness against re-infection by the omicron BA.1 variant was 45·3% (95% uncertainty interval [UI] 17·3–76·1) and 44·0% (26·5–65·0) against omicron BA.1 symptomatic disease. Mean pooled effectiveness was greater than 78% against severe disease (hospitalisation and death) for all variants, including omicron BA.1. Protection from re-infection from ancestral, alpha, and delta variants declined over time but remained at 78·6% (49·8–93·6) at 40 weeks. Protection against re-infection by the omicron BA.1 variant declined more rapidly and was estimated at 36·1% (24·4–51·3) at 40 weeks. On the other hand, protection against severe disease remained high for all variants, with 90·2% (69·7–97·5) for ancestral, alpha, and delta variants, and 88·9% (84·7–90·9) for omicron BA.1 at 40 weeks.

**Interpretation:**

Protection from past infection against re-infection from pre-omicron variants was very high and remained high even after 40 weeks. Protection was substantially lower for the omicron BA.1 variant and declined more rapidly over time than protection against previous variants. Protection from severe disease was high for all variants. The immunity conferred by past infection should be weighed alongside protection from vaccination when assessing future disease burden from COVID-19, providing guidance on when individuals should be vaccinated, and designing policies that mandate vaccination for workers or restrict access, on the basis of immune status, to settings where the risk of transmission is high, such as travel and high-occupancy indoor settings.

**Funding:**

Bill & Melinda Gates Foundation, J Stanton, T Gillespie, and J and E Nordstrom.

## Introduction

As of June 1, 2022, the COVID-19 pandemic had caused an estimated 17·2 million total deaths (6·88 million reported deaths), and an estimated 7·63 billion total infections and re-infections.^1^, ^2^, ^3^, ^4^ A large proportion of these infections occurred after Nov 14, 2022; 3·8 billion people or 46% of the global population are estimated to have been infected by the omicron variant and its sublineages.^3^ With strict physical distancing mandates increasingly unwelcome to populations and politicians alike,^5^ the burden of COVID-19 will be largely a function of the coverage of vaccines and their corresponding efficacy, the level of protection afforded by those who have previously been infected by any of the series of SARS-CoV-2 variants, the role of antivirals in averting COVID-19 hospitalisations and deaths,^6^ and the transmissibility and severity of circulating variants. The key dimensions of this protection from previous infection are the extent to which immunity wanes over time and how that protection varies by variant.

Understanding the characteristics of protection from past infection is also necessary in designing science-based policies on the timing of vaccine doses and mandates that require mask wearing, travel restrictions, or access to venues where the risk of transmission is high, such as restaurants, gyms, and places of large indoor gatherings. Virtually all governments have, at some point during the pandemic, limited access to these venues to those who were fully vaccinated or have proof of a recent negative test.^7^, ^8^ Employers and governments have also mandated vaccination for certain classes of workers, particularly those working with vulnerable populations. More variable in implementation is whether those policies allow individuals who are unvaccinated and who have proof of a past infection to qualify. The EU COVID certificate^9^ allowed those with a documented infection within the past 180 days to qualify for the certificate alongside individuals whose last vaccine dose (last dose of the primary series or booster dose) was within 14 days and 270 days. By contrast, USA regulations,^10^ among others,^11^, ^12^, ^13^ required non-citizens to be fully vaccinated (primary series) to travel to the USA. Unvaccinated non-citizens with a past documented infection are not able to enter the country.

**Research in context**

**Evidence before this study**

The future potential burden of COVID-19 is determined by levels and trends in population susceptibility to infection and symptomatic disease. Susceptibility in turn is a function of three main drivers, the coverage of vaccines and their corresponding efficacy, and the level of protection afforded by those who have previously been infected. Individual studies have documented the effectiveness of past infection in preventing re-infection and subsequent symptomatic disease and severe disease (hospitalisation or death), including the extent to which immunity wanes over time. Several systematic reviews of these studies have been done, but none have comprehensively assessed the level of protection by variant and, more importantly, the extent to which immunity from past infection will wane over time.

**Added value of this study**

This study provides a comprehensive review of studies that have estimated the protection from past COVID-19 infection by variant and time since infection. The result shows high levels of protection against re-infection for ancestral, alpha, and delta variants for all major outcomes. Our analysis found significantly reduced protection against re-infection from the omicron BA.1 variant but that levels of protection against severe disease remained high. Although protection from re-infection from all variants wanes over time, our analysis of the available data suggests that the level of protection afforded by previous infection is at least as high, if not higher than that provided by two-dose vaccination using high-quality mRNA vaccines (Moderna and Pfizer-BioNTech), as documented by Nassereldine and colleagues, in our companion study. To our knowledge, this is the first review to comprehensively assess natural immunity protection against COVID-19 re-infection by variant (primary infection and re-infection) and to evaluate waning immunity with time since primary infection.

**Implications of all the available evidence**

Our findings confirm that past infection affords significantly reduced protection against re-infection by the omicron BA.1 variant compared to previous variants, highlighting the high immune escape features of this variant. Our finding that the level of protection from past infection by variant and over time is equivalent to that provided by two-dose mRNA vaccines has important implications for guidance regarding the timing of vaccine doses, including boosters. This finding also has important implications for the design of policies that restrict access to travel or venues or require vaccination for workers. It supports the idea that those with a documented infection should be treated similarly to those who have been fully vaccinated with high-quality vaccines. This was implemented, for example, as part of the EU COVID certificate, but not in countries such as the USA. The scarcity of data on protection afforded by past infection from the omicron BA.1 variant and its sublineages (BA.2, BA.4, and BA.5) highlights the importance of continued assessment, particularly considering that an estimated 46% of the global population was infected by the omicron variant between Nov 15, 2021, and June 1, 2022.

Since January, 2021, several studies^14^, ^15^, ^16^, ^17^ have documented the effectiveness of past COVID-19 infection in reducing the risk of re-infection, including the extent to which immunity wanes over time.^18^ These studies vary substantially in terms of the time period over which protection is assessed, and the variant for which re-infection risk is evaluated. Several in-vitro studies have detected high levels of neutralising antibodies after infection.^19^, ^20^, ^21^ Systematic reviews and meta-analyses have been done on the risks of re-infection;^22^, ^23^, ^24^ however, to date, none have comprehensively assessed how re-infection risk varies by time since infection or stratified results by variant. The objective of this study is to systematically synthesise all available studies to estimate protection from past infection by variant, and where data allow, by time since infection.

## Methods

### Study design

In this systematic review and meta-analysis, we did a living systematic review,^25^ and report here on data published from inception up to Sept 31, 2022, for studies that reported results on protection from past COVID-19 infection. We searched peer-reviewed publications, reports, preprints, medRxiv, and news articles. We routinely searched PubMed, Web of Science, medRxiv, SSRN, and the bibliographies of the included papers using the following keywords: “COVID-19”, “SARS-CoV-2”, “natural immunity”, “previous infection”, “past infection”, “protection”, and “reinfection”. The search was not limited to any language.

The protocol of this study is registered at PROSPERO international database (number CRD42022303850). This study complies with the Guidelines for Accurate and Transparent Health Estimates Reporting^26^ and the PRISMA^27^ recommendations (appendix pp 4–5). All code used in the analyses is available at GitHub.^28^

### Inclusion and exclusion criteria

Any study with results for the protective effect of COVID-19 natural immunity in individuals who were non-vaccinated in comparison with those who were non-vaccinated and COVID-19 naive were included in our analysis. We also included studies that included individuals who were vaccinated but controlled for vaccination status. We included retrospective and prospective cohort studies, and test-negative case-control studies. Any study that included results only for the protective effectiveness of natural immunity in combination with vaccination (ie, hybrid immunity) was excluded from the analysis.

### Outcomes

Re-infection was defined by the following characteristics: a positive SARS-CoV-2 PCR test or a rapid-antigen test (RAT) more than 90 days (or in some studies 120 days) after a previously positive PCR test or RAT; two positive PCR tests or RATs separated by four consecutive negative PCR tests; or a positive PCR test or RAT in an individual with a positive IgG SARS-CoV-2 anti-spike antibody test. Symptomatic re-infection was defined as re-infection with SARS-CoV-2 that leads to the development of symptoms, which may include but are not limited to fever, new or increased cough, new or increased shortness of breath, chills, new or increased muscle pain, new loss of taste or smell, sore throat, diarrhoea, and vomiting. Severe re-infection was re-infection with SARS-CoV-2 that led to hospitalisation or death.

### Study selection and data extraction

We determined on the basis of title and abstract review whether a study or report pertained to infection immunity from COVID-19. If so, the main text and supplementary material were assessed by two independent reviewers on whether it met the inclusion criteria.

We extracted all available data on protection from past infection by primary infection and re-infection variant. Extracted SARS-CoV-2 lineages were ancestral, mixed (two different specified variants; eg, ancestral and alpha, alpha (B.1.1.7), beta (B.1.351), delta (B.1.617.2), and omicron (BA.1), and its sublineages (BA.2 and BA.4/BA.5), the variants were either confirmed through sequencing or inferred from the timing of the infection and included as mixed variants for the studies that did not report specific variants of concern. Where available, we extracted subgroup analyses of protection as a function of time since primary infection. Where these analyses were not available, we extracted the mean time since primary infection. CIs with negative values were changed to 0·01 during the analysis.

The complete information extracted included author, location, study design, primary infection, and re-infection variant (ancestral, mixed, alpha, beta, delta, or omicron), outcomes (re-infection, symptomatic disease, and severe disease), age, protective effect (lower bound and upper bound), average time since infection, time since baseline (weeks), and the method for determining past infection (antibody test or history). Citations and characteristics for all included studies and all data inputs are shown in the appendix (p 28).

The extraction process was completed manually by one reviewer and independently verified by a second reviewer. When there were disagreements, a third reviewer was consulted.

### Risk-of-bias assessment

Each record was evaluated by one reviewer using the National Institutes of Health tools according to study design of the included studies.^29^ Each tool is composed of a series of questions regarding study population, sample, recruitment, measures of exposure or risk and outcome, and potential confounding variables measured and adjusted statistically in the analyses, with possible answers being yes, no, or other. At the end of the evaluation the quality rating could be good, fair, or poor. All studies were treated equally regardless of the quality rating in the primary analysis.

### Data analysis

Risk measures of SARS-CoV-2 infection in individuals with previous infection compared with those who were infection naive (adjusted and unadjusted hazard ratio, adjusted and unadjusted incidence rate ratio, adjusted and unadjusted relative risk, or adjusted and unadjusted odds ratio and CI according to the results available from each study) were extracted from each study. We used adjusted effect sizes where available, otherwise we used unadjusted effect sizes.

Using Bayesian meta-regression we estimated the pooled effect size in logit space using the meta-regression—Bayesian, regularised, trimmed modelling tool (MR-BRT).^30^ The distribution of random intercepts is assumed to be Gaussian in logit space. We used study-level random intercepts and a spline on time since infection to make the estimates, including studies that had subgroup analyses of time since infection and including studies based on the mean time since infection of the study population. We used a uniform prior on the coefficients for the spline basis functions that implement the monotonicity constraint for the spline. The numbers of knots were six internal knots for curves representing about 60 weeks after infection and eight knots for curves representing about 80 weeks after infection. Knots were spaced evenly over the domain between the lowest-observed values and highest-observed values. We estimated 95% uncertainty intervals (UIs)^31^ from fixed effects and between-study heterogeneity using simulation analysis (1000 draws). We did a sensitivity analysis of the meta-analysis by risk-of-bias assessment. We assessed publication bias using Egger's regression test for funnel-plot asymmetry.

Analyses were completed using R version 1.4.1103. The function used was MR-BRT from the mrtool Python package.^27^ Tidyverse, data.table, stringi, ggplot2, forestplot, formattable, crosswalk002, metafor, and mrbrt002 packages were used.

### Role of the funding source

The funders of the study had no role in the study design, data collection, data analysis, data interpretation, or the writing of the report.

## Results

**Figure 1:**
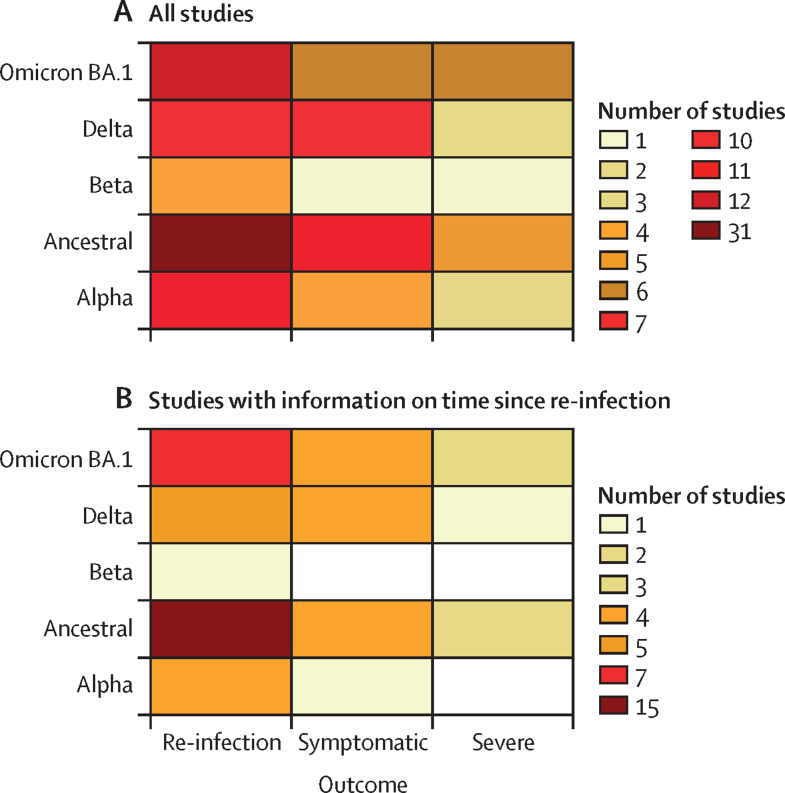
Data availability (number of input studies) by SARS-CoV-2 variant and outcome for the systematic review as a whole and for the analysis of time since infection (A) Number of studies available for inclusion in any component of the systematic review. (B) Number of studies available for inclusion specifically in the analysis of time since infection. Studies were included in this analysis if they included information on time since infection.

We identified 65 studies from 19 different countries (Austria, Belgium, Brazil, Canada, Czechia, Denmark, France, India, Italy, Netherlands, Nicaragua, Norway, Qatar, Scotland, South Africa, Sweden, Switzerland, the UK, and the USA; figure 1A). A total of 30 studies included information on time since infection (figure 1B); 18 of those studies explicitly analysed protection as a function of time since infection. For the remaining 13 studies, we were able to identify the average time since infection for the study population.

The studies used a variety of approaches to determine past infection status. 16 studies relied on antibody testing alone, 38 studies relied on confirmed test (PCR or RAT) history alone, nine studies used a combination of antibody testing and confirmed test history, and two studies did not specify which approach was used.

**Figure 2:**
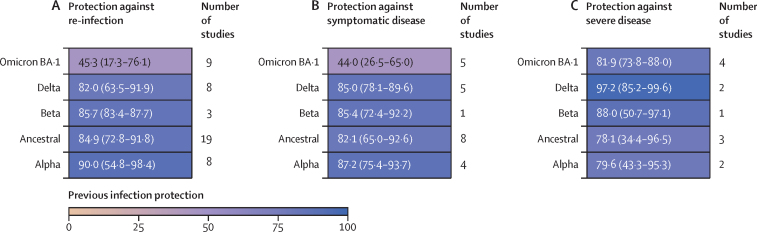
Pooled estimate of protection from past SARS-CoV-2 infection against re-infection, symptomatic disease, and severe disease by variant, and number of included studies in each meta-analysis estimate Data are pooled estimate (95% uncertainty interval). Estimates of protection against re-infection (A), symptomatic disease (B), and severe disease (C).

We found that protection against re-infection was high, with a mean pooled estimate greater than 82% for ancestral, alpha, beta, and delta variants (figure 2A; appendix p 9). By comparison, protection by past infection of earlier variants against re-infection by the omicron BA.1 variant was substantially reduced, with a pooled effectiveness of only 45·3% (95% UI 17·3–76·1; figure 2A; appendix p 10). Protection against symptomatic disease mirrored the results for protection against re-infection. The mean pooled protection from re-infection against symptomatic disease was 82% or greater for ancestral, alpha, beta, and delta variants, and was again substantially reduced for the omicron BA.1 variant (pooled estimate of 44·0%, 26·5–65·0; figure 2B; appendix p 11). By contrast, although based on data from 12 studies, protection against severe disease (hospitalisation or death) was universally high, with mean protection of 78% or greater for ancestral, alpha, beta, delta, and omicron BA.1. The ancestral variant had the lowest pooled estimate, at 78·1% (34·4–96·5) protection against severe disease (figure 2C; appendix p 11). One study^32^ assessed the protection from past omicron BA.1 against sublineages BA.4 and BA.5 with protection of 76·1 (54·9–87·3) against symptomatic disease (table).

**Table:** Protection against omicron sublineages by outcome

	**Country**	**Outcome**	**Primary variant**	**Subsequent variant**	**Protection (95% UI)**	**Weeks after infection**
**Studies without information on time since infection**						
Chemaitelly et al (2022)^33^	Qatar	Infection	Omicron BA.1	Omicron BA.2	94·2 (89·2 to 96·9)	..
Chemaitelly et al (2022)^33^	Qatar	Infection	Omicron BA.2	Omicron BA.1	80·9 (73·1 to 86·4)	..
Altarawneh et al (2022)^32^	Qatar	Infection	Ancestral	Omicron BA.4/BA.5	27·7 (19·3 to 35·2)	..
Altarawneh et al (2022)^32^	Qatar	Infection	Omicron BA.1	Omicron BA.4/BA.5	78·0 (75·0 to 80·7)	..
Andeweg et al (2022)^34^	Netherlands	Infection	Ancestral	Omicron BA.2	47·0 (44·0 to 50·0)	..
Altarawneh et al (2022)^35^	Qatar	Symptomatic	Ancestral	Omicron BA.2	46·1 (39·5 to 51·9)	..
Altarawneh et al (2022)^32^	Qatar	Symptomatic	Ancestral	Omicron BA.4/BA.5	35·5 (12·1 to 52·7)	..
Altarawneh et al (2022)^32^	Qatar	Symptomatic	Omicron BA.1	Omicron BA.4/BA.5	76·2 (66·4 to 83·1)	..
Andeweg et al (2022)^34^	Netherlands	Symptomatic	Ancestral	Omicron BA.2	49·0 (45·0 to 52·0)	..
Powell et al (2022)^36^	UK	Symptomatic	Omicron BA.1	Omicron BA.1	59·3 (46·7 to 69·0)	..
Altarawneh et al (2022)^35^	Qatar	Severe	Ancestral	Omicron BA.2	73·4 (0·2 to 92·9)	..
**Studies with information on time since infection**						
Carazo et al (2022)^37^	Canada	Infection	Ancestral	Omicron BA.2	42·0 (−47·0 to 77·0)	17
Carazo et al (2022)^37^	Canada	Infection	Ancestral	Omicron BA.2	39·0 (0 to 63·0)	37
Carazo et al (2022)^37^	Canada	Infection	Ancestral	Omicron BA.2	42·0 (17·0 to 60·0)	58
Carazo et al (2022)^37^	Canada	Infection	Omicron BA.1	Omicron BA.2	82·0 (49·0 to 94·0)	5
Carazo et al (2022)^37^	Canada	Infection	Omicron BA.1	Omicron BA.2	76·0 (63·0 to 85·0)	9
Carazo et al (2022)^37^	Canada	Infection	Omicron BA.1	Omicron BA.2	70·0 (61·0 to 77·0)	17
Andeweg et al (2022)^34^	Netherlands	Infection	Ancestral	Omicron BA.2	76·0 (68·0 to 82·0)	6
Andeweg et al (2022)^34^	Netherlands	Infection	Ancestral	Omicron BA.2	56·0 (48·0 to 62·0)	10
Andeweg et al (2022)^34^	Netherlands	Infection	Ancestral	Omicron BA.2	50·0 (43·0 to 56·0)	15
Andeweg et al (2022)^34^	Netherlands	Infection	Ancestral	Omicron BA.2	57·0 (49·0 to 64·0)	19
Andeweg et al (2022)^34^	Netherlands	Infection	Ancestral	Omicron BA.2	50·0 (36·0 to 61·0)	23
Andeweg et al (2022)^34^	Netherlands	Infection	Ancestral	Omicron BA.2	53·0 (42·0 to 62·0)	27
Andeweg et al (2022)^34^	Netherlands	Infection	Ancestral	Omicron BA.2	38·0 (34·0 to 43·0)	32
Andeweg et al (2022)^34^	Netherlands	Symptomatic	Ancestral	Omicron BA.2	76·0 (69·0 to 82·0)	6
Andeweg et al (2022)^34^	Netherlands	Symptomatic	Ancestral	Omicron BA.2	57·0 (49·0 to 63·0)	10
Andeweg et al (2022)^34^	Netherlands	Symptomatic	Ancestral	Omicron BA.2	50·0 (43·0 to 56·0)	15
Andeweg et al (2022)^34^	Netherlands	Symptomatic	Ancestral	Omicron BA.2	58·0 (50·0 to 66·0)	19
Andeweg et al (2022)^34^	Netherlands	Symptomatic	Ancestral	Omicron BA.2	55·0 (41·0 to 65·0)	23
Andeweg et al (2022)^34^	Netherlands	Symptomatic	Ancestral	Omicron BA.2	52·0 (40·0 to 62·0)	27·5
Andeweg et al (2022)^34^	Netherlands	Symptomatic	Ancestral	Omicron BA.2	40·0 (35·0 to 44·0)	32

**Figure 3:**
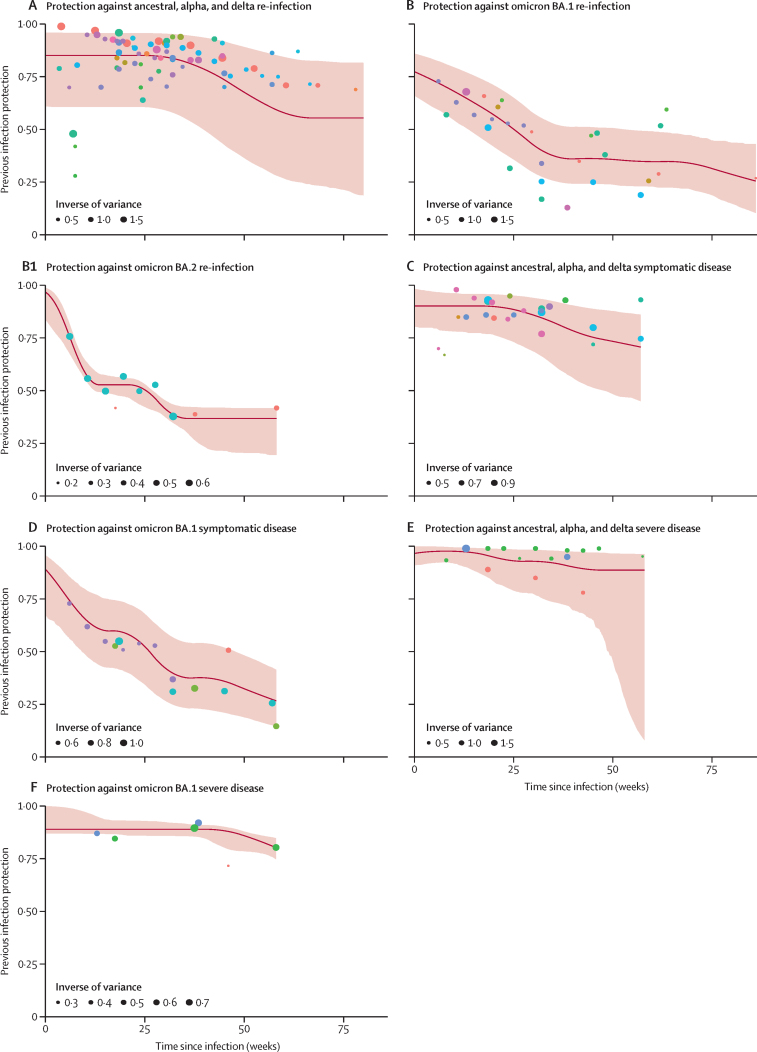
Estimates of protection by time since infection for ancestral, alpha, delta, omicron BA.1, and omicron BA.2 variants Each dot colour represents a different study and its data points according to week after infection. Estimates of protection by time since infection for ancestral, alpha, and delta variants are shown for re-infection (A), symptomatic disease (C), and severe disease (E). Estimates of protection by time since infection for omicron BA.1 are shown for re-infection (B), symptomatic disease (D), and severe disease (F). Estimates of protection by time since infection for omicron BA.2 re-infection (B1).

When evaluating protection against re-infection as a function of time since infection for ancestral, alpha, and delta variants combined, we found that protection was high initially—85·2% (60·8–96·0) at 4 weeks—and declined to 78·6% (49·8–93·6) at 40 weeks. Although based on scarce data, the results showed a protection of 55·5% (18·8–81·7) at 80 weeks (figure 3A; appendix p 50). By contrast to earlier variants, protection from re-infection from the omicron BA.1 variant declined more rapidly, with protection declining to 36·1% (24·4–51·3) at 40 weeks (figure 3B; appendix p 50).

Protection against symptomatic disease by time since infection was similar to that estimated for infection. For the ancestral, alpha, and delta variants combined, protection was 78·4% (56·1–90·5) at 40 weeks (figure 3C; appendix p 50), whereas protection against symptomatic disease was lower for omicron BA.1, with 37·7% (22·8–54·1) at 40 weeks (figure 3D; appendix p 50). However, protection against severe disease remained high for all variants, at 90·2% (69·7–97·5) for ancestral, alpha, and delta; and, 88·9% (84·7–90·9) at 40 weeks for omicron BA.1 (figure 3E and F; appendix p50).

**Figure 4:**
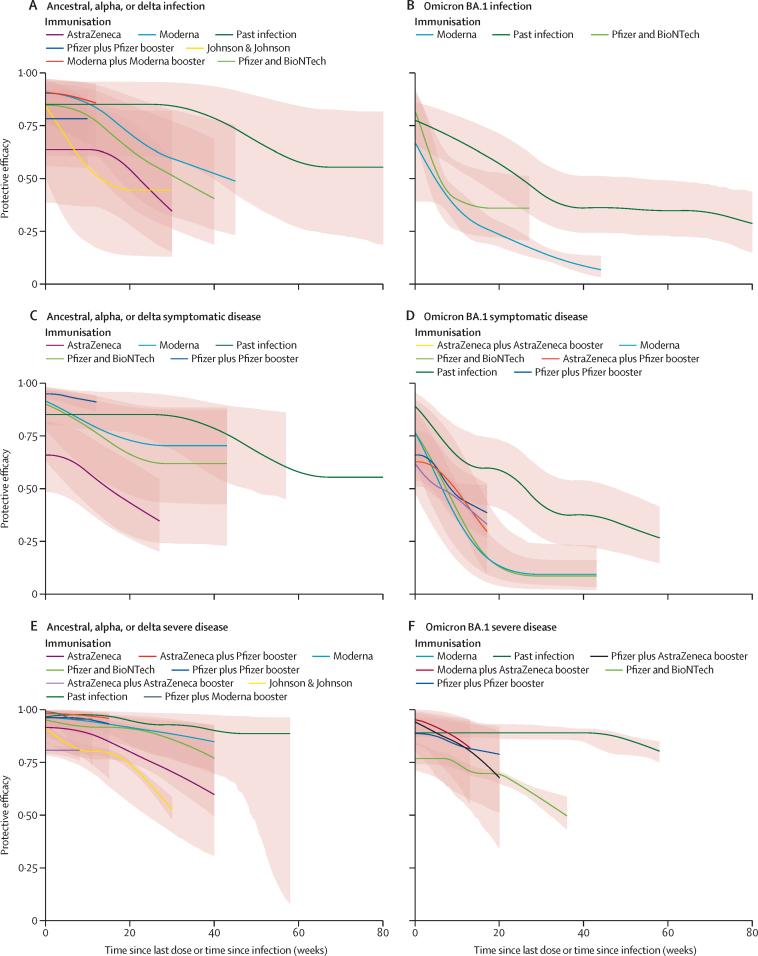
Comparison of protection efficacy from past COVID-19 infection versus protection from vaccination (by vaccine type and dose) against re-infection, symptomatic disease, and severe disease for ancestral, alpha, delta, or omicron BA.1 variants Shading indicates 95% UIs. (A) Comparison between waning of immunity with time of protection conferred by SARS-CoV-2 infection against re-infection with ancestral, alpha, or delta variant versus vaccine protection against primary infection with alpha and delta by type of vaccine and dose. (B) Comparison between waning of immunity with time of protection conferred by SARS-CoV-2 infection against re-infection with omicron variant versus vaccine protection against primary infection with omicron by type of vaccine and dose. (C) Comparison between waning of immunity with time of protection conferred by SARS-CoV-2 infection against symptomatic disease with ancestral, alpha, or delta variant versus vaccine protection against primary infection with alpha and delta by type of vaccine and dose. (D) Comparison between waning of immunity with time of the protection conferred by SARS-CoV-2 infection against symptomatic disease with omicron variant versus vaccine protection against primary infection with omicron by type of vaccine and dose. (E) Comparison between waning of immunity with time of the protection conferred by SARS-CoV-2 infection against severe disease with ancestral, alpha, or delta variant versus vaccine protection against primary infection with alpha and delta by type of vaccine and dose. (F) Comparison between waning of immunity with time of protection conferred by SARS-CoV-2 infection against severe disease with omicron variant versus vaccine protection against primary infection with omicron by type of vaccine and dose. UIs=uncertainty intervals.

Only a small number of studies evaluated protection against omicron sublineages specifically (BA.2 and BA.4 and BA.5). Data by variant and outcome were in general not sufficient to conduct meta-analyses (table). Protection against omicron BA.2 and BA.4 and BA.5 was lower when the past infection was a pre-omicron variant than when the past infection was omicron (table). For example, one study^34^ showed protection against omicron BA.2 re-infection of 47·0% (44·0–50·0) and another one^32^ showed protection against omicron BA.4 and BA.5 of 27·7% (19·3–35·2). Protection was notably higher when the previous infection was omicron BA.1 although remained reduced for BA.4 and BA.5. Other studies^32^, ^33^ showed protection against omicron BA.2 was 94·2% (89·2–96·9) and protection against omicron BA.45 was 78·0% (75·0–80·7). In another study assessing protection against symptomatic disease, infection levels were higher when the previous infection was omicron than when it was pre-omicron.^32^ Protection against omicron BA.4 and BA.5 with omicron BA.1 as the past infection was 76·2% (66·4–83·1) in comparison with 35·5% (12·1–52·7) if the past infection was pre-omicron^32^ (table). Two studies^34^, ^37^ assessed protection from past omicron sublineage BA.2 considering time since infection, showing protection of 85·4 (74·0–91·1) at 4 weeks and 37·0 (23·5–42·2) at 40 weeks against re-infection (figure 3B; appendix p 50). Past COVID-19 infection against re-infection, symptomatic disease, and severe disease for ancestral, alpha, delta, or omicron BA.1 variants, appears to be at least as protective as two-dose vaccination with the mRNA vaccines for all vaccines and outcomes (by vaccine type and dose; figure 4).

14 case-control studies and 51 cohort studies were assessed for risk of bias; 23 studies had a good-quality rating, 32 had a fair-quality rating, and eight had a poor-quality rating (appendix pp 78, 80). Common potential causes of bias among these studies were the absence of a reliable and consistent way of measuring exposure, the absence of sample-size justification in the studies that were not on the national level, and the absence of adjusting for confounding variables during the analysis. One report^38^ was not assessed because of the scarcity of data for assessment.

The sensitivity analysis showed no significant differences in the results by the level of bias (p>0·05; for exact p values see appendix p 13) or the level of adjustment for confounders (p>0·05; for exact p values see appendix p 18). The sensitivity analysis for the level of bias between studies evaluated as being fair and good or good was for omicron BA.1 protection against re-infection (p=0·86), as well as for omicron BA.1 protection against symptomatic disease (p=0·60). The sensitivity analysis for the level of adjustment for confounders (no adjustment or matching and adjusted or matched for age, sex, and other variables) for omicron BA.1 protection against re-infection was not significant (p=0·64). There was no evidence of publication bias for ten of 13 meta-analyses (p>0·05; for exact p values see appendix p 25). For the remaining three meta-analyses, there was evidence of publication bias for protection against re-infection from delta (p=0·011), ancestral variants (p=0·026), and for protection against omicron BA.1 symptomatic disease (p=0·044; appendix p 25).

## Discussion

Our systematic review and meta-analysis provides a comprehensive assessment of the scientific literature on the protection against subsequent SARS-CoV-2 infection, symptomatic disease, and severe disease (hospitalisation or death) afforded by previous infection by variant and by time since the initial infection. Our results show that high levels of protection—on average greater than 85%—are present for ancestral, alpha, delta, and beta variants across all three outcomes (infection, any symptomatic disease, and severe disease). The analysis shows the substantially reduced level of protection against re-infection or any symptomatic disease to less than 55% for the omicron variant, but that protection against severe disease from the omicron variant appears to be maintained at a high level. Only a small number of studies were identified that evaluated protection from past infection against omicron sublineages such as BA.2 and BA.4 and BA.5. In general, the findings for omicron sublineages showed significantly reduced protection when the past infection was pre-omicron. When the past infection was omicron, protection was maintained at a higher level, although less so for BA.4 and BA.5, confirming the greater immune escape associated with this sublineage.^39^

Furthermore, although protection from past infection wanes over time, the level of protection against re-infection, symptomatic disease, and severe disease appears to be at least as durable, if not more so, than that provided by two-dose vaccination with the mRNA vaccines for ancestral, alpha, delta, and omicron BA.1 variants (Nassereldine H et al, unpublished), which is also seen from studies directly comparing natural immunity to vaccine-induced protection.^40^ Protection against severe disease, although based on scarce data, appears to be durable up to more than 1 year for ancestral, alpha, delta, and omicron BA.1 variants. Protection from past infection in comparison with that conferred by vaccination, however, must be weighed against the risks of severe morbidity and mortality associated with the initial infection. This balance of risk varies by the type of variant, with omicron for instance having less severe outcomes than delta,^41^, ^42^ and other risk factors associated with the individual, such as age and other comorbidities.^43^

Our findings are corroborated by other reviews^44^ and studies including in-vitro findings, mechanistic studies of infection, and modelling studies.^45^ Immunity conferred by infection includes both humoral and cellular responses,^46^, ^47^ and there is evidence of diverse T-cell immunity and memory B-cell response to COVID-19 spike-protein antigens, in addition to other protein targets, that could lead to a more sustained immunity with increased protection against the various COVID-19 variants.^48^, ^49^ This mechanism operates alongside the valuable role of mucosal immunity as a barrier protection.^50^, ^51^ The weaker cross-variant immunity with the omicron BA.1 variant and its sublineages further supports the effect spike-protein mutations have on evading immunity in omicron, in comparison with other variants.^52^

Our findings have several important policy implications. First to monitor the risk of future COVID-19 burden, tracking of past infection rates and the variant-specific temporal pattern of infections is essential. Maintaining surveillance systems that track infections and variant emergence (eg, the Real-time Assessment of Community Transmission^53^ study has been an effective tool for monitoring the spread and emergence of variants in England) and spread will continue to be an important aspect of managing current and future COVID-19 transmission. Second, restrictions of movement and access to venues based on immune status and vaccine mandates for workers should take into account immunity conferred by vaccination and that provided by natural infection. Countries have taken different approaches to this; for example, immunity from past infection was considered as part of eligibility for the EU COVID certificate but not in countries such as the USA or Australia.^9^, ^10^, ^13^ Third, the protection afforded by past infection should be considered in guidelines for when people should receive vaccine doses, including boosters. Fourth, as new variants emerge, as highlighted by the omicron variant, timely and well conducted epidemiological studies are needed to understand not only protection afforded by vaccination but also past infection, although it is important to note that the ability to assess protection conferred by infection, by comparing individuals unvaccinated and previously infected to those who are unvaccinated and COVID-19 naive, is increasingly challenging given the small number of people who are unvaccinated and COVID-19 naive remaining in many populations. To date, the number of studies on vaccine efficacy (Nassereldine et al, unpublished) far exceeds the number of studies on the protection from natural infection. These studies should further examine the protection conferred by combinations of vaccination and natural infection.

The primary limitations of our study relate to the limitations of the studies and data included in our systematic review and meta-analysis. First, the number of studies available is generally low, particularly for those that have examined protection as a function of time since infection for severe disease, that report data on the omicron BA.1 variant and its sublineages in particular, and that come from Africa that met our inclusion criteria. Moreover, few data are available beyond a period of 40 weeks after the initial infection. Second, there was evidence of publication bias for three of 13 variant outcomes assessed in our study. Third, in estimating protection, we are relying on observational studies, which are prone to residual confounding. Fourth, studies used a variety of approaches for ascertaining past infection status, comprising antibody prevalence, documented history of infection, or a combination of the two. Incomplete or in some cases over-ascertainment of past infections might bias the estimate of protection. Fifth, underlying studies also vary in the extent to which they measure hospitalisation because of COVID-19 versus hospitalisation with an incidental COVID-19 infection. This bias might affect our estimates of protection against severe disease, particularly during the initial omicron wave when transmission was very high. Finally, in our analyses of protection by time since infection, compositional bias exists in terms of the different time periods that the underlying studies have assessed. We have attempted to control for this bias with the use of study random effects.

Our findings show that immunity from COVID-19 infection confers substantial protection against infection from pre-omicron variants. By comparison, protection against re-infection from the omicron BA.1 variant was substantially reduced and wanes rapidly over time. Protection against severe disease, although based on scarce data, was maintained at a relatively high level up to 1 year after the initial infection for all variants. Our analysis suggests that the level of protection from past infection by variant and over time is at least equivalent if not greater than that provided by two-dose mRNA vaccines.

## Data sharing

To download the data used in these analyses, please visit the Global Health Data Exchange website at https://ghdx.healthdata.org/record/ihme-data/past-sars-cov-2-infection-protection-against-reinfection-systematic-review-meta-analysis-estimates.

## Declaration of interests

DMP reports support from the Bill & Melinda Gates foundation as grant payments made to the Institute for Health Metrics and Evaluation. All other authors declare no competing interests.
