# Supplementary material for: Past SARS-CoV-2 infection protection against re-infection: a systematic review and meta-analysis
Source: Lancet. 2023 Mar 11;401(10379):833–42. doi: 10.1016/S0140-6736(22)02465-5 (PMC9998097; doi:10.1016/S0140-6736(22)02465-5)
Supplement: Supplementary appendix [file mmc1.pdf]

THE LANCET

Supplementary appendix

This appendix formed part of the original submission and has been peer reviewed. We post it as supplied by the authors.

Supplement to: COVID-19 Forecasting Team. Past SARS-CoV-2 infection protection against re-infection: a systematic review and meta-analysis. *Lancet* 2023; published online Feb 16. https://doi.org/10.1016/S0140-6736(22)02465-5.

Supplementary appendix “Past COVID-19 infection protection against reinfection: systematic review and meta-analysis"

This appendix provides further supplemental figures and more detailed results.

The appendix is organised into broad sections following the structure of the main paper.

Table of Contents

Table of Contents 2
Section 1 List of acronyms and abbreviations 3
Section 2 Guidelines for Accurate and Transparent Health Estimates Reporting (GATHER) compliance 4
Section 3 Preferred Reporting Items for Systematic Reviews and Meta-Analyses (PRISMA) compliance 5
Section 4 Supplementary figures and tables 8
Figure S1 Forest plots of meta-analysis results by variant and outcome 9
Figure S2 Sensitivity analysis according to Risk of Bias assessment 13
Figure S3 Sensitivity analysis according to adjusted/unadjusted results 18
Figure S4 Publication bias assessment 25
Table S1 Data inputs and characteristics of included studies 28
Table S2 Protection against reinfection, symptomatic, and severe disease by time since infection 50
Table S3 Risk of Bias Assessment for Case-Control Studies 78
Table S4 Risk of Bias Assessment for Cohort Studies 80
Section 5 Reference list 84

Section 1 List of acronyms and abbreviations

Abbreviation/acronym	Full phrase
SARS-CoV-2	Severe acute respiratory syndrome coronavirus 2
PCR	Polymerase chain reaction
RAT	Rapid antigen test
GATHER	Guidelines for Accurate and Transparent Health Estimates Reporting
PRISMA	Preferred Reporting Items for Systematic Reviews and Meta-Analyses
UI	Uncertainty Interval
MR-BRT	Meta-regression—Bayesian, regularised, trimmed
GHDx	Global Health Data Exchange

Section 2 Guidelines for Accurate and Transparent Health Estimates Reporting (GATHER) compliance

This study complies with GATHER recommendations,[1] See table S2 below for the GATHER checklist. The GATHER recommendations can be found on the GATHER website.

GATHER checklist

Item #	Checklist item	Reported location
Objectives and funding		
1	Define the indicator(s), populations (including age, sex, and geographic entities), and time period(s) for which estimates were made.	*Methods (Study selection and data extraction); appendix Table S1*
2	List the funding sources for the work.	*Summary*
Data Inputs		
For all data inputs from multiple sources that are synthesized as part of the study:		
3	Describe how the data were identified and how the data were accessed.	*Methods (Overview)*
4	Specify the inclusion and exclusion criteria. Identify all ad-hoc exclusions.	*Methods (Inclusion and exclusion criteria)*
5	Provide information on all included data sources and their main characteristics. For each data source used, report reference information or contact name/institution, population represented, data collection method, year(s) of data collection, sex and age range, diagnostic criteria or measurement method, and sample size, as relevant.	*Appendix Table S1*
6	Identify and describe any categories of input data that have potentially important biases (e.g., based on characteristics listed in item 5).	*Methods (Risk of bias assessment); (appendix, table S3 and S4 page 78 and 80*
For data inputs that contribute to the analysis but were not synthesized as part of the study:		
7	Describe and give sources for any other data inputs.	*NA*
For all data inputs:		
8	Provide all data inputs in a file format from which data can be efficiently extracted (e.g., a spreadsheet rather than a PDF), including all relevant meta-data listed in item 5. For any data inputs that cannot be shared because of ethical or legal reasons, such as third-party ownership, provide a contact name or the name of the institution that retains the right to the data.	*Appendix, table S1 page 28*
Data analysis		
9	Provide a conceptual overview of the data analysis method. A diagram may be helpful.	*Methods (Data analysis)*
10	Provide a detailed description of all steps of the analysis, including mathematical formulae. This description should cover, as relevant, data cleaning, data pre-processing, data adjustments and weighting of data sources, and mathematical or statistical model(s).	*Methods (Data analysis)*
11	Describe how candidate models were evaluated and how the final model(s) were selected.	*NA*
12	Provide the results of an evaluation of model performance, if done, as well as the results of any relevant sensitivity analysis.	*Appendix, figure S2 and S3, page 13 and 18*
13	Describe methods for calculating uncertainty of the estimates. State which sources of uncertainty were, and were not, accounted for in the uncertainty analysis.	*Methods (Data analysis)*
14	State how analytic or statistical source code used to generate estimates can be accessed.	*GitHub URL(s) will be made available*
Results and Discussion		
15	Provide published estimates in a file format from which data can be efficiently extracted.	*Appendix, table S1 page 28*
16	Report a quantitative measure of the uncertainty of the estimates (e.g. uncertainty intervals).	*Results*
17	Interpret results in light of existing evidence. If updating a previous set of estimates, describe the reasons for changes in estimates.	*Discussion*
18	Discuss limitations of the estimates. Include a discussion of any modelling assumptions or data limitations that affect interpretation of the estimates.	*Discussion*

Section 3 Preferred Reporting Items for Systematic Reviews and Meta-Analyses (PRISMA) compliance

This study complies with PRISMA 2020 recommendations.[2] See table S3 below for the PRISMA 2020 checklist. The PRISMA recommendations can be found on the PRISMA website.

PRISMA 2020 checklist

Section and topic	Item #	Checklist item	Location where item is reported
TITLE			
Title	1	Identify the report as a systematic review.	Paper title
ABSTRACT			
Abstract	2	See the PRISMA 2020 for Abstracts checklist.	N/A
INTRODUCTION			
Rationale	3	Describe the rationale for the review in the context of existing knowledge.	Main text introduction, paragraph 3
Objectives	4	Provide an explicit statement of the objective(s) or question(s) the review addresses.	Final sentence of main text introduction
METHODS			
Eligibility criteria	5	Specify the inclusion and exclusion criteria for the review and how studies were grouped for the syntheses.	Main text methods "inclusion and exclusion criteria" section
Information sources	6	Specify all databases, registers, websites, organisations, reference lists and other sources searched or consulted to identify studies. Specify the date when each source was last searched or consulted.	Main text methods "overview," paragraph 1
Search strategy	7	Present the full search strategies for all databases, registers and websites, including any filters and limits used.	Main text methods "overview," paragraph 1
Selection process	8	Specify the methods used to decide whether a study met the inclusion criteria of the review, including how many reviewers screened each record and each report retrieved, whether they worked independently, and if applicable, details of automation tools used in the process.	Main text methods "study selection and data extraction" section, paragraph 1
Data collection process	9	Specify the methods used to collect data from reports, including how many reviewers collected data from each report, whether they worked independently, any processes for obtaining or confirming data from study investigators, and if applicable, details of automation tools used in the process.	Main text methods "study selection and data extraction" section
Data items	10a	List and define all outcomes for which data were sought. Specify whether all results that were compatible with each outcome domain in each study were sought (e.g. for all measures, time points, analyses), and if not, the methods used to decide which results to collect.	Main text methods "outcomes assessed" section
	10b	List and define all other variables for which data were sought (e.g. participant and intervention characteristics, funding sources). Describe any assumptions made about any missing or unclear information.	Main text methods "study selection and data extraction" section, paragraph 2
Study risk of bias assessment	11	Specify the methods used to assess risk of bias in the included studies, including details of the tool(s) used, how many reviewers assessed each study and whether they worked independently, and if applicable, details of automation tools used in the process.	Main text methods "risk of bias assessment" section
Effect measures	12	Specify for each outcome the effect measure(s) (e.g. risk ratio, mean difference) used in the synthesis or presentation of results.	Main text methods "data analysis" section

Section and topic	Item #	Checklist item	Location where item is reported
Synthesis methods	13a	Describe the processes used to decide which studies were eligible for each synthesis (e.g. tabulating the study intervention characteristics and comparing against the planned groups for each synthesis (item #5)).	N/A
	13b	Describe any methods required to prepare the data for presentation or synthesis, such as handling of missing summary statistics, or data conversions.	N/A
	13c	Describe any methods used to tabulate or visually display results of individual studies and syntheses.	Main text methods “data analysis” section
	13d	Describe any methods used to synthesize results and provide a rationale for the choice(s). If meta-analysis was performed, describe the model(s), method(s) to identify the presence and extent of statistical heterogeneity, and software package(s) used.	Main text methods “data analysis” section
	13e	Describe any methods used to explore possible causes of heterogeneity among study results (e.g. subgroup analysis, meta-regression).	Main text methods “data analysis” section
	13f	Describe any sensitivity analyses conducted to assess robustness of the synthesized results.	Main text methods “data analysis” section
Reporting bias assessment	14	Describe any methods used to assess risk of bias due to missing results in a synthesis (arising from reporting biases).	Main text methods “Risk of bias assessment” section
Certainty assessment	15	Describe any methods used to assess certainty (or confidence) in the body of evidence for an outcome.	Main text methods “data analysis” section
RESULTS			
Study selection	16a	Describe the results of the search and selection process, from the number of records identified in the search to the number of studies included in the review, ideally using a flow diagram.	Main text results, paragraphs 1 and 2
	16b	Cite studies that might appear to meet the inclusion criteria, but which were excluded, and explain why they were excluded.	N/A
Study characteristics	17	Cite each included study and present its characteristics.	Appendix Table S1
Risk of bias in studies	18	Present assessments of risk of bias for each included study.	Appendix tables S3 and S4
Results of individual studies	19	For all outcomes, present, for each study: (a) summary statistics for each group (where appropriate) and (b) an effect estimate and its precision (e.g. confidence/credible interval), ideally using structured tables or plots.	Appendix table S1
Results of syntheses	20a	For each synthesis, briefly summarise the characteristics and risk of bias among contributing studies.	Main text results, paragraph 8
	20b	Present results of all statistical syntheses conducted. If meta-analysis was done, present for each the summary estimate and its precision (e.g. confidence/credible interval) and measures of statistical heterogeneity. If comparing groups, describe the direction of the effect.	Main text results, paragraphs 3–7
	20c	Present results of all investigations of possible causes of heterogeneity among study results.	NA
	20d	Present results of all sensitivity analyses conducted to assess the robustness of the synthesized results.	Main text results, paragraphs 9
Reporting biases	21	Present assessments of risk of bias due to missing results (arising from reporting biases) for each synthesis assessed.	NA
Certainty of evidence	22	Present assessments of certainty (or confidence) in the body of evidence for each outcome assessed.	95% uncertainty intervals (UIs) are presented for all mean estimates in the main text results section and in all figures, as relevant
DISCUSSION			
Discussion	23a	Provide a general interpretation of the results in the context of other evidence.	Main text discussion,

Section and topic	Item #	Checklist item	Location where item is reported
			particularly paragraph 3
	23b	Discuss any limitations of the evidence included in the review.	Main text discussion, paragraph 5
	23c	Discuss any limitations of the review processes used.	Main text discussion, paragraph 5
	23d	Discuss implications of the results for practice, policy, and future research.	Main text discussion, paragraph 4; conclusion
OTHER INFORMATION			
Registration and protocol	24a	Provide registration information for the review, including register name and registration number, or state that the review was not registered.	Main text methods "overview," second paragraph
	24b	Indicate where the review protocol can be accessed, or state that a protocol was not prepared.	Main text methods "overview," second paragraph
	24c	Describe and explain any amendments to information provided at registration or in the protocol.	N/A
Support	25	Describe sources of financial or non-financial support for the review, and the role of the funders or sponsors in the review.	Main text methods "role of the funding source" section, acknowledgments section
Competing interests	26	Declare any competing interests of review authors.	Main text declaration of interests section
Availability of data, code and other materials	27	Report which of the following are publicly available and where they can be found: template data collection forms; data extracted from included studies; data used for all analyses; analytic code; any other materials used in the review.	Main text data sharing section, methods "overview," second paragraph; Appendix table S1

Section 4 Supplementary figures and tables

Figure S1 Forest plots of meta-analysis results by variant and outcome

Protection against reinfection

Ancestral

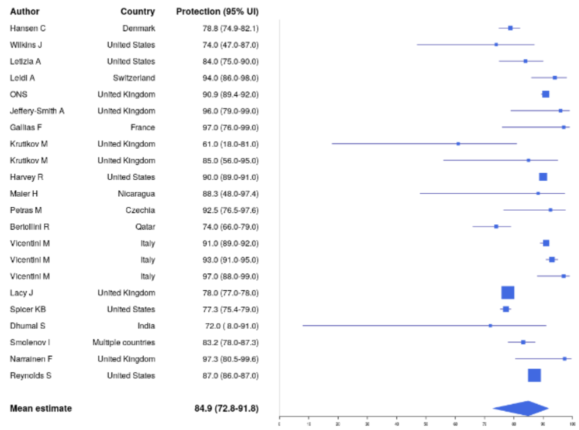

Multiple countries: Belgium, Brazil, Colombia, The Philippines, and South Africa.
Krutikov M (two different age groups: 18-64 and 65-90)
Massimo V (three different primary infection variants: ancestral, Alpha, and Delta)

Alpha

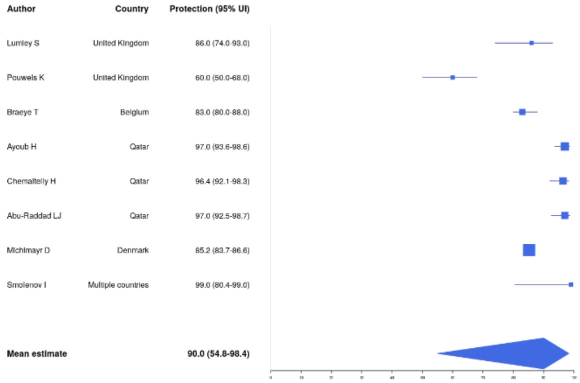

Multiple countries: Belgium, Brazil, Colombia, The Philippines, and South Africa

Beta

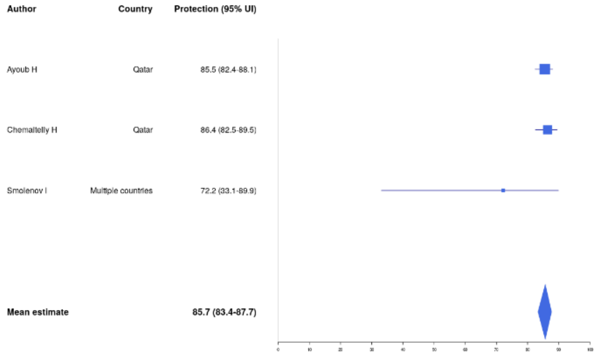

Multiple countries: Belgium, Brazil, Colombia, The Philippines, and South Africa

Delta

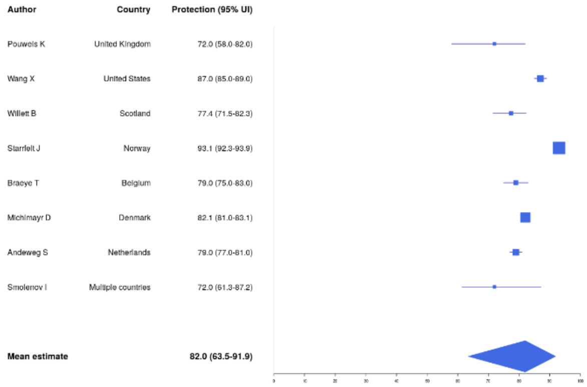

Multiple countries: Belgium, Brazil, Colombia, The Philippines, and South Africa

Omicron BA.1

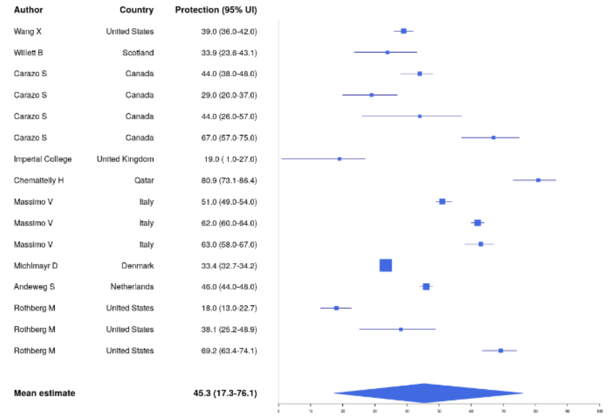

Carazo S (four different primary infection variants: mixed variant, ancestral, Alpha, and Delta)
Massimo V (three different primary infection variants: ancestral, Alpha, and Delta)
Rothberg M (three different primary infection variants: ancestral, Alpha, and Delta)

Protection against symptomatic disease

Ancestral

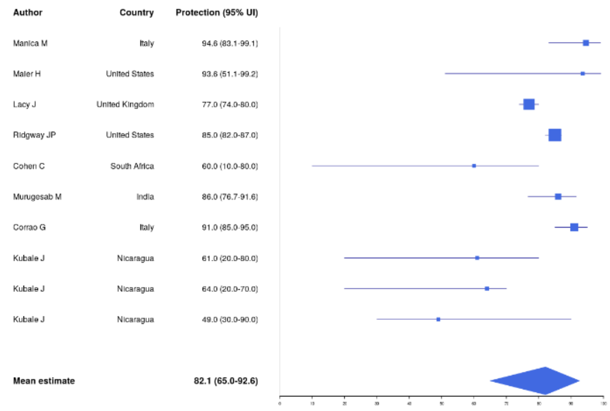

Kubale J (three different age groups: 0-4, 5-9, 10-14)

Alpha

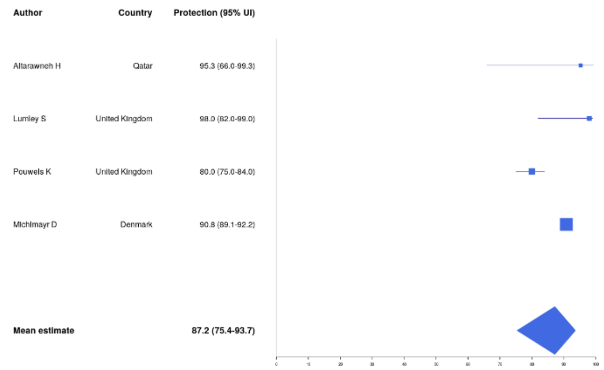

Beta
Author: Altarawneh H
Country: Qatar
Protection (95% UI): 85.4 (72.4–92.2)

Delta

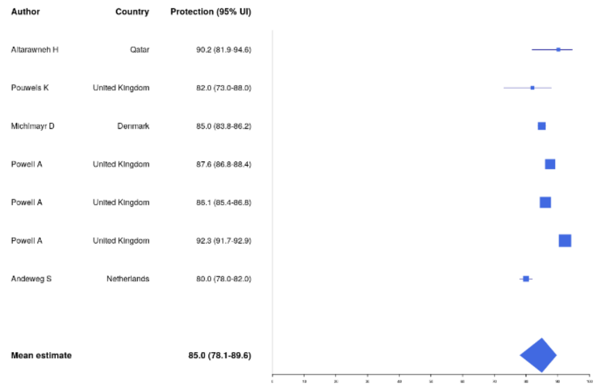

Powell A (three different primary infection variants: ancestral, Alpha, and Delta)

Omicron BA.1

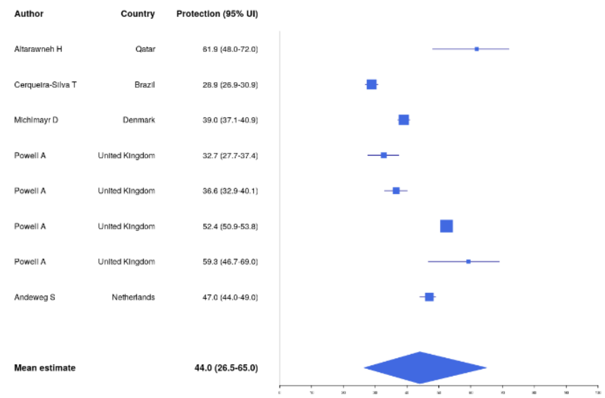

Powell A (four different primary infection variants: ancestral, Alpha, Delta, and Omicron BA.1)

Protection against severe disease

Ancestral

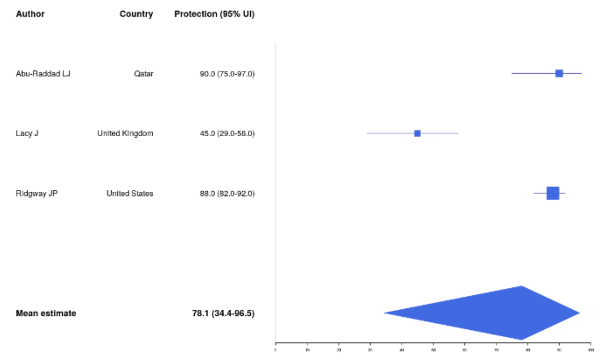

Alpha

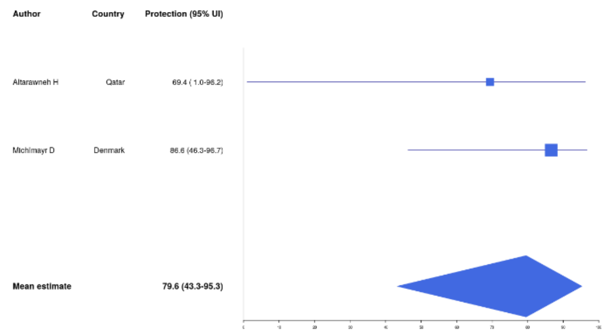

Beta
Author: Altarawneh H
Country: Qatar
Protection (95% UI): 88.0 (50.7–97.1)

Delta

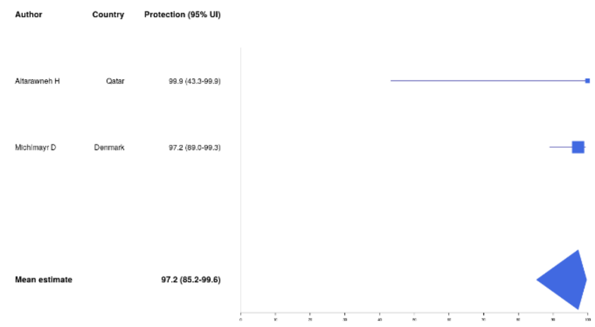

Omicron BA.1

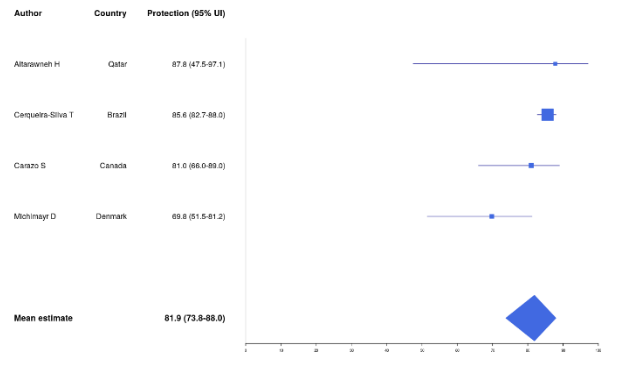

Author
Country
Protection (95% UI)
Altarawneh H
Qatar
87.8 (47.5-97.1)
Cerqueira-Silva T
Brazil
85.6 (82.7-88.0)
Carazo S
Canada
81.0 (66.0-89.0)
Michlmayr D
Denmark
69.8 (51.5-81.2)
Mean estimate
81.9 (73.8-88.0)

Figure S2 Sensitivity analysis according to Risk of Bias assessment

Protection against reinfection

Ancestral

z = -0.06855, p = 0.9453

Fair and Good

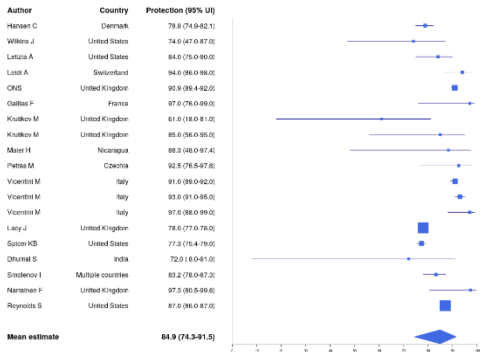

Multiple countries: Belgium, Brazil, Colombia, The Philippines, and South Africa

Good

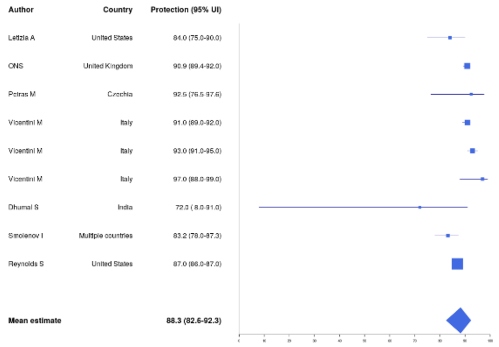

Multiple countries: Belgium, Brazil, Colombia, The Philippines, and South Africa

Alpha

z = -0.08907, p = 0.9290

Fair and Good

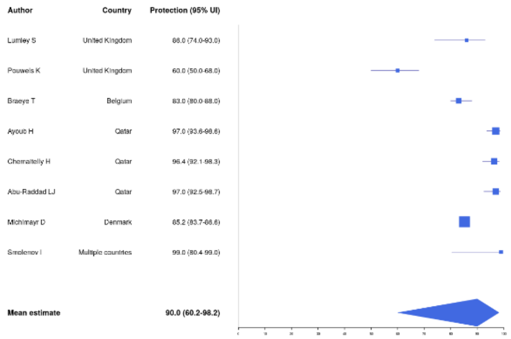

Multiple countries: Belgium, Brazil, Colombia, The Philippines, and South Africa

Good

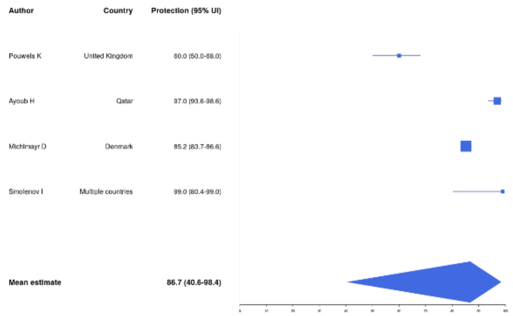

Multiple countries: Belgium, Brazil, Colombia, The Philippines, and South Africa

Beta

z = -0.0226, p = 0.981966

Fair and Good

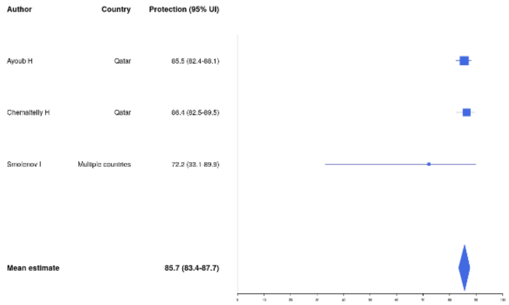

Multiple countries: Belgium, Brazil, Colombia, The Philippines, and South Africa

Good

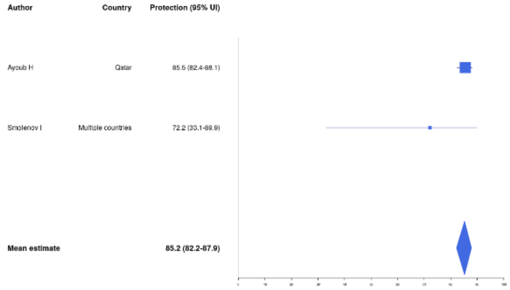

Multiple countries: Belgium, Brazil, Colombia, The Philippines, and South Africa

Delta

z = 0.000428, p = 0.999659

Fair and Good

Good

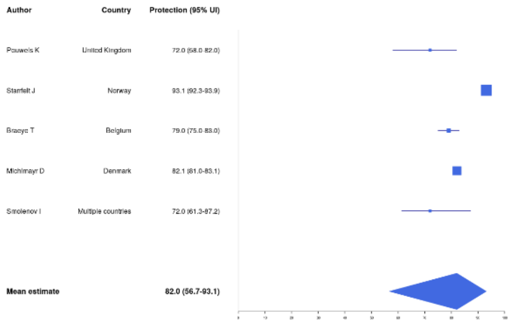

Multiple countries: Belgium, Brazil, Colombia, The Philippines, and South Africa

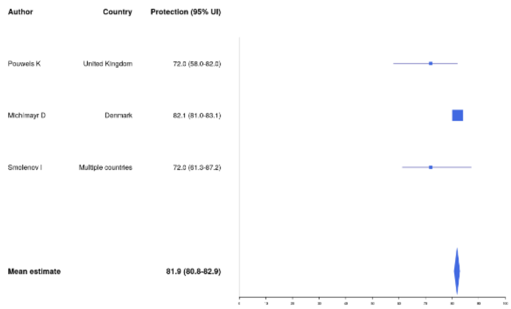

Multiple countries: Belgium, Brazil, Colombia, The Philippines, and South Africa

Omicron BA.1

z = 0.165204, p = 0.868783

Fair and Good

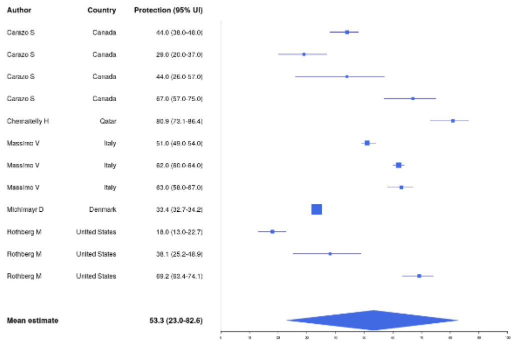

Good

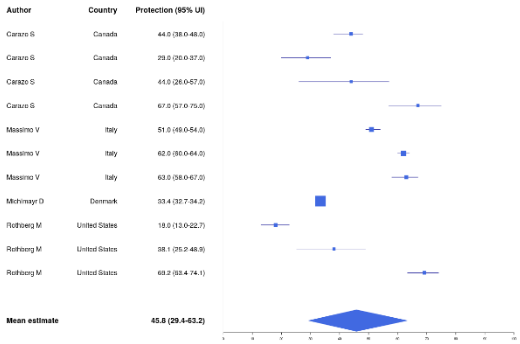

Protection against symptomatic disease

Ancestral

z = -0.39889, p = 0.689973

Fair or Good

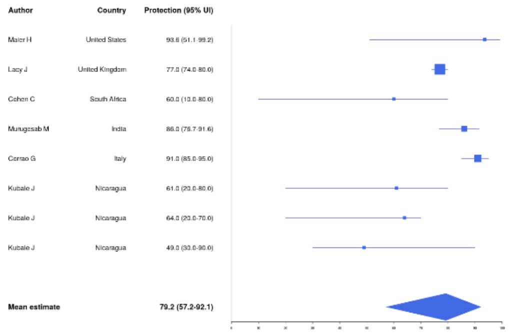

Good

Author: Cohen C

Country: South Africa

Protection (95% UI): 60.0 (10.0-80.0)

Alpha

z = -0.20574, p = 0.836995

Fair and Good

Good

<table>
<tr><td>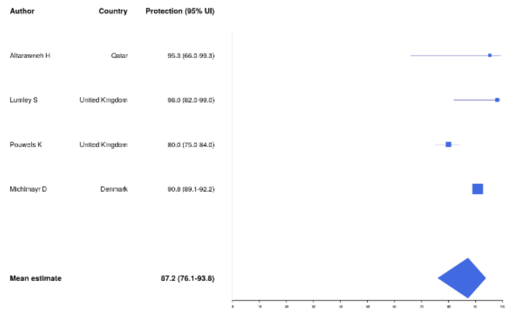
</td><td>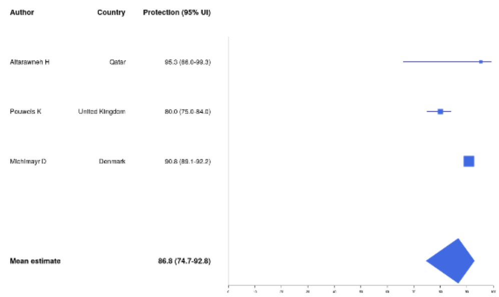
</td></tr>
<tr><td colspan="2">Beta</td></tr>
<tr><td></td><td>Good
Author: Altarawneh H
Country: Qatar
Protection (95% UI): 85.4 (72.4–92.2)</td></tr>
<tr><td colspan="2">Delta
z = 0.089078, p = 0.92902</td></tr>
<tr><td>Fair and Good
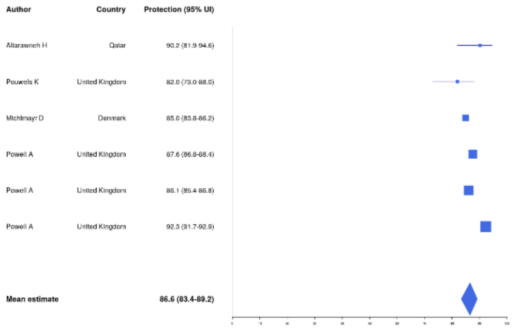
</td><td>Good
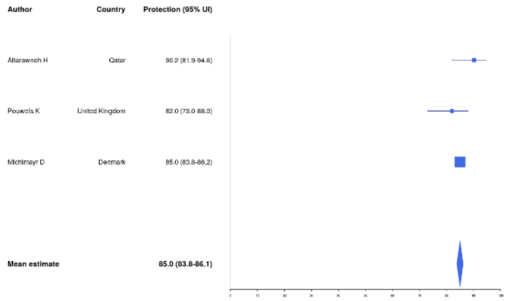
</td></tr>
<tr><td colspan="2">Omicron BA.1
z = -0.51998, p = 0.603079</td></tr>
<tr><td>Fair and Good
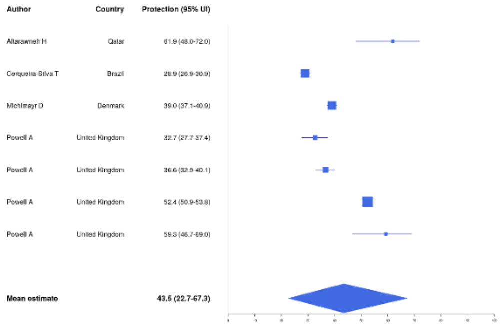
</td><td>Good
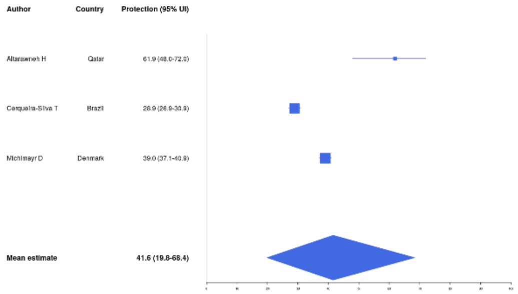
</td></tr>
<tr><td colspan="2">Protection against severe disease
Ancestral</td></tr>
<tr><td>Fair</td><td></td></tr>
</table>

<table>
<tr><td>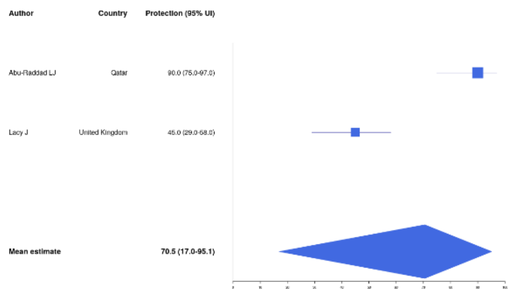
</td><td></td></tr>
<tr><th colspan="2">Alpha</th></tr>
<tr><td></td><td>Good
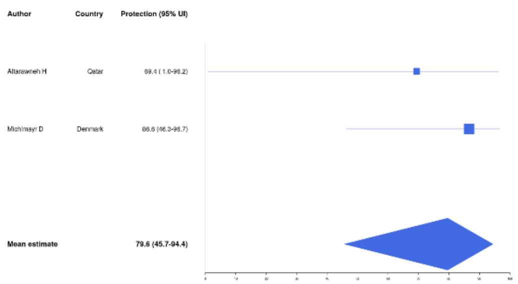
</td></tr>
<tr><th colspan="2">Beta</th></tr>
<tr><td></td><td>Good
Author: Altarawneh H
Country: Qatar
Protection (95% UI): 88.0 (50.7–97.1)</td></tr>
<tr><th colspan="2">Delta</th></tr>
<tr><td></td><td>Good
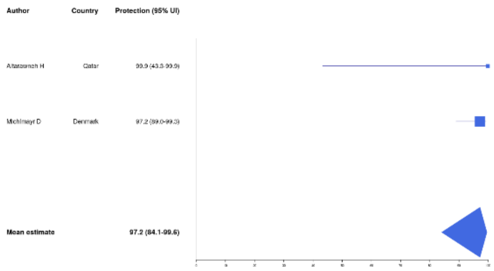
</td></tr>
<tr><th colspan="2">Omicron BA.1</th></tr>
<tr><td></td><td>Good
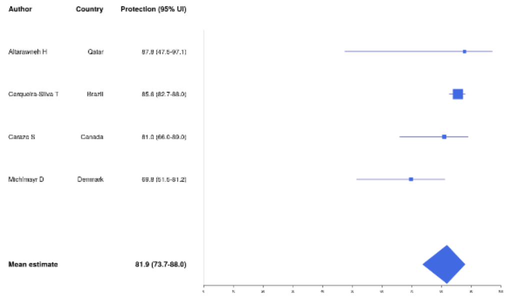
</td></tr>
</table>

Figure S3 Sensitivity analysis according to adjusted/unadjusted results

Protection against reinfection

Ancestral

z = -0.19868, p = 0.842513

No adjustment/matching

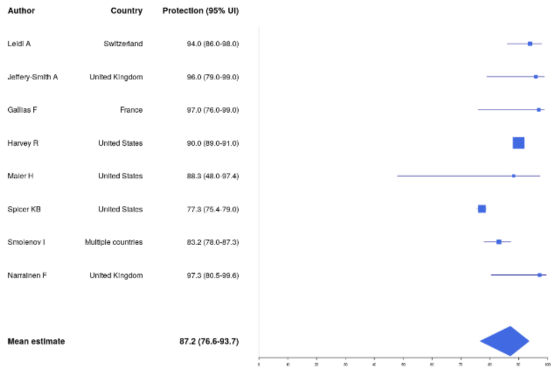

Adjusted or matched for age and sex
0 studies

Adjusted or matched for age, sex, and other variable

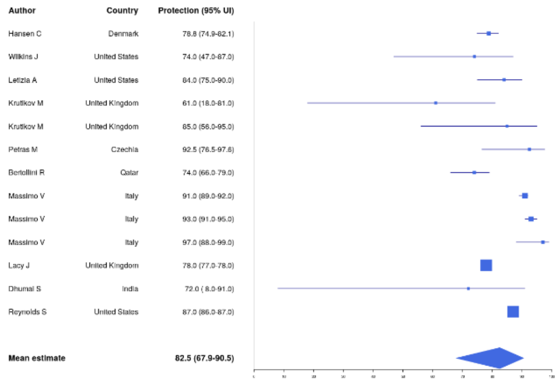

Adjusted or matched for other variables
0 studies

Alpha

z = -0.05175, p = 0.958731

No adjustment/matching

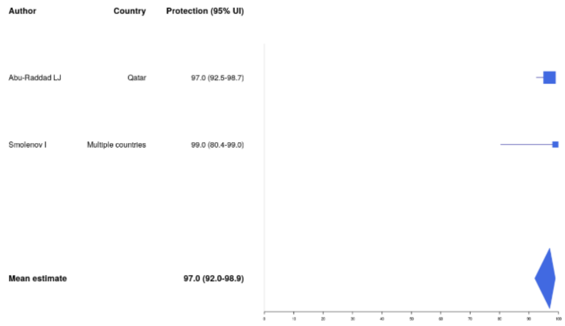

Adjusted or matched for age and sex
0 studies

Adjusted or matched for age, sex, and other variable

Adjusted or matched for other variables
0 studies

<table>
<tr><td>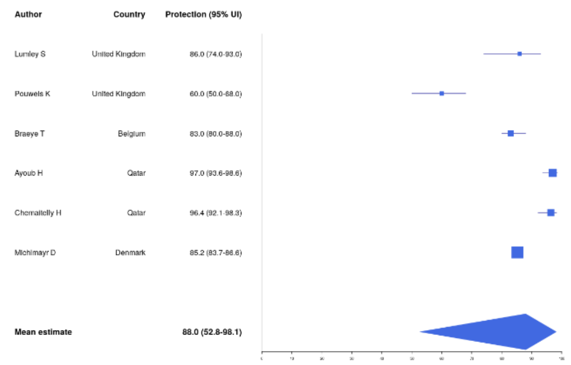
</td><td></td></tr>
<tr><td colspan="2">Beta
z = -0.06747, p = 0.946209</td></tr>
<tr><td>No adjustment/matching
Author: Smolenov I
Country: Multiple countries: Belgium, Brazil, Colombia, The Philippines, and South Africa
Protection (95% UI): 72.2 (33.1–89.9)</td><td>Adjusted or matched for age and sex
0 studies</td></tr>
<tr><td>Adjusted or matched for age, sex, and other variable
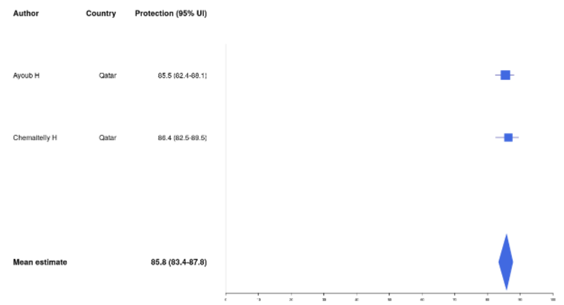
</td><td>Adjusted or matched for other variables
0 studies</td></tr>
<tr><td colspan="2">Delta
z = 0.080889, p = 0.93553</td></tr>
<tr><td>No adjustment/matching
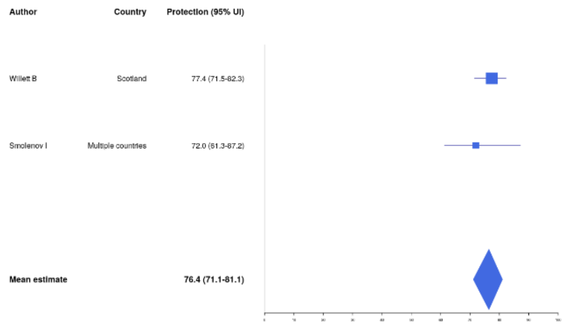
</td><td>Adjusted or matched for age and sex
0 studies</td></tr>
<tr><td>Adjusted or matched for age, sex, and other variable</td><td>Adjusted or matched for other variables
0 studies</td></tr>
</table>

<table>
<tr><td>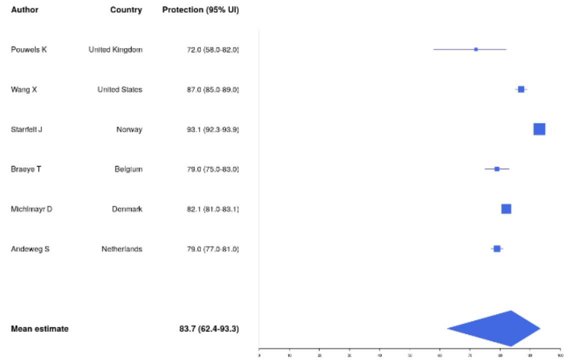
</td><td></td></tr>
<tr><td colspan="2">Omicron BA.1
z = 0.464388, p = 0.64237</td></tr>
<tr><td>No adjustment/matching
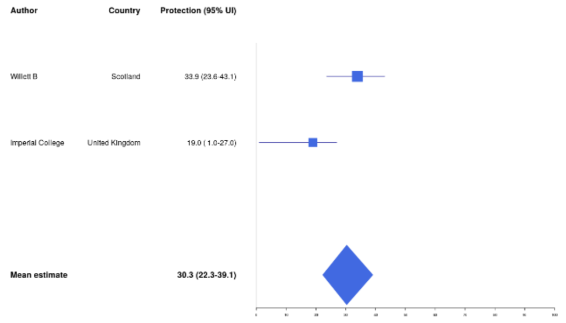
</td><td>Adjusted or matched for age and sex
0 studies</td></tr>
<tr><td>Adjusted or matched for age, sex, and other variable
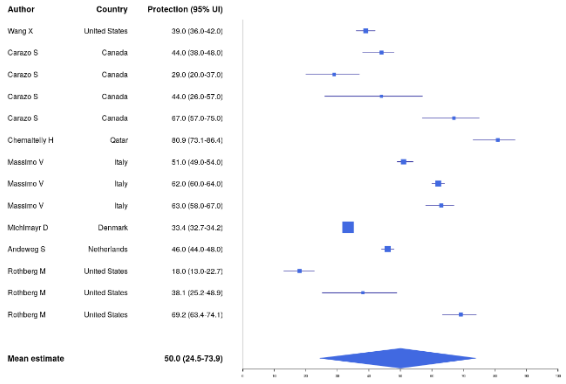
</td><td>Adjusted or matched for other variables
0 studies</td></tr>
<tr><td colspan="2">Protection against symptomatic disease</td></tr>
<tr><td colspan="2">Ancestral
z = -0.39446, p = 0.693244</td></tr>
<tr><td>No adjustment/matching</td><td>Adjusted or matched for age and sex
0 studies</td></tr>
</table>

<table>
<tr><td>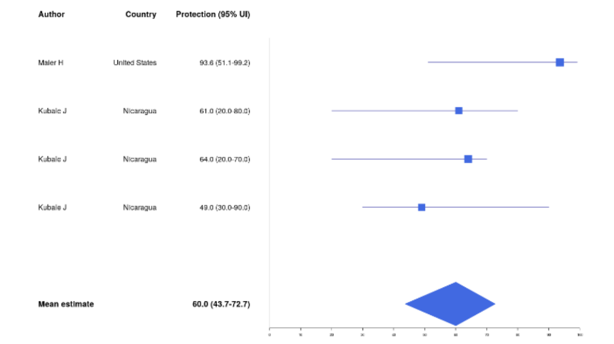
</td><td></td></tr>
<tr><td>Adjusted or matched for age, sex, and other variable
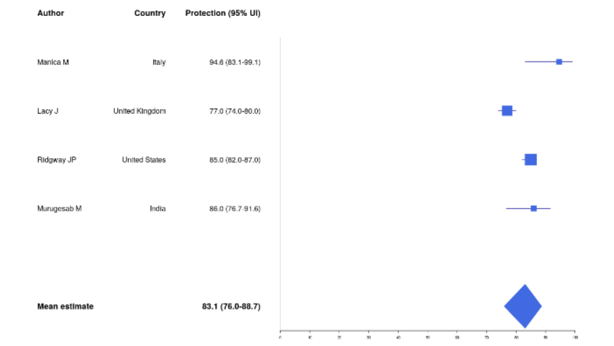
</td><td>Adjusted or matched for other variables
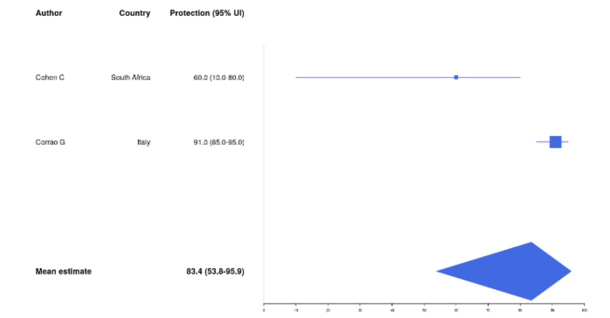
</td></tr>
<tr><td colspan="2">Alpha</td></tr>
<tr><td>No adjustment/matching
0 studies</td><td>Adjusted or matched for age and sex
0 studies</td></tr>
<tr><td>Adjusted or matched for age, sex, and other variable
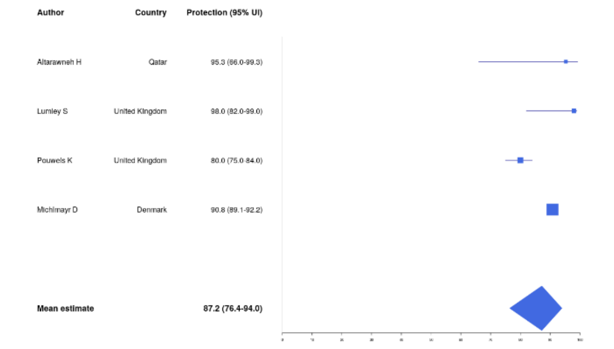
</td><td>Adjusted or matched for other variables
0 studies</td></tr>
<tr><td colspan="2">Beta</td></tr>
<tr><td>No adjustment/matching
0 studies</td><td>Adjusted or matched for age and sex
0 studies</td></tr>
<tr><td>Adjusted or matched for age, sex, and other variable
Author: Altarawneh H
Country: Qatar
Protection (95% UI): 85.4 (72.4–92.2)</td><td>Adjusted or matched for other variables
0 studies</td></tr>
<tr><td colspan="2">Delta</td></tr>
<tr><td>No adjustment/matching
0 studies</td><td>Adjusted or matched for age and sex
0 studies</td></tr>
<tr><td>Adjusted or matched for age, sex, and other variable</td><td>Adjusted or matched for other variables
0 studies</td></tr>
</table>

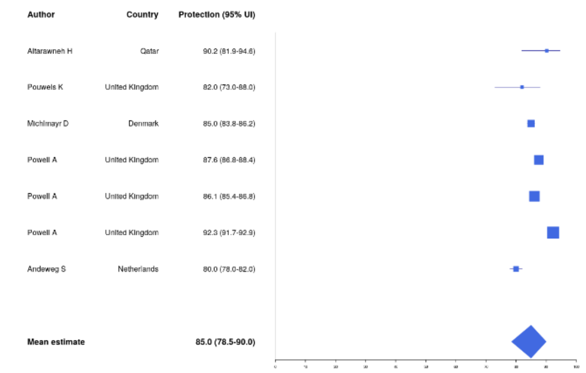

Omicron BA.1

No adjustment/matching
0 studies

Adjusted or matched for age and sex
0 studies

Adjusted or matched for age, sex, and other variable

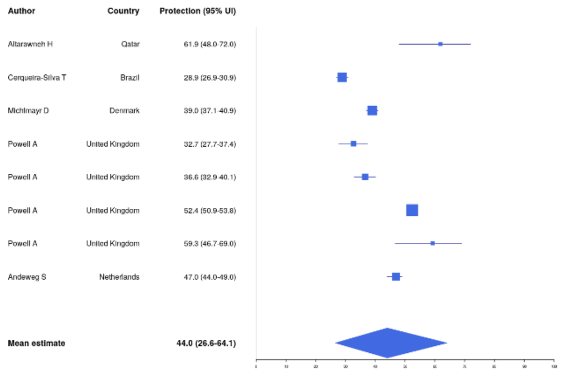

Adjusted or matched for other variables
0 studies

Protection against severe disease

Ancestral

No adjustment/matching
0 studies

Adjusted or matched for age and sex
0 studies

Adjusted or matched for age, sex, and other variable

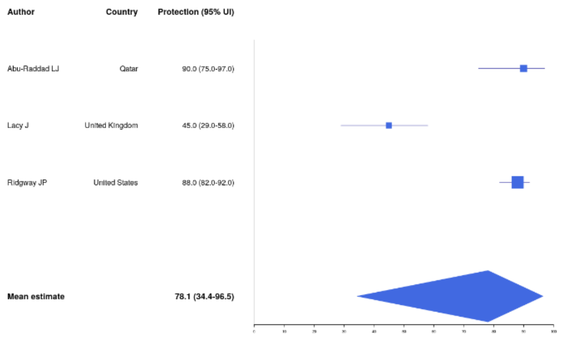

Adjusted or matched for other variables
0 studies

Alpha

No adjustment/matching
0 studies

Adjusted or matched for age and sex
0 studies

Adjusted or matched for age, sex, and other variable

Adjusted or matched for other variables
0 studies

<table>
<tr><td>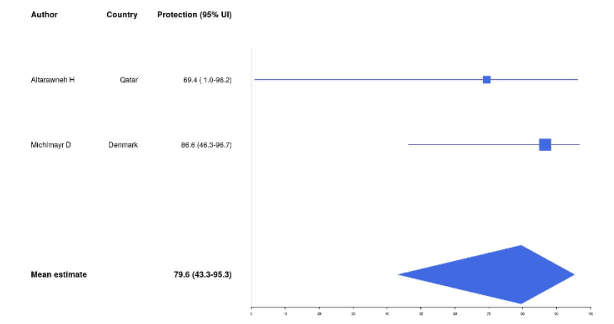
</td><td></td></tr>
<tr><td colspan="2">Beta</td></tr>
<tr><td>No adjustment/matching
0 studies</td><td>Adjusted or matched for age and sex
0 studies</td></tr>
<tr><td>Adjusted or matched for age, sex, and other variable
Author: Altarawneh H
Country: Qatar
Protection (95% UI): 88.0 (50.7–97.1)</td><td>Adjusted or matched for other variables
0 studies</td></tr>
<tr><td colspan="2">Delta</td></tr>
<tr><td>No adjustment/matching
0 studies</td><td>Adjusted or matched for age and sex
0 studies</td></tr>
<tr><td>Adjusted or matched for age, sex, and other variable
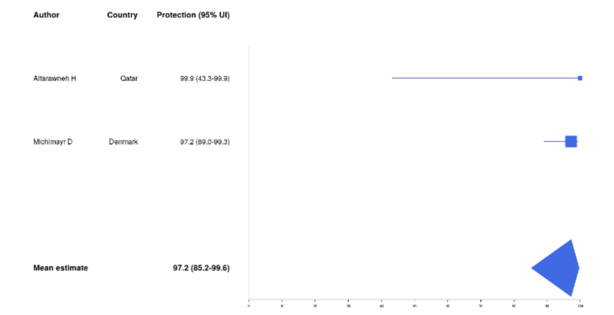
</td><td>Adjusted or matched for other variables
0 studies</td></tr>
<tr><td colspan="2">Omicron BA.1</td></tr>
<tr><td>No adjustment/matching
0 studies</td><td>Adjusted or matched for age and sex
0 studies</td></tr>
<tr><td>Adjusted or matched for age, sex, and other variable
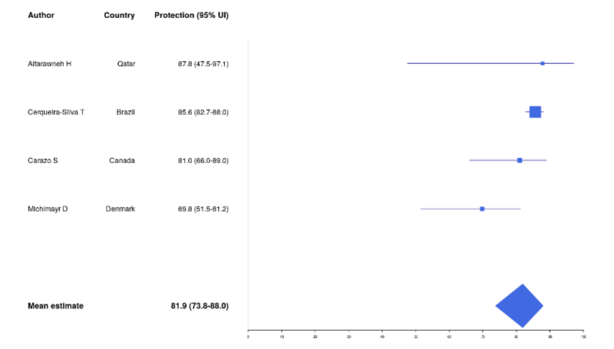
</td><td>Adjusted or matched for other variables
0 studies</td></tr>
</table>

Figure S4 Publication bias assessment

<table>
<tr><td colspan="2">Protection against reinfection</td></tr>
<tr><td>Ancestral
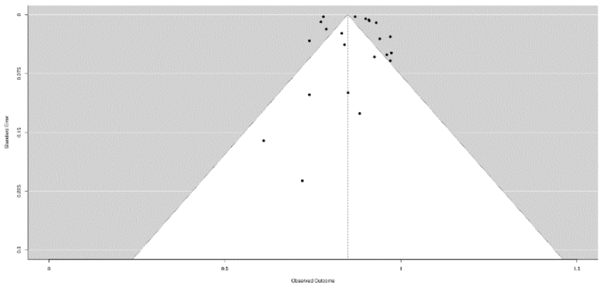
Egger's regression test for funnel plot asymmetry.
z = -0.5625, p = 0.5738</td><td>Alpha
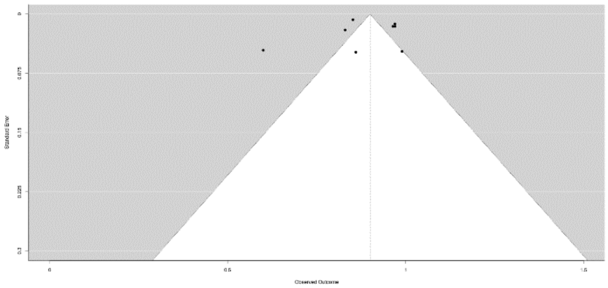
Egger's regression test for funnel plot asymmetry.
z = -1.0159, p = 0.3097</td></tr>
<tr><td>Beta
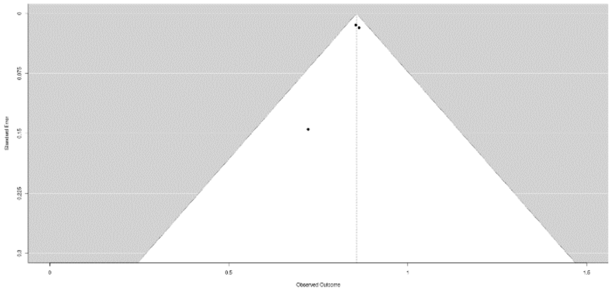
Egger's regression test for funnel plot asymmetry.
z = -0.8651, p = 0.3870</td><td>Delta
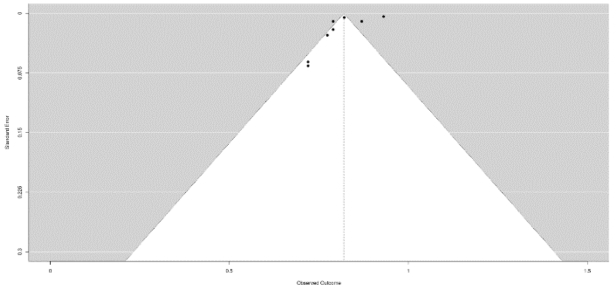
Egger's regression test for funnel plot asymmetry.
z = -2.5398, p = 0.0111</td></tr>
<tr><td>Omicron BA.1
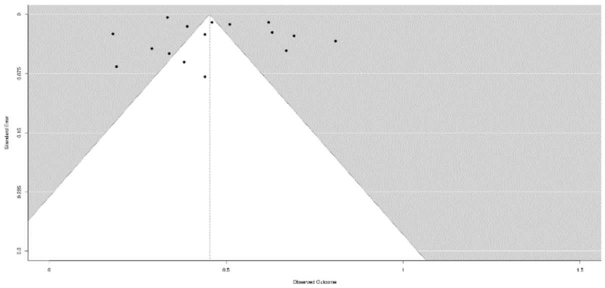
Egger's regression test for funnel plot asymmetry.
z = -0.7768, p = 0.4373</td><td></td></tr>
<tr><td colspan="2">Protection against symptomatic disease</td></tr>
<tr><td>Ancestral</td><td>Alpha</td></tr>
</table>

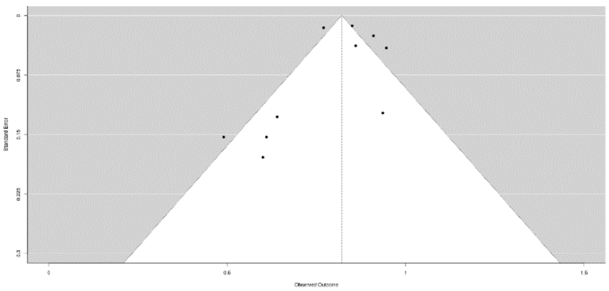<br>Egger's regression test for funnel plot asymmetry.<br>`z = -2.2226, p = 0.0262`	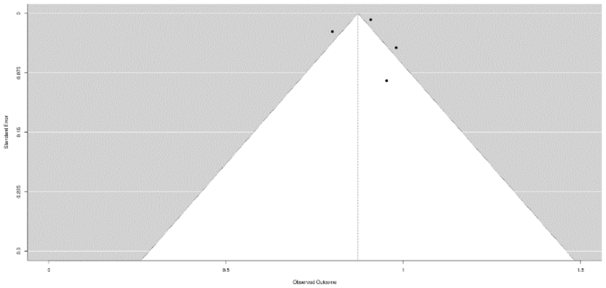<br>Egger's regression test for funnel plot asymmetry.<br>`z = 0.7302, p = 0.4653`
Beta<br>Author: Altarawneh H<br>Country: Qatar<br>Protection (95% UI): 85.4 (72.4–92.2)	Delta<br>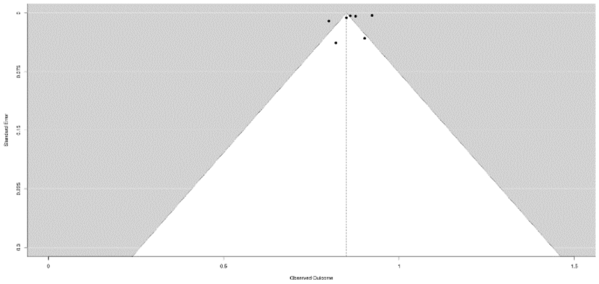<br>Egger's regression test for funnel plot asymmetry.<br>`z = -0.4368, p = 0.6622`
Omicron BA.1<br>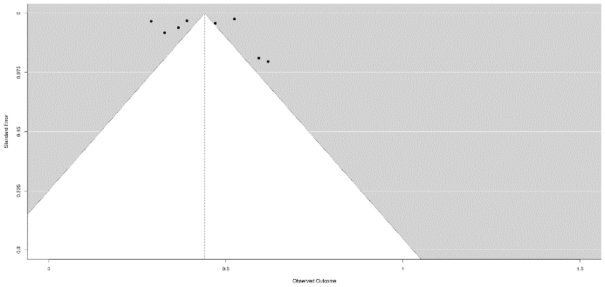<br>Egger's regression test for funnel plot asymmetry.<br>`z = 2.0055, p = 0.0449`	
Protection against severe disease	
Ancestral<br>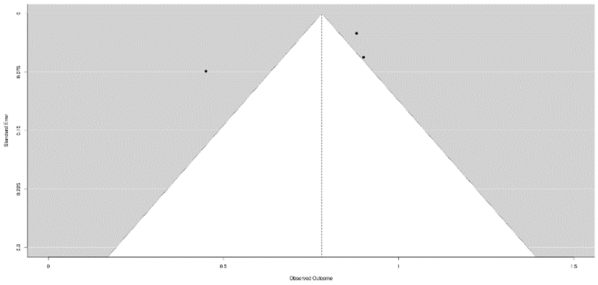<br>Egger's regression test for funnel plot asymmetry.<br>`z = -1.1403, p = 0.2542`	Alpha<br>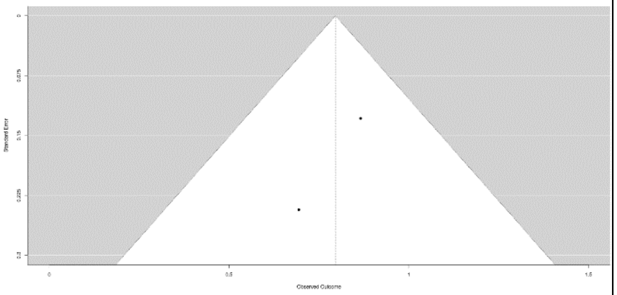<br>Egger's regression test for funnel plot asymmetry.<br>Number of parameters to be estimated is larger than the number of observations.
Beta<br>Author: Altarawneh H	Delta

Country: Qatar<br>Protection (95% UI): 88.0 (50.7–97.1)	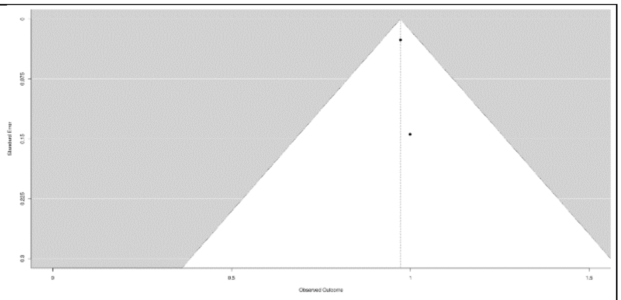<br>Egger's regression test for funnel plot asymmetry.<br>Number of parameters to be estimated is larger than the number of observations.
Omicron BA.1<br>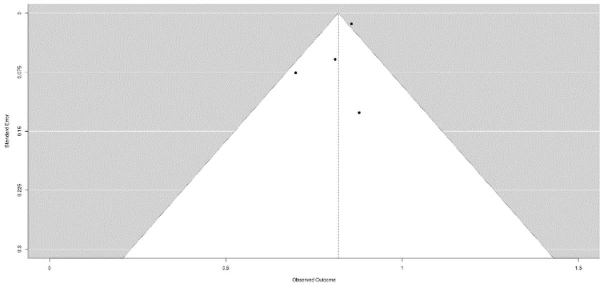<br>Egger's regression test for funnel plot asymmetry.<br>`z = -1.4175, p = 0.1563`	

Table S1 **Data inputs and characteristics of included studies**

First author name	Study design	Age or median age	Country	Variant (1st infection)	Variant (2nd infection)	Outcome	Parameter	Adjustment	Mean efficacy	Lower 95% CI	Upper 95% CI	Interval or mean time since infection (in weeks)
Berec L[3]	retrospective cohort	>12	Czechia	mixed variant	all variant	infection	1 - aHR	Age and sex	0.99	0.98	0.99	0-8
				mixed variant	all variant	infection	1 - aHR		0.97	0.965	0.975	9-16
				mixed variant	all variant	infection	1 - aHR		0.91	0.905	0.915	17-24
				mixed variant	all variant	infection	1 - aHR		0.92	0.915	0.925	25-32
				mixed variant	all variant	infection	1 - aHR		0.9	0.895	0.905	33-40
				mixed variant	all variant	infection	1 - aHR		0.84	0.835	0.845	41-48
				mixed variant	all variant	infection	1 - aHR		0.79	0.78	0.8	49-56
				mixed variant	all variant	infection	1 - aHR		0.71	0.685	0.72	57-64
				mixed variant	all variant	infection	1 - aHR		0.71	0.65	0.78	65-72
Hansen C[4]	retrospective cohort	>18	Denmark	Ancestral	Ancestral	infection	1 - aRR	Age, sex, and other covariates	0.788	0.749	0.821	NA
				Ancestral	Ancestral	infection	1 - aRR		0.793	0.744	0.833	12_24
				Ancestral	Ancestral	infection	1 - aRR		0.777	0.709	0.829	25_32
Altarawneh H[5]	test-negative case-control	All ages	Qatar	Ancestral	Alpha	symptomatic	1 - aOR	Age, sex, and other covariates	0.953	0.66	0.993	NA
			Qatar	Ancestral	Beta	symptomatic	1 - aOR		0.854	0.724	0.922	NA
			Qatar	mixed variant	Delta	symptomatic	1 - aOR		0.902	0.819	0.946	NA

			Qatar	mixed variant	Omicron (unspecified sub-lineage)	symptomatic	1 - aOR		0.619	0.48	0.72	NA
			Qatar	Ancestral	Alpha	hospitalisation & death	1 - aOR		0.694	0.01	0.962	NA
			Qatar	Ancestral	Beta	hospitalisation & death	1 - aOR		0.88	0.507	0.971	NA
			Qatar	mixed variant	Delta	hospitalisation & death	1 - aOR		0.999	0.433	0.999	NA
			Qatar	mixed variant	Omicron (unspecified sub-lineage)	hospitalisation & death	1 - aOR		0.878	0.475	0.971	NA
			Qatar	Ancestral	Alpha	infection	1 - aOR		0.894	0.226	0.985	12_32
			Qatar	Ancestral	Alpha	infection	1 - aOR		0.91	0.345	0.988	33_56
			Qatar	Ancestral	Beta	infection	1 - aOR		0.926	0.767	0.976	12_32
			Qatar	Ancestral	Beta	infection	1 - aOR		0.812	0.655	0.898	33_56
			Qatar	mixed variant	Delta	infection	1 - aOR		0.934	0.876	0.965	12_32
			Qatar	mixed variant	Delta	infection	1 - aOR		0.911	0.833	0.953	33_56
			Qatar	mixed variant	Delta	infection	1 - aOR		0.871	0.594	0.959	>56
			Qatar	mixed variant	Omicron (unspecified sub-lineage)	infection	1 - aOR		0.64	0.547	0.714	12_32
			Qatar	mixed variant	Omicron (unspecified sub-lineage)	infection	1 - aOR		0.472	0.375	0.554	33_56

			Qatar	mixed variant	Omicron (unspecified sub-lineage)	infection	1 - aOR		0.596	0.507	0.67	>56
Rane M[6]	test-negative case-control	>12	US	mixed variant	Delta	Symptomatic	1 - aOR	Age, sex, and other covariates	0.9	0.87	0.93	34*
Smid M[7]	retrospective cohort	>5	Czechia	mixed variant	Omicron (unspecified sub-lineage)	hospitalisation	1 - aHR	Age and sex	0.87	0.73	0.94	2_24
				mixed variant	Omicron (unspecified sub-lineage)	hospitalisation	1 - aHR		0.92	0.86	0.96	>24
				mixed variant	Delta	hospitalisation	1 - aHR		0.99	0.98	0.99	2_24
				mixed variant	Delta	hospitalisation	1 - aHR		0.95	0.93	0.96	>24
				mixed variant	Omicron (unspecified sub-lineage)	infection	1 - aHR		0.68	0.68	0.69	2_24
				mixed variant	Omicron (unspecified sub-lineage)	infection	1 - aHR		0.13	0.1	0.14	>24
				mixed variant	Delta	infection	1 - aHR		0.95	0.94	0.96	2_24
				mixed variant	Delta	infection	1 - aHR		0.83	0.82	0.84	>24
Abu-Raddad L[8]	retrospective cohort	10-74	Qatar	Ancestral	Ancestral	infection	1 - aOR	Other covariates	0.927	0.913	0.939	17*
Hall V[9]	prospective cohort	>18	UK	Ancestral	Alpha	infection	1 - aIRR	Age and sex	0.84	0.81	0.87	29*
Hall V[10]	prospective cohort	>25	UK	all variant	all variant	infection	1 - aHR	Other covariates	0.86	0.81	0.89	0_51

				all variant	all variant	infection	1 - aHR		0.69	0.38	0.84	52_104
Pilz S[11]	retrospective cohort	15-93	Austria	Ancestral	Ancestral	infection	1 - OR	No adjustment	0.91	0.87	0.93	30*
Rennert L[12]	retrospective cohort	18-24	US	Ancestral	mixed variant	infection	1 - aRR	Age, sex, and other covariates	0.84	0.78	0.88	18*
Sheehan M[13]	retrospective cohort	18-80	US	Ancestral	Ancestral	infection	1 - OR	No adjustment	0.818	0.766	0.858	20*
			US	Ancestral	Ancestral	Symptomatic	1 - OR		0.845	0.779	0.891	20*
Vitale J[14]	retrospective cohort	18-80	Italy	Ancestral	Ancestral	infection	1 - aHR	Age, sex, and other covariates	0.94	0.92	0.95	34*
Wilkins J[15]	prospective cohort	>18	US	Ancestral	Ancestral	infection	1 - aIRR	Age, sex, and other covariates	0.74	0.47	0.87	NA
Letizia A[16]	prospective cohort	18-20	US	Ancestral	Ancestral	infection	1 - aOR	Age, sex, and other covariates	0.84	0.75	0.9	NA
Abo-Leyah H[17]	prospective cohort	>18	UK	Ancestral	Ancestral	Symptomatic	1 - OR	No adjustment	0.85	0.65	0.94	11*
Lumley S[18]	longitudinal cohort	39*	UK	Ancestral	Alpha	Symptomatic	1 - aIRR	Age, sex, and other covariates	0.98	0.82	0.99	NA
			UK	Ancestral	Alpha	infection	1 - aIRR		0.86	0.74	0.93	NA
Breathnach A[19]	retrospective cohort	50*	UK	Ancestral	Ancestral	infection	1 - RR	No adjustment	0.94	0.88	0.97	32*
Manica M[20]	retrospective cohort	50*	Italy	Ancestral	Ancestral	Symptomatic	1 - aOR	Age, sex, and other covariates	0.946	0.831	0.991	NA
Leidi A[21]	retrospective cohort	>12	Switzerland	Ancestral	Ancestral	infection	1 - HR	No adjustment	0.94	0.86	0.98	NA
Pouwels K[22]	prospective cohort	>18	UK	Ancestral	Alpha	Symptomatic	1 - aOR	Age, sex, and other covariates	0.8	0.75	0.84	NA
				Ancestral	Alpha	infection	1 - aOR		0.6	0.5	0.68	NA

				Ancestral and Alpha	Delta	Symptomatic	1 - aOR		0.82	0.73	0.88	NA
				Ancestral and Alpha	Delta	infection	1 - aOR		0.72	0.58	0.82	NA
Pritchard E[23]	prospective cohort	>18	UK	Ancestral and Alpha	Ancestral and Alpha	infection	1 - aOR	Age, sex, and other covariates	0.28	0.1	0.43	0_15
				Ancestral and Alpha	Ancestral and Alpha	infection	1 - aOR		0.7	0.56	0.79	16_32
				Ancestral and Alpha	Ancestral and Alpha	Symptomatic	1 - aOR		0.67	0.5	0.88	0_15
				Ancestral and Alpha	Ancestral and Alpha	Symptomatic	1 - aOR		0.95	0.85	0.985	16_32
				Ancestral and Alpha	Alpha	infection	1 - aOR		0.42	0.19	0.58	0_15
				Ancestral and Alpha	Alpha	infection	1 - aOR		0.81	0.66	0.9	16_32
Wang X[24]	test-negative case-control	All ages	US	mixed variant	Delta	infection	1 - aOR	Age, sex, and other covariates	0.87	0.85	0.89	NA
				mixed variant	Omicron (unspecified sub-lineage)	infection	1 - aOR		0.39	0.36	0.42	NA
				mixed variant	Omicron (unspecified sub-lineage)	infection	1 - OR		0.339	0.236	0.431	NA
Willett B[25]	test-negative case-control	18-108	Scotland	mixed variant	Delta	infection	1 - OR	No adjustment	0.774	0.715	0.823	NA
				mixed variant	Omicron (unspecif	infection	1 - OR		0.339	0.236	0.431	NA

					ied sub-lineage)							
Altarawneh H[26]	test-negative case-control	All ages	Qatar	mixed variant	Omicron (unspecified sub-lineage)	Symptomatic	1 - OR	Age, sex, and other covariates	0.508	0.454	0.557	46*
				mixed variant	Omicron BA.1	Symptomatic	1 - OR		0.505	0.381	0.599	46*
				mixed variant	Omicron BA.2	Symptomatic	1 - OR		0.461	0.395	0.519	46*
				mixed variant	Omicron (unspecified sub-lineage)	hospitalisation	1 - OR		0.716	0.157	0.904	46*
				mixed variant	Omicron BA.2	hospitalisation	1 - OR		0.734	0.002	0.929	46*
Starrfelt J[27]	retrospective cohort	>18	Norway	mixed variant	Delta	infection	1 - aHR	Age, sex, and other covariates	0.931	0.923	0.939	NA
Cerqueira-Silva T[28]	test-negative case-control	>18	Brazil	mixed variant	Omicron (unspecified sub-lineage)	Symptomatic	1 - aOR	Age, sex, and other covariates	0.289	0.269	0.309	NA
				mixed variant	Omicron (unspecified sub-lineage)	hospitalisation & death	1 - aOR		0.856	0.827	0.88	NA
				mixed variant	Omicron (unspecified sub-lineage)	hospitalisation & death	1 - aOR		0.845	0.731	0.911	12_23
				mixed variant	Omicron (unspecified sub-lineage)	hospitalisation & death	1 - aOR		0.895	0.86	0.922	24_51
				mixed variant	Omicron (unspecified sub-lineage)	hospitalisation & death	1 - aOR		0.803	0.744	0.848	>52

				mixed variant	Omicron (unspecified sub-lineage)	Symptomatic	1 - aOR		0.528	0.483	0.568	12_23
				mixed variant	Omicron (unspecified sub-lineage)	Symptomatic	1 - aOR		0.327	0.302	0.352	24_51
				mixed variant	Omicron (unspecified sub-lineage)	Symptomatic	1 - aOR		0.147	0.108	0.185	>52
Carazo S[29]	test-negative case-control	>12	Canada	mixed variant	Omicron (unspecified sub-lineage)	infection	1 - aOR	Age, sex, and other covariates	0.44	0.38	0.48	NA
				Ancestral	Omicron (unspecified sub-lineage)	infection	1 - aOR		0.29	0.2	0.37	NA
				Alpha	Omicron (unspecified sub-lineage)	infection	1 - aOR		0.44	0.26	0.57	NA
				Delta	Omicron (unspecified sub-lineage)	infection	1 - aOR		0.67	0.57	0.75	NA
				mixed variant	Omicron (unspecified sub-lineage)	hospitalisation	1 - aOR		0.81	0.66	0.89	NA
				mixed variant	Omicron (unspecified sub-lineage)	infection	1 - aOR		0.66	0.57	0.73	12_23
				mixed variant	Omicron (unspecified sub-lineage)	infection	1 - aOR		0.49	0.32	0.61	24_35

				Ancestral	Omicron (unspecified sub-lineage)	infection	1 - aOR		0.35	0.21	0.47	36_47
				Delta	Omicron (unspecified sub-lineage)	infection	1 - aOR		0.29	0.17	0.38	48_75
				Alpha	Omicron (unspecified sub-lineage)	infection	1 - aOR		0.27	0.08	0.42	76_96
Nordstrom P[30]	retrospective cohort	39.9*	Sweden	mixed variant	mixed variant	infection	1 - aHR	Age, sex, and other covariates	0.48	0.47	0.48	2_12
				mixed variant	mixed variant	infection	1 - aHR		0.96	0.95	0.96	13_24
				mixed variant	mixed variant	infection	1 - aHR		0.92	0.91	0.93	25_36
				mixed variant	mixed variant	infection	1 - aHR		0.93	0.92	0.98	>36
				mixed variant	mixed variant	hospitalisation	1 - aHR		0.89	0.86	0.91	13_24
				mixed variant	mixed variant	hospitalisation	1 - aHR		0.85	0.09	0.78	25_36
				mixed variant	mixed variant	hospitalisation	1 - aHR		0.78	0.085	0.66	>36
Office of National Statistics[31]	prospective cohort	>2	UK	mixed variant	mixed variant	infection	NA	NA	0.909	0.894	0.92	NA
Imperial College[32]	test-negative case-control	NA	UK	mixed variant	Omicron (unspecified sub-lineage)	infection	1 - RR	No adjustment	0.19	0	0.27	NA
Jeffery-Smith A[33]	prospective cohort	NA	UK	Ancestral	Ancestral	infection	1 - RR	No adjustment	0.96	0.79	0.99	NA
Gallais F[34]	prospective cohort	39*	France	Ancestral	mixed variant	infection	1 - HR	No adjustment	0.97	0.76	0.99	NA

Krutikov M[35]	prospective cohort	18-64	UK	Ancestral	mixed variant	infection	1 - aHR	Age, sex, and other covariates	0.61	0.18	0.81	NA
		>64	UK	Ancestral	mixed variant	infection	1 - aHR		0.85	0.56	0.95	NA
Harvey R[36]	retrospective cohort	48*	US	Ancestral	Ancestral	infection	1 - RR	No adjustment	0.9	0.89	0.91	NA
Maier H[37]	prospective cohort	0-96	US	mixed variant	mixed variant	infection	1 - OR	No adjustment	0.883	0.48	0.974	NA
			US	mixed variant	mixed variant	Symptomatic	1 - OR		0.936	0.511	0.992	NA
Chemaitelly H[38]	retrospective cohort	All ages	Qatar	Omicron BA.1	Omicron BA.2	infection	1 - aHR	Age, sex, and other covariates	0.942	0.892	0.969	NA
				Omicron BA.2	Omicron BA.1	infection	1 - aHR		0.809	0.731	0.864	NA
Satwik R[39]	retrospective cohort	NA	India	mixed variant	Delta	Symptomatic	1 - aHR	Age, sex, and other covariates	0.93	0.87	0.96	38*
Chin E[40]	test-negative case-control	>18	US	mixed variant	Omicron (unspecified sub-lineage)	infection	1 - aOR	Age, sex, and other covariates	0.257	0.234	0.316	59*
				Delta	Omicron (unspecified sub-lineage)	infection	1 - aOR		0.608	0.584	0.668	21*
Braeye T[41]	retrospective cohort	0-84	Belgium	mixed variant	Delta	infection	1 - aOR	Age, sex, and other covariates	0.79	0.75	0.83	NA
				mixed variant	Alpha	infection	1 - aOR		0.83	0.8	0.88	NA
				mixed variant	Delta	infection	1 - aOR		0.79	0.74	0.83	0_7
				mixed variant	Delta	infection	1 - aOR		0.64	0.61	0.66	21_28
Ayoub H[42]	test-negative case-control	All ages	Qatar	mixed variant	Alpha	infection	1 - aOR	Age, sex, and other covariates	0.97	0.936	0.986	NA
				mixed variant	Beta	infection	1 - aOR		0.855	0.824	0.881	NA

Chemaitelly H[43]	retrospective cohort	All ages	Qatar	mixed variant	Alpha	infection	1 - aHR	Age, sex, and other covariates	0.964	0.921	0.983	NA
				mixed variant	Beta	infection	1 - aHR		0.864	0.825	0.895	NA
Petras M[44]	retrospective cohort	All ages	Czechia	mixed variant	mixed variant	infection	1 - aIRR	Age, sex, and other covariates	0.925	0.765	0.976	NA
Carazo S[29]	test-negative case-control	>18	Canada	mixed variant	Omicron BA.2	infection	1 - aOR	Age, sex, and other covariates	NA	NA	NA	4_7
				mixed variant	Omicron BA.2	infection	1 - aOR		NA	NA	NA	8_11
				mixed variant	Omicron BA.2	infection	1 - aOR		0.42	-0.47	0.77	12_23
				mixed variant	Omicron BA.2	infection	1 - aOR		0.39	0	0.63	24_51
				mixed variant	Omicron BA.2	infection	1 - aOR		0.42	0.17	0.6	>51
				Omicron BA.1	Omicron BA.2	infection	1 - aOR		0.82	0.49	0.94	4_7
				Omicron BA.1	Omicron BA.2	infection	1 - aOR		0.76	0.63	0.85	8_11
				Omicron BA.1	Omicron BA.2	infection	1 - aOR		0.7	0.61	0.77	12_23
				Omicron BA.1	Omicron BA.2	infection	1 - aOR		NA	NA	NA	24_51
				Omicron BA.1	Omicron BA.2	infection	1 - aOR		NA	NA	NA	>51
Altarawneh H[45]	test-negative case-control	All ages	Qatar	mixed variant	Omicron BA.4/BA.5	infection	1 - OR	Age, sex, and other covariates	0.286	0.118	0.422	NA
				mixed variant	Omicron BA.4/BA.5	Symptomatic	1 - OR		0.149	-0.475	0.509	NA
				Omicron (unspecified sub-lineage)	Omicron BA.4/BA.5	infection	1 - OR		0.797	0.744	0.84	NA

				Omicron (unspecified sub-lineage)	Omicron BA.4/BA.5	Symptomatic	1 - OR		0.761	0.549	0.873	NA
Abu-Raddad LJ[46]	retrospective cohort	All ages	Qatar	Ancestral	Alpha	infection	1 - IRR	No adjustment	0.975	0.957	0.986	NA
Bertollini R[47]	retrospective cohort	33*	Qatar	mixed variant	mixed variant	infection	1- RR	Age, sex, and other covariates	0.74	0.66	0.79	NA
Chemaitelly H[48]	retrospective cohort	All ages	Qatar	mixed variant	mixed variant	infection	1 - aHR	Age, sex, and other covariates	0.806	0.778	0.831	0-16
			Qatar	mixed variant	mixed variant	infection	1 - aHR		0.863	0.838	0.887	17-20
			Qatar	mixed variant	mixed variant	infection	1 - aHR		0.887	0.862	0.909	21-24
			Qatar	mixed variant	mixed variant	infection	1 - aHR		0.905	0.881	0.926	25-28
			Qatar	mixed variant	mixed variant	infection	1 - aHR		0.9	0.877	0.922	29-32
			Qatar	mixed variant	mixed variant	infection	1 - aHR		0.888	0.866	0.909	33-36
			Qatar	mixed variant	mixed variant	infection	1 - aHR		0.864	0.838	0.885	37-40
			Qatar	mixed variant	mixed variant	infection	1 - aHR		0.824	0.795	0.851	41-44
			Qatar	mixed variant	mixed variant	infection	1 - aHR		0.754	0.704	0.799	45-48
			Qatar	mixed variant	mixed variant	infection	1 - aHR		0.785	0.696	0.851	49-52
			Qatar	mixed variant	mixed variant	infection	1 - aHR		0.755	0.577	0.862	53-56
			Qatar	mixed variant	mixed variant	infection	1 - aHR		0.751	0.538	0.87	57-60
			Qatar	mixed variant	mixed variant	infection	1 - aHR		0.716	0.422	0.862	>60
			Qatar	Ancestral	Omicron (unspecif	infection	1 - aHR		0.381	0.356	0.406	48*

					ied sub-lineage)							
			Qatar	Ancestral	Omicron (unspecified sub-lineage)	infection	1 - aHR		0.484	0.46	0.507	46*
			Qatar	Ancestral	Omicron (unspecified sub-lineage)	infection	1 - aHR		0.519	0.488	0.545	62*
			Qatar	Alpha	Omicron (unspecified sub-lineage)	infection	1 - aHR		0.17	0.146	0.193	32*
			Qatar	Beta	Omicron (unspecified sub-lineage)	infection	1 - aHR		0.317	0.297	0.34	24*
			Qatar	Delta	Omicron (unspecified sub-lineage)	infection	1 - aHR		0.571	0.552	0.598	8*
			Qatar	mixed variant	mixed variant	hospitalisation & death	1 - aHR		0.934	0.783	0.982	0-16
			Qatar	mixed variant	mixed variant	hospitalisation & death	1 - aHR		0.99	0.904	0.99	17-20
			Qatar	mixed variant	mixed variant	hospitalisation & death	1 - aHR		0.99	0.904	0.99	21-24
			Qatar	mixed variant	mixed variant	hospitalisation & death	1 - aHR		0.943	0.567	0.996	25-28
			Qatar	mixed variant	mixed variant	hospitalisation & death	1 - aHR		0.99	0.902	0.99	29-32
			Qatar	mixed variant	mixed variant	hospitalisation & death	1 - aHR		0.942	0.81	0.985	33-36

			Qatar	mixed variant	mixed variant	hospitalisation & death	1 - aHR		0.981	0.859	0.99	37-40
			Qatar	mixed variant	mixed variant	hospitalisation & death	1 - aHR		0.98	0.852	0.99	41-44
			Qatar	mixed variant	mixed variant	hospitalisation & death	1 - aHR		0.99	0.835	0.99	45-48
			Qatar	mixed variant	mixed variant	hospitalisation & death	1 - aHR		0.99	0	0.99	49-52
			Qatar	mixed variant	mixed variant	hospitalisation & death	1 - aHR		0.952	0.516	0.999	53-62
Vicentini M[49]	retrospective cohort	All ages	Italy	Ancestral	mixed variant	infection	1 - aHR	Age, sex, and other covariates	0.91	0.89	0.92	NA
			Italy	Alpha	mixed variant	infection	1 - aHR		0.93	0.91	0.95	NA
			Italy	Delta	mixed variant	infection	1 - aHR		0.97	0.88	0.99	NA
			Italy	Ancestral	Omicron (unspecified sub-lineage)	infection	1 - aHR		0.51	0.49	0.54	NA
			Italy	Alpha	Omicron (unspecified sub-lineage)	infection	1 - aHR		0.62	0.64	0.6	NA
			Italy	Delta	Omicron (unspecified sub-lineage)	infection	1 - aHR		0.63	0.58	0.67	NA
Abu-Raddad LJ[50]	retrospective cohort	All ages	Qatar	mixed variant	mixed variant	hospitalisation & death	1 - aOR	Age, sex, and other covariates	0.9	0.75	0.97	NA
Michlmayr D[51]	retrospective cohort	>2	Denmark	mixed variant	Alpha	infection	1 - aHR	Age, sex, and other covariates	0.852	0.837	0.866	NA
				mixed variant	Delta	infection	1 - aHR		0.821	0.81	0.831	NA

				mixed variant	Omicron (unspecif ied sub-lineage)	infection	1 - aHR		0.334	0.32 7	0.34 2	NA
				mixed variant	Alpha	Symptom atic	1 - aHR		0.908	0.89 1	0.92 2	NA
				mixed variant	Delta	Symptom atic	1 - aHR		0.85	0.83 8	0.86 2	NA
				mixed variant	Omicron (unspecif ied sub-lineage)	Symptom atic	1 - aHR		0.39	0.37 1	0.40 9	NA
				mixed variant	Alpha	hospitalis ation	1 - aHR		0.866	0.46 3	0.96 7	NA
				mixed variant	Delta	hospitalis ation	1 - aHR		0.972	0.89	0.99 3	NA
				mixed variant	Omicron (unspecif ied sub-lineage)	hospitalis ation	1 - aHR		0.698	0.51 5	0.81 2	NA
				mixed variant	Alpha	infection	1 - aHR		0.867	0.84 9	0.88 3	12-25
				mixed variant	Alpha	infection	1 - aHR		0.833	0.80 1	0.86	26-38
				mixed variant	Alpha	infection	1 - aHR		0.702	0.56 8	0.79 4	39-51
				mixed variant	Alpha	infection	1 - aHR		0.864	0.76 1	0.92 3	>51
				mixed variant	Delta	infection	1 - aHR		0.913	0.89 7	0.92 7	12-25
				mixed variant	Delta	infection	1 - aHR		0.84	0.82 2	0.85 6	26-38
				mixed variant	Delta	infection	1 - aHR		0.767	0.74 6	0.78 7	39-51
				mixed variant	Delta	infection	1 - aHR		0.714	0.66 9	0.75 3	>51
				mixed variant	Omicron (unspecif	infection	1 - aHR		0.51	0.50 1	0.52	12-25

					ied sub-lineage)							
				mixed variant	Omicron (unspecified sub-lineage)	infection	1 - aHR		0.254	0.233	0.273	26-38
				mixed variant	Omicron (unspecified sub-lineage)	infection	1 - aHR		0.251	0.231	0.271	39-51
				mixed variant	Omicron (unspecified sub-lineage)	infection	1 - aHR		0.19	0.172	0.205	>51
				mixed variant	Alpha	Symptomatic	1 - aHR		0.926	0.907	0.94	12-25
				mixed variant	Alpha	Symptomatic	1 - aHR		0.89	0.856	0.916	26-38
				mixed variant	Alpha	Symptomatic	1 - aHR		0.72	0.546	0.827	39-51
				mixed variant	Alpha	Symptomatic	1 - aHR		0.932	0.819	0.975	>51
				mixed variant	Delta	Symptomatic	1 - aHR		0.931	0.912	0.945	12-25
				mixed variant	Delta	Symptomatic	1 - aHR		0.872	0.853	0.89	26-38
				mixed variant	Delta	Symptomatic	1 - aHR		0.8	0.774	0.822	39-51
				mixed variant	Delta	Symptomatic	1 - aHR		0.747	0.694	0.79	>51
				mixed variant	Omicron (unspecified sub-lineage)	Symptomatic	1 - aHR		0.551	0.533	0.568	12-25
				mixed variant	Omicron (unspecified sub-lineage)	Symptomatic	1 - aHR		0.311	0.279	0.34	26-38
				mixed variant	Omicron (unspecif	Symptomatic	1 - aHR		0.314	0.284	0.343	39-51

					ied sub-lineage)							
				mixed variant	Omicron (unspecified sub-lineage)	Symptomatic	1 - aHR		0.257	0.228	0.284	>51
Lacy J[52]	retrospective cohort	>10	UK	Ancestral	mixed variant	infection	1 - OR	Age, sex, and other covariates	0.78	0.77	0.78	NA
				Ancestral	mixed variant	Symptomatic	1 - OR		0.77	0.74	0.8	NA
				Ancestral	mixed variant	death	1 - aOR		0.45	0.29	0.58	NA
Ridgway JP[53]	retrospective cohort	40*	US	Ancestral	mixed variant	Symptomatic	1 - aHR	Age, sex, and other covariates	0.85	0.82	0.87	NA
				Ancestral	mixed variant	hospitalisation	1 - aHR		0.88	0.82	0.92	NA
				Ancestral	mixed variant	Symptomatic	1 - aHR		0.85	0.79	0.9	12-14
				Ancestral	mixed variant	Symptomatic	1 - aHR		0.86	0.8	0.91	15-21
				Ancestral	mixed variant	Symptomatic	1 - aHR		0.86	0.78	0.91	22-28
				Ancestral	mixed variant	Symptomatic	1 - aHR		0.88	0.8	0.95	29-35
Spicer KB[54]	Retrospective cohort	>20	US	Mixed variant	Mixed variant	Infection	1 – RR	No adjustment	0.77	0.75	0.79	NA
				Mixed variant	Mixed variant	Infection	1 – RR		0.70	0.66	0.74	12-16
				Mixed variant	Mixed variant	Infection	1 – RR		0.79	0.75	0.82	17-20
				Mixed variant	Mixed variant	Infection	1 – RR		0.81	0.78	0.85	21-24
				Mixed variant	Mixed variant	Infection	1 – RR		0.74	0.67	0.79	25-28
				Mixed variant	Mixed variant	Infection	1 – RR		0.70	0.59	0.78	29-32
				Mixed variant	Mixed variant	Infection	1 – RR		0.79	0.65	0.88	33-36

Cohen C[55]	prospective cohort	All ages	South Africa	Mixed variant	Mixed variant	Symptomatic	1 - aOR	Other covariates	0.6	0.1	0.8	NA
Sun K[56]	prospective cohort	All ages	South Africa	Mixed variant	Mixed variant	Infection	1-aRR	Other covariates	0.92	0.84	0.96	12-24
				Mixed variant	Mixed variant	Infection	1-aRR		0.87	0.78	0.92	25-36
Powell A[57]	test-negative case-control	12-17	UK	Ancestral	Delta	Symptomatic	1-aOR	Age, sex, and other covariates	0.876	0.868	0.884	NA
				Alpha	Delta	Symptomatic	1-aOR		0.861	0.854	0.868	NA
				Delta	Delta	Symptomatic	1-aOR		0.923	0.917	0.929	NA
				Ancestral	Omicron (unspecified sub-lineage)	Symptomatic	1-aOR		0.327	0.277	0.374	NA
				Alpha	Omicron (unspecified sub-lineage)	Symptomatic	1-aOR		0.366	0.329	0.401	NA
				Delta	Omicron (unspecified sub-lineage)	Symptomatic	1-aOR		0.524	0.509	0.538	NA
				Omicron (unspecified sub-lineage)	Omicron (unspecified sub-lineage)	Symptomatic	1-aOR		0.593	0.467	0.69	NA
Murugesan M[58]	retrospective cohort	>18	India	mixed variant	mixed variant	Symptomatic	1-HR	Age, sex, and other covariates	0.86	0.767	0.916	NA
Dhumal S[59]	retrospective cohort	34*	India	mixed variant	mixed variant	infection	1-aHR	Age, sex, and other covariates	0.72	0.08	0.91	NA

Corrao G[60]	test-negative case-control	>18	Italy	mixed variant	mixed variant	Symptomatic	1-aOR	Other covariates	0.91	0.85	0.95	NA
Smolenov I[61]	clinical trial	>18	US	mixed variant	mixed variant	infection	1-IRR	No adjustment	0.832	0.78	0.873	NA
				mixed variant	Alpha	infection	1-IRR		0.99	0.804	0.99	NA
				mixed variant	Beta	infection	1-IRR		0.722	0.331	0.899	NA
				mixed variant	Delta	infection	1-IRR		0.72	0.613	0.872	NA
Reynolds S[62]	retrospective cohort	All ages	US	Ancestral	Ancestral	Infection	1-aHR	Age, sex, and other covariates	0.87	0.87	0.87	NA
				Ancestral	Ancestral	Infection	1-aHR		0.88	0.87	0.88	24-32
				Ancestral	Ancestral	Infection	1-aHR		0.83	0.82	0.85	33-40
				Ancestral	Ancestral	Infection	1-aHR		0.85	0.82	0.87	41-48
Narrainen F[63]	retrospective cohort	>18	UK	Ancestral	Ancestral	Infection	1-OR	No adjustment	0.973	0.805	0.996	NA
Kubale J[64]	prospective cohort	0-4	Nicaragua	mixed variant	mixed variant	Symptomatic	1-RR	No adjustment	0.61	0.2	0.8	NA
		5-9		mixed variant	mixed variant	Symptomatic	1-RR		0.64	0.2	0.7	Na
		10-14		mixed variant	mixed variant	Symptomatic	1-RR		0.49	0.3	0.9	NA
Rothberg M[65]	retrospective cohort	All ages	US	Ancestral	Omicron (unspecified sub-lineage)	infection	1-aRR	Age, sex, and other covariates	0.18	0.13	0.227	NA
				Alpha	Omicron (unspecified sub-lineage)	infection	1-aRR		0.381	0.252	0.489	NA
				Delta	Omicron (unspecif	infection	1-aRR		0.692	0.634	0.741	NA

					ied sub-lineage)							
Andeweg S[66]	retrospective cohort	>11	Netherlands	mixed variant	Delta	infection	1-aOR	Age, sex, and other covariates	0.79	0.77	0.81	NA
				mixed variant	Delta	infection	1-aOR		0.7	0.5	0.83	4-8
				mixed variant	Delta	infection	1-aOR		0.95	0.85	0.99	9-12
				mixed variant	Delta	infection	1-aOR		0.93	0.84	0.97	13-17
				mixed variant	Delta	infection	1-aOR		0.92	0.86	0.95	18-21
				mixed variant	Delta	infection	1-aOR		0.86	0.76	0.91	22-25
				mixed variant	Delta	infection	1-aOR		0.84	0.74	0.9	26-29
				mixed variant	Delta	infection	1-aOR		0.76	0.73	0.79	30-34
				mixed variant	Omicron BA.1	infection	1-aOR		0.46	0.44	0.48	NA
				mixed variant	Omicron BA.1	infection	1-aOR		0.73	0.66	0.79	4-8
				mixed variant	Omicron BA.1	infection	1-aOR		0.63	0.58	0.67	9-12
				mixed variant	Omicron BA.1	infection	1-aOR		0.57	0.52	0.62	13-17
				mixed variant	Omicron BA.1	infection	1-aOR		0.55	0.46	0.62	18-21
				mixed variant	Omicron BA.1	infection	1-aOR		0.53	0.43	0.6	22-25
				mixed variant	Omicron BA.1	infection	1-aOR		0.52	0.45	0.58	26-29
				mixed variant	Omicron BA.1	infection	1-aOR		0.34	0.31	0.38	30-34
				mixed variant	Omicron BA.2	infection	1-aOR		0.47	0.44	0.5	NA

				mixed variant	Omicron BA.2	infection	1-aOR		0.76	0.68	0.82	4-8
				mixed variant	Omicron BA.2	infection	1-aOR		0.56	0.48	0.62	9-12
				mixed variant	Omicron BA.2	infection	1-aOR		0.5	0.43	0.56	13-17
				mixed variant	Omicron BA.2	infection	1-aOR		0.57	0.49	0.64	18-21
				mixed variant	Omicron BA.2	infection	1-aOR		0.5	0.36	0.61	22-25
				mixed variant	Omicron BA.2	infection	1-aOR		0.53	0.42	0.62	26-29
				mixed variant	Omicron BA.2	infection	1-aOR		0.38	0.34	0.43	30-34
				mixed variant	Delta	infection	1-aOR		0.8	0.78	0.82	NA
				mixed variant	Delta	infection	1-aOR		0.7	0.48	0.83	4-8
				mixed variant	Delta	infection	1-aOR		0.98	0.86	0.99	9-12
				mixed variant	Delta	infection	1-aOR		0.94	0.83	0.98	13-17
				mixed variant	Delta	infection	1-aOR		0.92	0.86	0.95	18-21
				mixed variant	Delta	infection	1-aOR		0.84	0.73	0.9	22-25
				mixed variant	Delta	infection	1-aOR		0.88	0.78	0.93	26-29
				mixed variant	Delta	infection	1-aOR		0.77	0.74	0.8	30-34
				mixed variant	Omicron BA.1	infection	1-aOR		0.47	0.44	0.49	NA
				mixed variant	Omicron BA.1	infection	1-aOR		0.73	0.66	0.8	4-8
				mixed variant	Omicron BA.1	infection	1-aOR		0.62	0.57	0.67	9-12

				mixed variant	Omicron BA.1	infection	1-aOR		0.55	0.48	0.6	13-17
				mixed variant	Omicron BA.1	infection	1-aOR		0.51	0.41	0.6	18-21
				mixed variant	Omicron BA.1	infection	1-aOR		0.54	0.44	0.62	22-25
				mixed variant	Omicron BA.1	infection	1-aOR		0.53	0.45	0.59	26-29
				mixed variant	Omicron BA.1	infection	1-aOR		0.37	0.33	0.4	30-34
				mixed variant	Omicron BA.2	infection	1-aOR		0.49	0.45	0.52	NA
				mixed variant	Omicron BA.2	infection	1-aOR		0.76	0.69	0.82	4-8
				mixed variant	Omicron BA.2	infection	1-aOR		0.57	0.49	0.63	9-12
				mixed variant	Omicron BA.2	infection	1-aOR		0.5	0.43	0.56	13-17
				mixed variant	Omicron BA.2	infection	1-aOR		0.58	0.5	0.66	18-21
				mixed variant	Omicron BA.2	infection	1-aOR		0.55	0.41	0.65	22-25
				mixed variant	Omicron BA.2	infection	1-aOR		0.52	0.4	0.62	26-29
				mixed variant	Omicron BA.2	infection	1-aOR		0.4	0.35	0.44	30-34

CI = Confidence Interval; NA = not available; * indicates extracted data point is median age

Table S2 Protection against reinfection, symptomatic, and severe disease by time since infection

	Protection against ancestral, Alpha, and Delta reinfection			Protection against Omicron BA.1 reinfection			Protection against Omicron BA.2 reinfection			Protection against ancestral, Alpha, and Delta symptomatic disease			Protection against BA.1 symptomatic disease			Protection against ancestral, Alpha, and Delta severe disease			Protection against BA.1 severe disease		
Week after infection	Estimate	Lower bound	Upper bound	Estimate	Lower bound	Upper bound	Estimate	Lower bound	Upper bound	Estimate	Lower bound	Upper bound	Estimate	Lower bound	Upper bound	Estimate	Lower bound	Upper bound	Estimate	Lower bound	Upper bound
0	85.2%	61.0%	96.0%	77.5%	66.1%	86.1%	96.9%	83.6%	99.0%	90.3%	80.4%	98.4%	89.2%	67.1%	95.8%	96.7%	90.9%	100.0%	88.9%	86.9%	99.9%
0.1	85.2%	61.0%	96.0%	77.4%	66.0%	86.1%	96.8%	83.4%	98.9%	90.3%	80.4%	98.4%	89.0%	67.0%	95.7%	96.7%	90.9%	100.0%	88.9%	86.9%	99.9%
0.2	85.2%	61.0%	96.0%	77.4%	65.9%	86.0%	96.6%	83.3%	98.9%	90.3%	80.4%	98.3%	88.8%	67.0%	95.6%	96.7%	91.1%	100.0%	88.9%	86.9%	99.9%
0.3	85.2%	61.0%	96.0%	77.3%	65.8%	86.0%	96.5%	83.1%	98.8%	90.3%	80.4%	98.3%	88.6%	66.9%	95.5%	96.7%	91.1%	100.0%	88.9%	86.9%	99.9%
0.4	85.2%	61.0%	96.0%	77.2%	65.7%	85.9%	96.3%	82.8%	98.7%	90.3%	80.4%	98.2%	88.4%	66.7%	95.4%	96.8%	91.1%	100.0%	88.9%	86.9%	99.9%
0.5	85.2%	61.0%	96.0%	77.1%	65.6%	85.9%	96.1%	82.6%	98.6%	90.3%	80.3%	98.2%	88.2%	66.3%	95.2%	96.8%	91.1%	100.0%	88.9%	86.9%	99.9%
0.6	85.2%	61.0%	96.0%	77.0%	65.5%	85.8%	96.0%	82.3%	98.5%	90.3%	80.3%	98.2%	88.0%	65.9%	95.1%	96.8%	91.2%	100.0%	88.9%	86.9%	99.9%
0.7	85.2%	61.0%	96.0%	76.9%	65.4%	85.8%	95.8%	82.1%	98.4%	90.3%	80.3%	98.1%	87.7%	65.9%	94.9%	96.9%	91.2%	100.0%	88.9%	86.9%	99.9%
0.8	85.2%	61.0%	96.0%	76.8%	65.2%	85.7%	95.6%	81.8%	98.3%	90.3%	80.2%	98.1%	87.5%	65.8%	94.7%	96.9%	91.2%	100.0%	88.9%	86.9%	99.9%
0.9	85.2%	60.9%	96.0%	76.8%	65.1%	85.6%	95.4%	81.6%	98.2%	90.3%	80.2%	98.1%	87.3%	65.6%	94.5%	96.9%	91.2%	100.0%	88.9%	86.9%	99.8%
1	85.2%	60.9%	96.0%	76.7%	65.0%	85.6%	95.2%	81.3%	98.1%	90.3%	80.1%	98.0%	87.1%	65.5%	94.4%	96.9%	91.2%	100.0%	88.9%	87.0%	99.8%
1.1	85.2%	60.9%	96.0%	76.6%	64.9%	85.5%	94.9%	81.0%	98.0%	90.3%	80.1%	98.0%	86.9%	65.4%	94.3%	97.0%	91.2%	100.0%	88.9%	87.0%	99.8%
1.2	85.2%	60.9%	96.0%	76.5%	64.8%	85.5%	94.7%	80.8%	97.9%	90.3%	80.1%	97.9%	86.6%	65.3%	94.3%	97.0%	91.2%	100.0%	88.9%	87.0%	99.8%
1.3	85.2%	60.9%	96.0%	76.4%	64.7%	85.4%	94.5%	80.5%	97.7%	90.3%	80.0%	97.9%	86.4%	65.2%	94.1%	97.0%	91.3%	100.0%	88.9%	87.0%	99.8%
1.4	85.2%	60.8%	96.0%	76.3%	64.5%	85.4%	94.3%	80.3%	97.6%	90.3%	80.0%	97.9%	86.2%	65.2%	93.9%	97.0%	91.3%	100.0%	88.9%	87.0%	99.8%
1.5	85.2%	60.8%	96.0%	76.3%	64.4%	85.3%	94.0%	80.0%	97.5%	90.3%	80.0%	97.8%	85.9%	65.1%	93.7%	97.1%	91.3%	100.0%	88.9%	87.0%	99.8%
1.6	85.2%	60.8%	96.0%	76.2%	64.3%	85.3%	93.8%	79.7%	97.3%	90.3%	80.0%	97.8%	85.7%	65.0%	93.6%	97.1%	91.3%	100.0%	88.9%	87.0%	99.8%
1.7	85.2%	60.8%	96.0%	76.1%	64.2%	85.2%	93.5%	79.5%	97.2%	90.3%	80.0%	97.7%	85.5%	65.0%	93.4%	97.1%	91.3%	100.0%	88.9%	87.0%	99.8%
1.8	85.2%	60.8%	96.0%	76.0%	64.1%	85.2%	93.2%	79.2%	97.0%	90.3%	80.0%	97.7%	85.2%	64.9%	93.3%	97.1%	91.3%	100.0%	88.9%	87.0%	99.8%
1.9	85.2%	60.8%	96.0%	75.9%	64.0%	85.1%	93.0%	78.9%	96.8%	90.3%	80.0%	97.7%	85.0%	64.8%	93.1%	97.2%	91.4%	100.0%	88.9%	87.0%	99.8%
2	85.2%	60.8%	96.0%	75.8%	63.8%	85.0%	92.7%	78.7%	96.6%	90.3%	80.0%	97.7%	84.7%	64.7%	93.0%	97.2%	91.4%	100.0%	88.9%	87.0%	99.8%
2.1	85.2%	60.8%	96.0%	75.7%	63.7%	85.0%	92.4%	78.4%	96.4%	90.3%	80.0%	97.7%	84.5%	64.7%	92.9%	97.2%	91.5%	100.0%	88.9%	87.0%	99.8%
2.2	85.2%	60.8%	96.0%	75.6%	63.6%	84.9%	92.1%	78.2%	96.2%	90.3%	80.0%	97.7%	84.2%	64.6%	92.7%	97.2%	91.5%	100.0%	88.9%	87.0%	99.7%
2.3	85.2%	60.8%	96.0%	75.6%	63.5%	84.9%	91.8%	78.0%	96.0%	90.3%	80.0%	97.7%	84.0%	64.2%	92.5%	97.2%	91.5%	100.0%	88.9%	87.0%	99.7%
2.4	85.2%	60.8%	96.0%	75.5%	63.4%	84.8%	91.5%	77.8%	95.8%	90.3%	80.0%	97.7%	83.8%	63.9%	92.3%	97.3%	91.6%	100.0%	88.9%	87.0%	99.7%
2.5	85.2%	60.8%	96.0%	75.4%	63.3%	84.8%	91.1%	77.5%	95.6%	90.3%	80.0%	97.6%	83.5%	63.5%	92.1%	97.3%	91.6%	100.0%	88.9%	87.0%	99.7%

2.6	85.2%	60.8%	96.0%	75.3%	63.1%	84.7%	90.8%	77.2%	95.4%	90.3%	80.0%	97.6%	83.3%	63.2%	91.9%	97.3%	91.6%	100.0%	88.9%	87.0%	99.7%
2.7	85.2%	60.8%	96.0%	75.2%	63.0%	84.6%	90.5%	77.0%	95.1%	90.3%	80.0%	97.5%	83.0%	63.1%	91.7%	97.3%	91.7%	100.0%	88.9%	87.0%	99.7%
2.8	85.2%	60.8%	96.0%	75.1%	62.9%	84.6%	90.1%	76.8%	94.9%	90.3%	80.0%	97.5%	82.8%	63.0%	91.6%	97.3%	91.7%	100.0%	88.9%	87.0%	99.7%
2.9	85.2%	60.8%	96.0%	75.0%	62.8%	84.5%	89.8%	76.7%	94.6%	90.3%	80.0%	97.5%	82.5%	62.8%	91.5%	97.3%	91.7%	100.0%	88.9%	87.0%	99.7%
3	85.2%	60.8%	96.0%	74.9%	62.7%	84.5%	89.4%	76.4%	94.3%	90.3%	80.0%	97.4%	82.3%	62.7%	91.3%	97.4%	91.8%	100.0%	88.9%	87.0%	99.6%
3.1	85.2%	60.8%	96.0%	74.8%	62.5%	84.4%	89.0%	76.1%	94.0%	90.3%	80.0%	97.4%	82.0%	62.5%	91.2%	97.4%	91.8%	100.0%	88.9%	87.0%	99.6%
3.2	85.2%	60.8%	96.0%	74.8%	62.4%	84.4%	88.7%	75.9%	93.8%	90.3%	80.0%	97.4%	81.7%	62.5%	91.0%	97.4%	91.8%	100.0%	88.9%	87.0%	99.6%
3.3	85.2%	60.8%	96.0%	74.7%	62.3%	84.3%	88.3%	75.7%	93.5%	90.3%	80.0%	97.4%	81.5%	62.4%	90.8%	97.4%	91.9%	100.0%	88.9%	87.0%	99.6%
3.4	85.2%	60.8%	96.0%	74.6%	62.2%	84.2%	87.9%	75.5%	93.1%	90.3%	80.0%	97.4%	81.2%	62.3%	90.7%	97.4%	91.9%	100.0%	88.9%	87.0%	99.6%
3.5	85.2%	60.8%	96.0%	74.5%	62.1%	84.2%	87.5%	75.3%	92.8%	90.3%	80.0%	97.4%	81.0%	62.1%	90.5%	97.4%	91.9%	100.0%	88.9%	87.0%	99.6%
3.6	85.2%	60.8%	96.0%	74.4%	61.9%	84.1%	87.1%	74.9%	92.5%	90.3%	80.0%	97.3%	80.7%	61.9%	90.3%	97.4%	92.0%	100.0%	88.9%	87.0%	99.5%
3.7	85.2%	60.8%	96.0%	74.3%	61.8%	84.1%	86.7%	74.7%	92.2%	90.3%	80.0%	97.3%	80.5%	61.7%	90.2%	97.5%	92.0%	100.0%	88.9%	87.0%	99.5%
3.8	85.2%	60.8%	96.0%	74.2%	61.7%	84.0%	86.2%	74.6%	91.8%	90.3%	80.0%	97.3%	80.2%	61.5%	90.0%	97.5%	92.0%	100.0%	88.9%	87.0%	99.5%
3.9	85.2%	60.8%	96.0%	74.1%	61.6%	84.0%	85.8%	74.2%	91.5%	90.3%	80.0%	97.3%	79.9%	61.3%	89.8%	97.5%	92.0%	100.0%	88.9%	87.0%	99.5%
4	85.2%	60.8%	96.0%	74.0%	61.5%	83.9%	85.4%	74.0%	91.1%	90.3%	80.0%	97.2%	79.7%	61.0%	89.7%	97.5%	92.0%	100.0%	88.9%	87.0%	99.5%
4.1	85.2%	60.8%	96.0%	73.9%	61.3%	83.8%	85.0%	73.7%	90.7%	90.3%	80.0%	97.2%	79.4%	60.7%	89.5%	97.5%	92.0%	100.0%	88.9%	87.0%	99.5%
4.2	85.2%	60.8%	96.0%	73.8%	61.2%	83.8%	84.5%	73.4%	90.3%	90.3%	80.0%	97.1%	79.2%	60.5%	89.3%	97.5%	92.1%	100.0%	88.9%	87.0%	99.4%
4.3	85.2%	60.8%	96.0%	73.8%	61.1%	83.7%	84.1%	73.2%	89.9%	90.3%	80.0%	97.1%	78.9%	60.2%	89.1%	97.5%	92.1%	99.9%	88.9%	87.0%	99.4%
4.4	85.2%	60.8%	95.9%	73.7%	61.0%	83.6%	83.6%	72.9%	89.5%	90.3%	80.0%	97.1%	78.6%	60.0%	88.9%	97.5%	92.1%	99.9%	88.9%	87.0%	99.4%
4.5	85.2%	60.8%	95.9%	73.6%	60.9%	83.5%	83.1%	72.6%	89.0%	90.3%	80.0%	97.1%	78.4%	59.7%	88.7%	97.6%	92.2%	99.9%	88.9%	87.0%	99.4%
4.6	85.2%	60.8%	95.9%	73.5%	60.8%	83.5%	82.7%	72.3%	88.6%	90.3%	79.9%	97.1%	78.1%	59.4%	88.5%	97.6%	92.2%	99.9%	88.9%	87.0%	99.4%
4.7	85.2%	60.8%	95.9%	73.4%	60.7%	83.4%	82.2%	71.9%	88.1%	90.3%	79.8%	97.1%	77.9%	59.1%	88.2%	97.6%	92.2%	99.9%	88.9%	87.0%	99.3%
4.8	85.2%	60.8%	95.9%	73.3%	60.6%	83.3%	81.7%	71.6%	87.6%	90.3%	79.7%	97.0%	77.6%	58.9%	88.0%	97.6%	92.2%	99.9%	88.9%	87.0%	99.3%
4.9	85.2%	60.8%	95.9%	73.2%	60.5%	83.2%	81.3%	71.2%	87.2%	90.3%	79.6%	97.0%	77.3%	58.9%	87.8%	97.6%	92.1%	99.9%	88.9%	87.0%	99.3%
5	85.2%	60.8%	95.9%	73.1%	60.4%	83.2%	80.8%	70.9%	86.7%	90.3%	79.6%	96.9%	77.1%	58.7%	87.7%	97.6%	92.1%	99.9%	88.9%	87.0%	99.3%
5.1	85.2%	60.8%	95.9%	73.0%	60.3%	83.1%	80.3%	70.6%	86.2%	90.3%	79.6%	96.9%	76.8%	58.3%	87.5%	97.6%	92.2%	99.9%	88.9%	87.0%	99.2%
5.2	85.2%	60.8%	95.9%	72.9%	60.2%	83.0%	79.8%	70.3%	85.8%	90.3%	79.6%	96.9%	76.6%	58.0%	87.3%	97.6%	92.2%	99.9%	88.9%	87.0%	99.2%
5.3	85.2%	60.8%	95.9%	72.8%	60.1%	82.9%	79.3%	69.9%	85.3%	90.3%	79.6%	96.9%	76.3%	57.9%	87.0%	97.6%	92.3%	99.9%	88.9%	87.0%	99.2%
5.4	85.2%	60.8%	95.9%	72.7%	60.0%	82.9%	78.8%	69.7%	84.8%	90.3%	79.6%	96.8%	76.0%	57.8%	86.9%	97.6%	92.3%	99.9%	88.9%	87.0%	99.1%
5.5	85.2%	60.8%	95.9%	72.6%	59.9%	82.8%	78.3%	69.4%	84.3%	90.3%	79.6%	96.8%	75.8%	57.5%	86.7%	97.6%	92.3%	99.9%	88.9%	87.0%	99.1%
5.6	85.2%	60.8%	95.9%	72.5%	59.8%	82.8%	77.8%	69.1%	83.8%	90.3%	79.6%	96.8%	75.5%	57.3%	86.5%	97.6%	92.3%	99.9%	88.9%	87.0%	99.1%
5.7	85.2%	60.8%	95.9%	72.4%	59.8%	82.7%	77.3%	68.8%	83.3%	90.3%	79.5%	96.8%	75.3%	57.1%	86.3%	97.7%	92.3%	99.9%	88.9%	87.0%	99.1%

5.8	85.2%	60.8%	95.9%	72.3%	59.7%	82.7%	76.8%	68.5%	82.8%	90.3%	79.3%	96.8%	75.0%	57.1%	86.0%	97.7%	92.3%	99.9%	88.9%	87.0%	99.0%
5.9	85.2%	60.8%	95.9%	72.3%	59.6%	82.6%	76.3%	68.1%	82.3%	90.3%	79.2%	96.8%	74.8%	56.7%	85.8%	97.7%	92.3%	99.9%	88.9%	87.0%	99.0%
6	85.2%	60.8%	95.9%	72.2%	59.5%	82.5%	75.8%	67.8%	81.8%	90.3%	79.1%	96.7%	74.5%	56.5%	85.7%	97.7%	92.3%	99.9%	88.9%	87.0%	99.0%
6.1	85.2%	60.8%	95.9%	72.1%	59.4%	82.5%	75.3%	67.5%	81.3%	90.3%	78.9%	96.7%	74.3%	56.2%	85.6%	97.7%	92.3%	99.9%	88.9%	87.0%	99.0%
6.2	85.2%	60.8%	95.9%	72.0%	59.3%	82.4%	74.8%	67.2%	80.7%	90.3%	78.8%	96.7%	74.0%	56.1%	85.4%	97.7%	92.3%	99.9%	88.9%	87.0%	98.9%
6.3	85.2%	60.8%	95.9%	71.9%	59.2%	82.4%	74.3%	66.9%	80.1%	90.3%	78.7%	96.7%	73.8%	55.9%	85.2%	97.7%	92.3%	99.9%	88.9%	87.0%	98.9%
6.4	85.2%	60.8%	95.9%	71.8%	59.1%	82.3%	73.8%	66.6%	79.6%	90.3%	78.7%	96.7%	73.5%	55.8%	85.0%	97.7%	92.4%	99.8%	88.9%	87.0%	98.9%
6.5	85.2%	60.8%	95.9%	71.7%	59.0%	82.2%	73.3%	66.2%	79.1%	90.3%	78.7%	96.7%	73.3%	55.6%	84.8%	97.7%	92.4%	99.8%	88.9%	87.0%	98.8%
6.6	85.2%	60.8%	95.9%	71.6%	58.9%	82.1%	72.8%	65.9%	78.6%	90.3%	78.7%	96.7%	73.0%	55.5%	84.6%	97.7%	92.4%	99.8%	88.9%	87.0%	98.8%
6.7	85.2%	60.8%	95.9%	71.5%	58.7%	82.1%	72.3%	65.5%	78.0%	90.3%	78.7%	96.6%	72.8%	55.3%	84.5%	97.7%	92.4%	99.8%	88.9%	87.0%	98.8%
6.8	85.2%	60.8%	95.9%	71.4%	58.6%	82.0%	71.8%	65.3%	77.4%	90.3%	78.6%	96.6%	72.5%	55.2%	84.3%	97.7%	92.4%	99.8%	88.9%	87.0%	98.7%
6.9	85.2%	60.8%	95.9%	71.3%	58.5%	81.9%	71.3%	65.0%	76.9%	90.3%	78.6%	96.6%	72.3%	55.0%	84.3%	97.7%	92.4%	99.8%	88.9%	87.0%	98.7%
7	85.2%	60.8%	95.9%	71.2%	58.4%	81.8%	70.8%	64.6%	76.3%	90.3%	78.6%	96.6%	72.0%	54.7%	84.1%	97.7%	92.4%	99.8%	88.9%	87.0%	98.7%
7.1	85.2%	60.8%	95.9%	71.1%	58.3%	81.8%	70.3%	64.3%	75.7%	90.3%	78.6%	96.5%	71.8%	54.3%	83.9%	97.7%	92.4%	99.8%	88.9%	87.0%	98.6%
7.2	85.2%	60.8%	95.9%	71.0%	58.1%	81.7%	69.8%	63.9%	75.1%	90.3%	78.6%	96.5%	71.5%	54.1%	83.8%	97.7%	92.4%	99.8%	88.9%	87.0%	98.6%
7.3	85.2%	60.8%	95.9%	70.9%	58.0%	81.6%	69.3%	63.6%	74.6%	90.3%	78.6%	96.5%	71.3%	54.0%	83.6%	97.7%	92.5%	99.8%	88.9%	87.0%	98.5%
7.4	85.2%	60.8%	95.9%	70.8%	57.9%	81.5%	68.9%	63.3%	74.0%	90.3%	78.5%	96.5%	71.1%	53.7%	83.4%	97.7%	92.5%	99.8%	88.9%	86.9%	98.5%
7.5	85.2%	60.8%	95.9%	70.7%	57.8%	81.4%	68.4%	62.9%	73.5%	90.3%	78.5%	96.5%	70.8%	53.4%	83.2%	97.7%	92.5%	99.8%	88.9%	86.9%	98.4%
7.6	85.2%	60.8%	95.9%	70.6%	57.7%	81.4%	67.9%	62.6%	73.0%	90.3%	78.5%	96.5%	70.6%	53.2%	83.0%	97.7%	92.5%	99.8%	88.9%	86.9%	98.4%
7.7	85.2%	60.8%	95.9%	70.5%	57.5%	81.3%	67.4%	62.3%	72.6%	90.3%	78.5%	96.5%	70.4%	53.0%	82.9%	97.7%	92.5%	99.8%	88.9%	86.9%	98.3%
7.8	85.2%	60.8%	95.9%	70.4%	57.4%	81.2%	67.0%	62.0%	72.1%	90.3%	78.5%	96.4%	70.1%	52.8%	82.8%	97.7%	92.5%	99.7%	88.9%	86.9%	98.3%
7.9	85.2%	60.8%	95.9%	70.3%	57.3%	81.1%	66.5%	61.6%	71.7%	90.3%	78.5%	96.4%	69.9%	52.6%	82.7%	97.7%	92.5%	99.7%	88.9%	86.9%	98.2%
8	85.2%	60.8%	95.9%	70.2%	57.1%	81.0%	66.1%	61.3%	71.2%	90.3%	78.4%	96.4%	69.7%	52.4%	82.6%	97.7%	92.5%	99.7%	88.9%	86.9%	98.2%
8.1	85.2%	60.8%	95.9%	70.1%	57.0%	81.0%	65.6%	61.0%	70.7%	90.3%	78.4%	96.4%	69.5%	52.3%	82.5%	97.7%	92.5%	99.7%	88.9%	86.9%	98.1%
8.2	85.2%	60.8%	95.9%	70.0%	56.9%	80.9%	65.2%	60.7%	70.3%	90.3%	78.4%	96.4%	69.2%	52.1%	82.2%	97.7%	92.4%	99.7%	88.9%	86.9%	98.1%
8.3	85.2%	60.8%	95.9%	69.9%	56.7%	80.8%	64.7%	60.4%	69.9%	90.3%	78.4%	96.4%	69.0%	51.9%	82.0%	97.7%	92.4%	99.7%	88.9%	86.9%	98.0%
8.4	85.2%	60.8%	95.9%	69.8%	56.6%	80.7%	64.3%	60.1%	69.5%	90.3%	78.4%	96.4%	68.8%	51.7%	81.8%	97.7%	92.4%	99.7%	88.9%	86.9%	98.0%
8.5	85.2%	60.8%	95.9%	69.7%	56.5%	80.7%	63.9%	59.7%	69.1%	90.3%	78.4%	96.4%	68.6%	51.6%	81.6%	97.7%	92.4%	99.7%	88.9%	86.9%	97.9%
8.6	85.2%	60.8%	95.9%	69.6%	56.3%	80.6%	63.5%	59.5%	68.7%	90.3%	78.4%	96.4%	68.4%	51.4%	81.4%	97.7%	92.4%	99.6%	88.9%	86.9%	97.9%
8.7	85.2%	60.8%	95.9%	69.5%	56.2%	80.5%	63.1%	59.2%	68.3%	90.3%	78.3%	96.4%	68.1%	51.2%	81.1%	97.7%	92.4%	99.6%	88.9%	86.9%	97.8%
8.8	85.2%	60.8%	95.9%	69.4%	56.1%	80.5%	62.6%	58.9%	68.0%	90.3%	78.3%	96.4%	67.9%	51.1%	81.0%	97.7%	92.3%	99.6%	88.9%	86.9%	97.8%
8.9	85.2%	60.8%	95.9%	69.3%	55.9%	80.4%	62.3%	58.7%	67.6%	90.3%	78.3%	96.3%	67.7%	50.9%	81.0%	97.7%	92.3%	99.6%	88.9%	86.9%	97.7%

9	85.2%	60.8%	95.9%	69.2%	55.8%	80.3%	61.9%	58.4%	67.2%	90.3%	78.3%	96.3%	67.5%	50.6%	80.9%	97.7%	92.2%	99.6%	88.9%	86.9%	97.7%
9.1	85.2%	60.8%	95.9%	69.1%	55.7%	80.2%	61.5%	58.1%	66.8%	90.3%	78.3%	96.3%	67.3%	50.4%	80.8%	97.7%	92.2%	99.6%	88.9%	86.9%	97.6%
9.2	85.2%	60.8%	95.9%	69.0%	55.5%	80.2%	61.1%	57.7%	66.5%	90.3%	78.3%	96.3%	67.1%	50.1%	80.6%	97.7%	92.1%	99.6%	88.9%	86.9%	97.6%
9.3	85.2%	60.8%	95.9%	68.9%	55.4%	80.1%	60.7%	57.4%	66.2%	90.3%	78.3%	96.3%	66.9%	49.9%	80.4%	97.7%	92.1%	99.6%	88.9%	86.9%	97.5%
9.4	85.2%	60.8%	95.8%	68.8%	55.3%	80.0%	60.4%	57.1%	65.9%	90.3%	78.3%	96.3%	66.7%	49.8%	80.3%	97.7%	92.1%	99.6%	88.9%	86.9%	97.4%
9.5	85.2%	60.8%	95.8%	68.7%	55.1%	79.9%	60.0%	56.8%	65.7%	90.3%	78.2%	96.3%	66.5%	49.8%	80.3%	97.7%	92.1%	99.6%	88.9%	86.9%	97.4%
9.6	85.2%	60.8%	95.8%	68.6%	55.0%	79.9%	59.7%	56.6%	65.4%	90.3%	78.2%	96.3%	66.3%	49.6%	80.2%	97.7%	92.1%	99.6%	88.9%	86.9%	97.3%
9.7	85.2%	60.8%	95.8%	68.5%	54.9%	79.8%	59.3%	56.3%	65.1%	90.3%	78.2%	96.3%	66.1%	49.4%	80.1%	97.7%	92.1%	99.5%	88.9%	86.9%	97.3%
9.8	85.2%	60.8%	95.8%	68.4%	54.7%	79.7%	59.0%	56.0%	64.8%	90.3%	78.2%	96.3%	66.0%	49.2%	79.9%	97.7%	92.0%	99.5%	88.9%	86.9%	97.2%
9.9	85.2%	60.7%	95.8%	68.3%	54.6%	79.6%	58.7%	55.7%	64.6%	90.3%	78.2%	96.3%	65.8%	49.0%	79.8%	97.7%	91.9%	99.5%	88.9%	86.9%	97.2%
10	85.2%	60.7%	95.8%	68.2%	54.5%	79.6%	58.4%	55.5%	64.4%	90.3%	78.2%	96.3%	65.6%	48.8%	79.7%	97.7%	91.9%	99.5%	88.9%	86.9%	97.1%
10.1	85.2%	60.7%	95.8%	68.1%	54.3%	79.5%	58.1%	55.1%	64.1%	90.3%	78.2%	96.3%	65.4%	48.6%	79.6%	97.7%	91.8%	99.5%	88.9%	86.9%	97.0%
10.2	85.2%	60.7%	95.8%	68.0%	54.2%	79.4%	57.8%	54.8%	63.9%	90.3%	78.2%	96.3%	65.2%	48.4%	79.4%	97.7%	91.8%	99.5%	88.9%	86.9%	97.0%
10.3	85.2%	60.7%	95.8%	67.9%	54.1%	79.4%	57.5%	54.6%	63.7%	90.3%	78.2%	96.3%	65.1%	48.2%	79.3%	97.7%	91.8%	99.5%	88.9%	86.8%	96.9%
10.4	85.2%	60.7%	95.8%	67.8%	53.9%	79.3%	57.2%	54.3%	63.5%	90.3%	78.1%	96.3%	64.9%	48.0%	79.1%	97.6%	91.7%	99.5%	88.9%	86.8%	96.8%
10.5	85.2%	60.7%	95.8%	67.7%	53.8%	79.2%	56.9%	54.0%	63.2%	90.3%	78.1%	96.3%	64.7%	47.8%	79.0%	97.6%	91.7%	99.5%	88.9%	86.8%	96.8%
10.6	85.2%	60.7%	95.8%	67.6%	53.7%	79.1%	56.7%	53.7%	63.0%	90.3%	78.1%	96.3%	64.5%	47.7%	78.9%	97.6%	91.7%	99.5%	88.9%	86.8%	96.7%
10.7	85.2%	60.7%	95.8%	67.5%	53.5%	79.1%	56.4%	53.4%	62.8%	90.3%	78.1%	96.2%	64.4%	47.6%	78.8%	97.6%	91.7%	99.5%	88.9%	86.8%	96.6%
10.8	85.2%	60.7%	95.8%	67.3%	53.4%	79.0%	56.2%	53.2%	62.6%	90.3%	78.1%	96.2%	64.2%	47.3%	78.7%	97.6%	91.6%	99.5%	88.9%	86.8%	96.6%
10.9	85.2%	60.7%	95.8%	67.2%	53.3%	78.9%	55.9%	52.9%	62.3%	90.3%	78.1%	96.2%	64.1%	47.2%	78.6%	97.6%	91.5%	99.5%	88.9%	86.8%	96.5%
11	85.2%	60.7%	95.8%	67.1%	53.1%	78.8%	55.7%	52.7%	62.1%	90.3%	78.1%	96.2%	63.9%	47.0%	78.5%	97.6%	91.4%	99.5%	88.9%	86.8%	96.4%
11.1	85.2%	60.7%	95.8%	67.0%	53.0%	78.8%	55.5%	52.5%	61.9%	90.3%	78.1%	96.2%	63.8%	46.9%	78.4%	97.6%	91.3%	99.5%	88.9%	86.8%	96.4%
11.2	85.2%	60.7%	95.8%	66.9%	52.9%	78.7%	55.3%	52.3%	61.6%	90.3%	78.1%	96.1%	63.6%	46.8%	78.3%	97.6%	91.2%	99.5%	88.9%	86.8%	96.4%
11.3	85.2%	60.7%	95.8%	66.8%	52.7%	78.6%	55.1%	52.0%	61.5%	90.3%	78.1%	96.1%	63.5%	46.6%	78.2%	97.6%	91.1%	99.5%	88.9%	86.8%	96.3%
11.4	85.2%	60.7%	95.8%	66.7%	52.6%	78.5%	54.9%	51.7%	61.3%	90.3%	78.1%	96.0%	63.3%	46.5%	78.1%	97.6%	91.1%	99.5%	88.9%	86.8%	96.2%
11.5	85.2%	60.7%	95.8%	66.6%	52.5%	78.4%	54.7%	51.5%	61.1%	90.3%	78.1%	96.0%	63.2%	46.4%	78.1%	97.6%	91.0%	99.4%	88.9%	86.8%	96.1%
11.6	85.2%	60.7%	95.8%	66.5%	52.4%	78.4%	54.6%	51.4%	60.9%	90.3%	78.0%	96.0%	63.0%	46.2%	78.0%	97.5%	91.0%	99.4%	88.9%	86.8%	96.1%
11.7	85.2%	60.7%	95.8%	66.4%	52.3%	78.3%	54.4%	51.2%	60.7%	90.3%	78.0%	96.0%	62.9%	46.1%	77.9%	97.5%	90.9%	99.4%	88.9%	86.8%	96.0%
11.8	85.2%	60.7%	95.8%	66.3%	52.2%	78.2%	54.2%	51.1%	60.5%	90.3%	78.0%	96.0%	62.8%	46.0%	77.8%	97.5%	90.9%	99.4%	88.9%	86.8%	95.9%
11.9	85.2%	60.7%	95.8%	66.2%	52.1%	78.1%	54.1%	50.9%	60.4%	90.3%	78.0%	96.0%	62.6%	45.7%	77.8%	97.5%	90.8%	99.4%	88.9%	86.8%	95.8%
12	85.2%	60.7%	95.8%	66.1%	52.0%	78.1%	54.0%	50.7%	60.2%	90.3%	78.0%	96.0%	62.5%	45.5%	77.7%	97.5%	90.7%	99.4%	88.9%	86.8%	95.8%
12.1	85.2%	60.7%	95.8%	66.0%	51.9%	78.0%	53.8%	50.6%	60.1%	90.3%	78.0%	96.0%	62.4%	45.4%	77.6%	97.5%	90.7%	99.4%	88.9%	86.8%	95.7%

12.2	85.2%	60.7%	95.8%	65.9%	51.8%	77.9%	53.7%	50.5%	59.9%	90.3%	78.0%	96.0%	62.3%	45.3%	77.5%	97.5%	90.6%	99.4%	88.9%	86.8%	95.6%
12.3	85.2%	60.7%	95.8%	65.8%	51.7%	77.8%	53.6%	50.3%	59.6%	90.3%	78.0%	96.0%	62.1%	45.2%	77.5%	97.5%	90.5%	99.4%	88.9%	86.8%	95.5%
12.4	85.2%	60.7%	95.8%	65.7%	51.6%	77.7%	53.5%	50.2%	59.5%	90.3%	78.0%	96.0%	62.0%	45.1%	77.4%	97.4%	90.5%	99.4%	88.9%	86.8%	95.4%
12.5	85.2%	60.7%	95.8%	65.5%	51.5%	77.7%	53.4%	50.1%	59.3%	90.3%	78.0%	95.9%	61.9%	45.0%	77.3%	97.4%	90.4%	99.3%	88.9%	86.8%	95.3%
12.6	85.2%	60.7%	95.8%	65.4%	51.4%	77.6%	53.3%	50.0%	59.2%	90.3%	78.0%	95.9%	61.8%	44.9%	77.3%	97.4%	90.3%	99.3%	88.9%	86.8%	95.3%
12.7	85.2%	60.7%	95.8%	65.3%	51.3%	77.5%	53.3%	49.9%	59.1%	90.3%	78.0%	95.9%	61.7%	44.8%	77.2%	97.4%	90.3%	99.3%	88.9%	86.8%	95.2%
12.8	85.2%	60.7%	95.8%	65.2%	51.1%	77.4%	53.2%	49.8%	59.0%	90.3%	78.0%	95.9%	61.6%	44.7%	77.2%	97.4%	90.3%	99.3%	88.9%	86.8%	95.1%
12.9	85.2%	60.7%	95.8%	65.1%	51.0%	77.3%	53.1%	49.8%	58.8%	90.3%	77.9%	95.9%	61.5%	44.5%	77.1%	97.4%	90.3%	99.3%	88.9%	86.8%	95.0%
13	85.2%	60.7%	95.8%	65.0%	50.9%	77.3%	53.1%	49.7%	58.6%	90.3%	77.8%	95.9%	61.4%	44.4%	77.0%	97.4%	90.2%	99.3%	88.9%	86.8%	95.0%
13.1	85.2%	60.7%	95.8%	64.9%	50.7%	77.2%	53.1%	49.7%	58.5%	90.3%	77.8%	95.9%	61.3%	44.3%	76.9%	97.3%	90.2%	99.3%	88.9%	86.8%	94.9%
13.2	85.2%	60.7%	95.8%	64.8%	50.6%	77.1%	53.0%	49.6%	58.4%	90.3%	77.7%	95.9%	61.2%	44.1%	76.8%	97.3%	90.1%	99.3%	88.9%	86.8%	94.8%
13.3	85.2%	60.7%	95.8%	64.7%	50.5%	77.0%	53.0%	49.6%	58.3%	90.3%	77.6%	95.9%	61.1%	44.0%	76.7%	97.3%	90.1%	99.3%	88.9%	86.8%	94.7%
13.4	85.2%	60.7%	95.8%	64.6%	50.3%	76.9%	53.0%	49.6%	58.2%	90.3%	77.6%	95.9%	61.0%	43.9%	76.5%	97.3%	90.0%	99.3%	88.9%	86.8%	94.6%
13.5	85.2%	60.7%	95.8%	64.5%	50.2%	76.9%	53.0%	49.6%	58.1%	90.3%	77.5%	95.9%	61.0%	43.8%	76.4%	97.3%	89.9%	99.3%	88.9%	86.8%	94.6%
13.6	85.2%	60.7%	95.8%	64.4%	50.1%	76.8%	53.0%	49.6%	58.0%	90.3%	77.5%	95.9%	60.9%	43.7%	76.3%	97.3%	89.8%	99.3%	88.9%	86.8%	94.5%
13.7	85.2%	60.7%	95.8%	64.2%	50.0%	76.7%	53.0%	49.6%	58.0%	90.3%	77.5%	95.9%	60.8%	43.6%	76.2%	97.2%	89.7%	99.3%	88.9%	86.8%	94.4%
13.8	85.2%	60.7%	95.8%	64.1%	49.9%	76.6%	53.0%	49.6%	57.9%	90.3%	77.5%	95.9%	60.7%	43.5%	76.1%	97.2%	89.6%	99.3%	88.9%	86.8%	94.3%
13.9	85.2%	60.7%	95.8%	64.0%	49.8%	76.5%	53.0%	49.6%	57.9%	90.3%	77.5%	95.9%	60.7%	43.4%	76.0%	97.2%	89.5%	99.3%	88.9%	86.8%	94.3%
14	85.2%	60.7%	95.8%	63.9%	49.6%	76.5%	53.0%	49.6%	57.9%	90.3%	77.5%	95.9%	60.6%	43.3%	75.9%	97.2%	89.4%	99.3%	88.9%	86.8%	94.2%
14.1	85.2%	60.7%	95.8%	63.8%	49.5%	76.4%	53.0%	49.5%	57.9%	90.3%	77.5%	95.9%	60.5%	43.3%	75.8%	97.2%	89.3%	99.3%	88.9%	86.8%	94.1%
14.2	85.2%	60.7%	95.8%	63.7%	49.4%	76.3%	53.0%	49.5%	57.9%	90.3%	77.5%	95.9%	60.5%	43.2%	75.7%	97.1%	89.2%	99.3%	88.9%	86.8%	94.1%
14.3	85.2%	60.7%	95.8%	63.6%	49.2%	76.2%	53.0%	49.5%	57.8%	90.3%	77.4%	95.9%	60.4%	43.1%	75.6%	97.1%	89.1%	99.3%	88.9%	86.8%	94.0%
14.4	85.2%	60.7%	95.8%	63.5%	49.1%	76.1%	53.0%	49.5%	57.8%	90.3%	77.4%	95.9%	60.4%	43.0%	75.5%	97.1%	89.0%	99.2%	88.9%	86.8%	94.0%
14.5	85.2%	60.7%	95.8%	63.4%	49.0%	76.0%	53.0%	49.5%	57.8%	90.3%	77.4%	95.9%	60.3%	43.0%	75.5%	97.1%	88.9%	99.2%	88.9%	86.8%	93.9%
14.6	85.2%	60.7%	95.8%	63.3%	48.9%	76.0%	53.0%	49.5%	57.7%	90.3%	77.4%	95.9%	60.3%	42.9%	75.4%	97.0%	88.8%	99.2%	88.9%	86.8%	93.8%
14.7	85.2%	60.7%	95.8%	63.1%	48.7%	75.9%	53.0%	49.5%	57.7%	90.3%	77.4%	95.9%	60.2%	42.9%	75.4%	97.0%	88.8%	99.2%	88.9%	86.8%	93.7%
14.8	85.2%	60.7%	95.8%	63.0%	48.6%	75.8%	53.0%	49.5%	57.6%	90.3%	77.4%	95.9%	60.2%	42.8%	75.4%	97.0%	88.7%	99.2%	88.9%	86.8%	93.7%
14.9	85.2%	60.7%	95.8%	62.9%	48.5%	75.7%	53.0%	49.5%	57.6%	90.3%	77.4%	95.9%	60.2%	42.8%	75.3%	97.0%	88.6%	99.2%	88.9%	86.8%	93.6%
15	85.2%	60.7%	95.8%	62.8%	48.3%	75.6%	53.0%	49.5%	57.6%	90.3%	77.4%	95.9%	60.1%	42.7%	75.3%	96.9%	88.5%	99.2%	88.9%	86.8%	93.6%
15.1	85.2%	60.7%	95.8%	62.7%	48.2%	75.5%	53.0%	49.5%	57.6%	90.3%	77.4%	95.9%	60.1%	42.7%	75.3%	96.9%	88.4%	99.2%	88.9%	86.7%	93.6%
15.2	85.2%	60.7%	95.8%	62.6%	48.1%	75.5%	53.0%	49.4%	57.5%	90.3%	77.4%	95.9%	60.1%	42.7%	75.3%	96.9%	88.3%	99.2%	88.9%	86.7%	93.6%
15.3	85.2%	60.7%	95.8%	62.5%	48.0%	75.4%	53.0%	49.3%	57.5%	90.3%	77.4%	95.9%	60.0%	42.7%	75.2%	96.9%	88.2%	99.2%	88.9%	86.7%	93.6%

15.4	85.2%	60.7%	95.8%	62.4%	47.8%	75.3%	53.0%	49.2%	57.5%	90.3%	77.4%	95.9%	60.0%	42.6%	75.2%	96.8%	88.2%	99.2%	88.9%	86.7%	93.5%
15.5	85.2%	60.7%	95.8%	62.3%	47.7%	75.2%	53.0%	49.2%	57.3%	90.3%	77.4%	95.9%	60.0%	42.6%	75.2%	96.8%	88.1%	99.2%	88.9%	86.7%	93.5%
15.6	85.2%	60.7%	95.8%	62.1%	47.6%	75.1%	53.0%	49.1%	57.3%	90.3%	77.4%	95.9%	60.0%	42.6%	75.2%	96.8%	88.0%	99.2%	88.9%	86.7%	93.5%
15.7	85.2%	60.7%	95.8%	62.0%	47.4%	75.0%	53.0%	49.0%	57.3%	90.3%	77.4%	95.9%	60.0%	42.6%	75.1%	96.7%	88.0%	99.2%	88.9%	86.7%	93.5%
15.8	85.2%	60.7%	95.8%	61.9%	47.3%	74.9%	53.0%	49.0%	57.3%	90.3%	77.4%	95.9%	60.0%	42.6%	75.0%	96.7%	87.9%	99.2%	88.9%	86.7%	93.5%
15.9	85.2%	60.7%	95.8%	61.8%	47.2%	74.9%	53.0%	49.0%	57.2%	90.3%	77.4%	95.9%	60.0%	42.6%	75.0%	96.7%	87.8%	99.1%	88.9%	86.7%	93.4%
16	85.2%	60.7%	95.8%	61.7%	47.0%	74.8%	53.0%	48.9%	57.2%	90.3%	77.4%	95.9%	60.0%	42.6%	74.9%	96.7%	87.7%	99.1%	88.9%	86.7%	93.4%
16.1	85.2%	60.7%	95.8%	61.6%	46.9%	74.7%	53.0%	48.9%	57.2%	90.3%	77.4%	95.9%	60.0%	42.6%	74.9%	96.6%	87.6%	99.1%	88.9%	86.7%	93.3%
16.2	85.2%	60.7%	95.8%	61.5%	46.8%	74.6%	53.0%	48.9%	57.1%	90.3%	77.4%	95.9%	60.0%	42.5%	74.8%	96.6%	87.5%	99.1%	88.9%	86.7%	93.3%
16.3	85.2%	60.7%	95.8%	61.4%	46.7%	74.5%	53.0%	48.8%	57.1%	90.3%	77.4%	95.9%	60.0%	42.5%	74.8%	96.6%	87.4%	99.1%	88.9%	86.7%	93.3%
16.4	85.2%	60.7%	95.8%	61.2%	46.5%	74.4%	53.0%	48.8%	57.0%	90.3%	77.4%	95.9%	60.0%	42.5%	74.8%	96.5%	87.2%	99.1%	88.9%	86.7%	93.2%
16.5	85.2%	60.7%	95.8%	61.1%	46.4%	74.3%	53.0%	48.8%	57.0%	90.3%	77.4%	95.9%	60.0%	42.5%	74.8%	96.5%	87.2%	99.1%	88.9%	86.7%	93.2%
16.6	85.2%	60.7%	95.8%	61.0%	46.3%	74.2%	53.0%	48.7%	57.0%	90.3%	77.4%	95.9%	60.0%	42.5%	74.8%	96.4%	87.1%	99.1%	88.9%	86.7%	93.2%
16.7	85.2%	60.7%	95.8%	60.9%	46.2%	74.2%	53.0%	48.6%	57.0%	90.3%	77.4%	95.9%	60.0%	42.5%	74.7%	96.4%	87.1%	99.1%	88.9%	86.7%	93.1%
16.8	85.2%	60.7%	95.8%	60.8%	46.0%	74.1%	53.0%	48.6%	57.0%	90.3%	77.4%	95.9%	60.0%	42.5%	74.7%	96.4%	87.0%	99.1%	88.9%	86.7%	93.1%
16.9	85.2%	60.7%	95.8%	60.7%	45.9%	74.0%	53.0%	48.6%	57.0%	90.3%	77.3%	95.9%	60.0%	42.5%	74.6%	96.3%	86.9%	99.1%	88.9%	86.7%	93.1%
17	85.2%	60.7%	95.8%	60.6%	45.8%	73.9%	53.0%	48.5%	56.9%	90.3%	77.2%	95.9%	60.0%	42.5%	74.6%	96.3%	86.8%	99.1%	88.9%	86.7%	93.1%
17.1	85.2%	60.7%	95.8%	60.4%	45.6%	73.8%	53.0%	48.5%	56.9%	90.3%	77.2%	95.9%	60.0%	42.4%	74.6%	96.3%	86.7%	99.1%	88.9%	86.7%	93.1%
17.2	85.2%	60.7%	95.8%	60.3%	45.5%	73.7%	53.0%	48.4%	56.9%	90.3%	77.1%	95.9%	60.0%	42.4%	74.6%	96.2%	86.6%	99.1%	88.9%	86.7%	93.1%
17.3	85.2%	60.7%	95.8%	60.2%	45.4%	73.6%	53.0%	48.4%	56.8%	90.3%	77.1%	95.9%	60.0%	42.3%	74.6%	96.2%	86.5%	99.1%	88.9%	86.7%	93.1%
17.4	85.2%	60.7%	95.8%	60.1%	45.3%	73.5%	53.0%	48.3%	56.7%	90.3%	77.1%	95.9%	60.0%	42.3%	74.6%	96.1%	86.4%	99.0%	88.9%	86.7%	93.1%
17.5	85.2%	60.7%	95.8%	60.0%	45.1%	73.5%	53.0%	48.3%	56.7%	90.2%	77.1%	95.9%	60.0%	42.2%	74.5%	96.1%	86.3%	99.0%	88.9%	86.7%	93.1%
17.6	85.2%	60.7%	95.8%	59.9%	45.0%	73.4%	53.0%	48.2%	56.7%	90.2%	77.1%	95.9%	60.0%	42.2%	74.5%	96.0%	86.2%	99.0%	88.9%	86.7%	93.1%
17.7	85.2%	60.7%	95.8%	59.8%	44.9%	73.3%	53.0%	48.1%	56.7%	90.2%	77.1%	95.9%	60.0%	42.2%	74.5%	96.0%	86.1%	99.0%	88.9%	86.7%	93.1%
17.8	85.2%	60.7%	95.8%	59.6%	44.7%	73.2%	53.0%	48.1%	56.6%	90.2%	77.0%	95.9%	59.9%	42.1%	74.4%	95.9%	86.0%	99.0%	88.9%	86.7%	93.1%
17.9	85.2%	60.7%	95.8%	59.5%	44.6%	73.1%	53.0%	48.0%	56.6%	90.2%	77.0%	95.9%	59.9%	42.1%	74.4%	95.9%	85.9%	99.0%	88.9%	86.7%	93.1%
18	85.2%	60.7%	95.8%	59.4%	44.5%	73.0%	53.0%	48.0%	56.6%	90.2%	77.0%	95.9%	59.9%	42.1%	74.3%	95.9%	85.8%	99.0%	88.9%	86.7%	93.1%
18.1	85.2%	60.7%	95.8%	59.3%	44.4%	72.9%	53.0%	47.9%	56.6%	90.2%	77.0%	95.9%	59.9%	42.1%	74.3%	95.8%	85.7%	99.0%	88.9%	86.6%	93.1%
18.2	85.2%	60.7%	95.8%	59.2%	44.2%	72.8%	53.0%	47.9%	56.6%	90.2%	77.0%	95.9%	59.8%	42.0%	74.3%	95.8%	85.6%	99.0%	88.9%	86.6%	93.1%
18.3	85.2%	60.7%	95.8%	59.1%	44.1%	72.7%	53.0%	47.9%	56.6%	90.2%	77.0%	95.9%	59.8%	41.9%	74.2%	95.7%	85.5%	99.0%	88.9%	86.6%	93.1%
18.4	85.2%	60.7%	95.8%	58.9%	44.0%	72.6%	53.0%	47.8%	56.6%	90.2%	77.0%	95.9%	59.8%	41.7%	74.2%	95.7%	85.4%	99.0%	88.9%	86.6%	93.1%
18.5	85.2%	60.7%	95.8%	58.8%	43.9%	72.5%	53.0%	47.8%	56.6%	90.2%	77.0%	95.9%	59.7%	41.7%	74.2%	95.6%	85.3%	99.0%	88.9%	86.6%	93.0%

18.6	85.2%	60.7%	95.8%	58.7%	43.7%	72.4%	53.0%	47.8%	56.5%	90.2%	77.0%	95.9%	59.7%	41.7%	74.1%	95.6%	85.2%	99.0%	88.9%	86.5%	93.0%
18.7	85.2%	60.7%	95.8%	58.6%	43.6%	72.4%	53.0%	47.8%	56.5%	90.2%	77.0%	95.9%	59.7%	41.6%	74.1%	95.5%	85.1%	98.9%	88.9%	86.5%	93.0%
18.8	85.2%	60.7%	95.8%	58.5%	43.5%	72.3%	53.0%	47.7%	56.4%	90.1%	77.0%	95.9%	59.6%	41.6%	74.1%	95.5%	85.0%	98.9%	88.9%	86.5%	93.0%
18.9	85.2%	60.7%	95.8%	58.3%	43.3%	72.2%	53.0%	47.7%	56.4%	90.1%	77.0%	95.9%	59.6%	41.5%	74.0%	95.4%	84.9%	98.9%	88.9%	86.5%	93.0%
19	85.2%	60.7%	95.8%	58.2%	43.2%	72.1%	53.0%	47.7%	56.4%	90.1%	76.9%	95.8%	59.5%	41.5%	74.0%	95.4%	84.8%	98.9%	88.9%	86.5%	93.0%
19.1	85.2%	60.7%	95.8%	58.1%	43.1%	72.0%	53.0%	47.7%	56.3%	90.1%	76.9%	95.8%	59.5%	41.5%	73.9%	95.3%	84.6%	98.9%	88.9%	86.5%	93.0%
19.2	85.2%	60.7%	95.8%	58.0%	43.0%	71.9%	53.0%	47.6%	56.3%	90.1%	76.9%	95.8%	59.4%	41.4%	73.9%	95.3%	84.5%	98.9%	88.9%	86.5%	93.0%
19.3	85.2%	60.7%	95.8%	57.9%	42.8%	71.8%	53.0%	47.6%	56.3%	90.1%	76.9%	95.8%	59.4%	41.4%	73.8%	95.2%	84.4%	98.9%	88.9%	86.4%	93.0%
19.4	85.2%	60.7%	95.8%	57.7%	42.7%	71.7%	53.0%	47.6%	56.3%	90.1%	76.9%	95.8%	59.3%	41.3%	73.8%	95.2%	84.2%	98.9%	88.9%	86.4%	93.0%
19.5	85.2%	60.7%	95.8%	57.6%	42.6%	71.6%	53.0%	47.5%	56.3%	90.1%	76.9%	95.8%	59.3%	41.2%	73.7%	95.1%	84.1%	98.9%	88.9%	86.4%	93.0%
19.6	85.2%	60.7%	95.8%	57.5%	42.4%	71.5%	53.0%	47.5%	56.3%	90.0%	76.8%	95.8%	59.2%	41.1%	73.7%	95.1%	83.9%	98.9%	88.9%	86.3%	93.0%
19.7	85.2%	60.7%	95.8%	57.4%	42.3%	71.4%	53.0%	47.5%	56.3%	90.0%	76.8%	95.8%	59.2%	40.9%	73.6%	95.0%	83.8%	98.9%	88.9%	86.3%	93.0%
19.8	85.2%	60.7%	95.8%	57.2%	42.2%	71.3%	53.0%	47.4%	56.3%	90.0%	76.7%	95.8%	59.1%	40.8%	73.5%	95.0%	83.6%	98.9%	88.9%	86.2%	93.0%
19.9	85.2%	60.7%	95.8%	57.1%	42.1%	71.2%	53.0%	47.4%	56.2%	90.0%	76.6%	95.8%	59.0%	40.8%	73.5%	94.9%	83.5%	98.9%	88.9%	86.2%	93.0%
20	85.2%	60.7%	95.8%	57.0%	41.9%	71.1%	53.0%	47.3%	56.2%	90.0%	76.5%	95.8%	59.0%	40.7%	73.4%	94.9%	83.3%	98.9%	88.9%	86.2%	93.0%
20.1	85.2%	60.7%	95.8%	56.9%	41.8%	71.0%	53.0%	47.3%	56.1%	90.0%	76.5%	95.8%	58.9%	40.6%	73.3%	94.8%	83.2%	98.9%	88.9%	86.2%	93.0%
20.2	85.2%	60.7%	95.8%	56.7%	41.7%	70.8%	53.0%	47.2%	56.1%	89.9%	76.4%	95.7%	58.8%	40.5%	73.2%	94.8%	83.0%	98.9%	88.9%	86.2%	93.0%
20.3	85.2%	60.7%	95.8%	56.6%	41.6%	70.7%	53.0%	47.2%	56.1%	89.9%	76.3%	95.7%	58.7%	40.5%	73.1%	94.8%	82.9%	98.9%	88.9%	86.1%	93.0%
20.4	85.2%	60.7%	95.8%	56.5%	41.4%	70.6%	53.0%	47.1%	56.1%	89.9%	76.2%	95.7%	58.7%	40.4%	73.0%	94.7%	82.7%	98.9%	88.9%	86.1%	93.0%
20.5	85.2%	60.7%	95.8%	56.4%	41.3%	70.5%	53.0%	47.1%	56.1%	89.9%	76.2%	95.7%	58.6%	40.3%	72.9%	94.7%	82.6%	98.8%	88.9%	86.0%	93.0%
20.6	85.2%	60.7%	95.8%	56.2%	41.2%	70.4%	53.0%	47.1%	56.1%	89.9%	76.2%	95.7%	58.5%	40.2%	72.8%	94.6%	82.4%	98.8%	88.9%	86.0%	93.0%
20.7	85.2%	60.7%	95.8%	56.1%	41.0%	70.3%	53.0%	47.0%	56.1%	89.9%	76.1%	95.7%	58.4%	40.2%	72.7%	94.6%	82.3%	98.8%	88.9%	86.0%	93.0%
20.8	85.2%	60.7%	95.8%	56.0%	40.9%	70.2%	53.0%	46.9%	56.1%	89.8%	76.1%	95.7%	58.3%	40.1%	72.7%	94.5%	82.2%	98.8%	88.9%	86.0%	93.0%
20.9	85.2%	60.7%	95.8%	55.8%	40.8%	70.1%	53.0%	46.8%	56.1%	89.8%	76.0%	95.7%	58.2%	40.0%	72.6%	94.5%	82.2%	98.8%	88.9%	86.0%	93.0%
21	85.2%	60.7%	95.8%	55.7%	40.7%	70.0%	53.0%	46.8%	56.1%	89.8%	75.9%	95.7%	58.1%	39.9%	72.5%	94.4%	82.1%	98.8%	88.9%	86.0%	93.0%
21.1	85.2%	60.7%	95.8%	55.6%	40.5%	69.9%	53.0%	46.7%	56.1%	89.8%	75.9%	95.7%	58.0%	39.8%	72.5%	94.4%	82.1%	98.8%	88.9%	86.0%	93.0%
21.2	85.2%	60.7%	95.8%	55.5%	40.4%	69.8%	53.0%	46.5%	56.0%	89.8%	75.8%	95.6%	57.9%	39.7%	72.4%	94.4%	82.1%	98.8%	88.9%	86.0%	93.0%
21.3	85.2%	60.7%	95.8%	55.3%	40.3%	69.7%	53.0%	46.4%	56.0%	89.7%	75.7%	95.6%	57.8%	39.6%	72.3%	94.3%	82.0%	98.8%	88.9%	86.0%	93.0%
21.4	85.2%	60.7%	95.8%	55.2%	40.2%	69.5%	53.0%	46.3%	56.0%	89.7%	75.7%	95.6%	57.7%	39.5%	72.3%	94.3%	82.0%	98.8%	88.9%	86.0%	93.0%
21.5	85.2%	60.7%	95.8%	55.1%	40.0%	69.4%	53.0%	46.1%	56.0%	89.7%	75.6%	95.6%	57.6%	39.4%	72.2%	94.2%	81.9%	98.8%	88.9%	86.0%	93.0%
21.6	85.2%	60.7%	95.8%	54.9%	39.9%	69.3%	52.9%	46.0%	56.0%	89.7%	75.6%	95.6%	57.5%	39.3%	72.1%	94.2%	81.9%	98.7%	88.9%	86.0%	93.0%
21.7	85.2%	60.7%	95.8%	54.8%	39.8%	69.2%	52.9%	45.9%	55.9%	89.6%	75.5%	95.6%	57.4%	39.2%	72.0%	94.2%	81.8%	98.7%	88.9%	86.0%	93.0%

21.8	85.2%	60.7%	95.8%	54.7%	39.6%	69.1%	52.9%	45.7%	55.9%	89.6%	75.4%	95.6%	57.3%	39.0%	72.0%	94.1%	81.7%	98.7%	88.9%	86.0%	93.0%
21.9	85.2%	60.7%	95.8%	54.5%	39.5%	69.0%	52.9%	45.7%	55.9%	89.6%	75.4%	95.6%	57.2%	38.9%	71.9%	94.1%	81.7%	98.7%	88.9%	86.0%	93.0%
22	85.2%	60.7%	95.8%	54.4%	39.4%	68.9%	52.8%	45.6%	55.9%	89.6%	75.3%	95.6%	57.1%	38.7%	71.8%	94.0%	81.6%	98.7%	88.9%	86.0%	93.0%
22.1	85.2%	60.7%	95.8%	54.3%	39.3%	68.7%	52.8%	45.4%	55.8%	89.5%	75.2%	95.5%	57.0%	38.6%	71.7%	94.0%	81.6%	98.7%	88.9%	86.0%	93.0%
22.2	85.2%	60.7%	95.8%	54.1%	39.2%	68.6%	52.8%	45.3%	55.8%	89.5%	75.2%	95.5%	56.8%	38.4%	71.6%	94.0%	81.5%	98.7%	88.9%	86.0%	93.0%
22.3	85.2%	60.7%	95.8%	54.0%	39.0%	68.5%	52.7%	45.2%	55.7%	89.5%	75.1%	95.5%	56.7%	38.3%	71.5%	93.9%	81.4%	98.7%	88.9%	86.0%	93.0%
22.4	85.2%	60.7%	95.8%	53.9%	38.9%	68.4%	52.7%	45.1%	55.7%	89.5%	75.0%	95.5%	56.6%	38.2%	71.4%	93.9%	81.3%	98.7%	88.9%	86.0%	93.0%
22.5	85.2%	60.7%	95.8%	53.7%	38.8%	68.3%	52.6%	45.0%	55.6%	89.4%	75.0%	95.5%	56.5%	38.0%	71.3%	93.8%	81.2%	98.7%	88.9%	86.0%	93.0%
22.6	85.2%	60.7%	95.8%	53.6%	38.7%	68.2%	52.6%	44.9%	55.5%	89.4%	74.9%	95.5%	56.3%	37.9%	71.2%	93.8%	81.1%	98.7%	88.9%	86.0%	92.9%
22.7	85.2%	60.7%	95.8%	53.5%	38.6%	68.0%	52.5%	44.8%	55.5%	89.4%	74.8%	95.5%	56.2%	37.7%	71.1%	93.8%	81.0%	98.7%	88.9%	86.0%	92.9%
22.8	85.2%	60.7%	95.8%	53.3%	38.5%	67.9%	52.5%	44.6%	55.4%	89.3%	74.8%	95.5%	56.1%	37.6%	70.9%	93.7%	80.9%	98.7%	88.9%	86.0%	92.9%
22.9	85.2%	60.7%	95.8%	53.2%	38.4%	67.8%	52.4%	44.5%	55.4%	89.3%	74.7%	95.5%	55.9%	37.4%	70.8%	93.7%	80.8%	98.7%	88.9%	86.0%	92.9%
23	85.2%	60.7%	95.8%	53.0%	38.2%	67.7%	52.3%	44.4%	55.3%	89.3%	74.6%	95.4%	55.8%	37.3%	70.7%	93.7%	80.7%	98.7%	88.9%	86.0%	92.9%
23.1	85.2%	60.7%	95.8%	52.9%	38.1%	67.5%	52.3%	44.2%	55.2%	89.3%	74.5%	95.4%	55.7%	37.1%	70.6%	93.6%	80.6%	98.7%	88.9%	86.0%	92.9%
23.2	85.2%	60.7%	95.8%	52.8%	38.0%	67.4%	52.2%	44.1%	55.2%	89.2%	74.5%	95.4%	55.5%	37.0%	70.5%	93.6%	80.5%	98.6%	88.9%	86.0%	92.9%
23.3	85.2%	60.7%	95.8%	52.6%	37.9%	67.3%	52.1%	44.0%	55.1%	89.2%	74.4%	95.4%	55.4%	36.8%	70.4%	93.6%	80.5%	98.6%	88.9%	86.0%	92.9%
23.4	85.2%	60.7%	95.8%	52.5%	37.8%	67.2%	52.1%	43.9%	55.0%	89.2%	74.3%	95.4%	55.2%	36.7%	70.3%	93.5%	80.4%	98.6%	88.9%	86.0%	92.9%
23.5	85.2%	60.7%	95.8%	52.3%	37.6%	67.1%	52.0%	43.7%	54.9%	89.1%	74.3%	95.4%	55.1%	36.6%	70.2%	93.5%	80.3%	98.6%	88.9%	86.0%	92.9%
23.6	85.2%	60.7%	95.8%	52.2%	37.5%	66.9%	51.9%	43.7%	54.8%	89.1%	74.2%	95.4%	54.9%	36.4%	70.1%	93.5%	80.2%	98.6%	88.9%	86.0%	92.9%
23.7	85.2%	60.7%	95.8%	52.1%	37.4%	66.8%	51.8%	43.6%	54.6%	89.1%	74.1%	95.4%	54.7%	36.3%	70.0%	93.5%	80.1%	98.6%	88.9%	86.0%	92.9%
23.8	85.2%	60.7%	95.8%	51.9%	37.3%	66.7%	51.7%	43.6%	54.5%	89.0%	74.1%	95.3%	54.6%	36.2%	69.8%	93.4%	80.0%	98.6%	88.9%	86.0%	92.9%
23.9	85.2%	60.7%	95.8%	51.8%	37.2%	66.5%	51.7%	43.5%	54.4%	89.0%	74.0%	95.3%	54.4%	36.0%	69.7%	93.4%	79.9%	98.6%	88.9%	86.0%	92.9%
24	85.2%	60.7%	95.8%	51.6%	37.0%	66.4%	51.6%	43.4%	54.3%	89.0%	73.9%	95.3%	54.3%	35.9%	69.6%	93.4%	79.9%	98.6%	88.9%	86.0%	92.9%
24.1	85.2%	60.7%	95.8%	51.5%	36.9%	66.3%	51.5%	43.2%	54.2%	88.9%	73.8%	95.3%	54.1%	35.8%	69.4%	93.3%	79.8%	98.6%	88.9%	86.0%	92.9%
24.2	85.2%	60.7%	95.8%	51.4%	36.8%	66.2%	51.4%	43.1%	54.1%	88.9%	73.8%	95.3%	53.9%	35.6%	69.3%	93.3%	79.7%	98.6%	88.9%	86.0%	92.9%
24.3	85.2%	60.7%	95.8%	51.2%	36.7%	66.0%	51.3%	43.1%	54.0%	88.8%	73.7%	95.3%	53.8%	35.5%	69.1%	93.3%	79.6%	98.6%	88.9%	86.0%	92.9%
24.4	85.2%	60.7%	95.8%	51.1%	36.6%	65.9%	51.2%	43.0%	53.9%	88.8%	73.6%	95.3%	53.6%	35.4%	69.0%	93.3%	79.6%	98.6%	88.9%	86.0%	92.9%
24.5	85.2%	60.7%	95.8%	50.9%	36.4%	65.8%	51.1%	42.9%	53.8%	88.8%	73.6%	95.3%	53.4%	35.2%	68.8%	93.2%	79.5%	98.6%	88.9%	86.0%	92.9%
24.6	85.2%	60.7%	95.8%	50.8%	36.3%	65.6%	50.9%	42.8%	53.7%	88.7%	73.5%	95.2%	53.2%	35.1%	68.7%	93.2%	79.4%	98.6%	88.9%	86.0%	92.9%
24.7	85.2%	60.7%	95.8%	50.6%	36.2%	65.5%	50.8%	42.7%	53.5%	88.7%	73.4%	95.2%	53.0%	34.9%	68.5%	93.2%	79.3%	98.6%	88.9%	86.0%	92.9%
24.8	85.2%	60.7%	95.8%	50.5%	36.1%	65.4%	50.7%	42.6%	53.4%	88.6%	73.3%	95.2%	52.9%	34.8%	68.3%	93.2%	79.2%	98.5%	88.9%	86.0%	92.8%
24.9	85.2%	60.7%	95.8%	50.4%	36.0%	65.2%	50.6%	42.5%	53.2%	88.6%	73.2%	95.2%	52.7%	34.7%	68.2%	93.2%	79.0%	98.5%	88.9%	86.0%	92.8%

25	85.2%	60.7%	95.8%	50.2%	35.8%	65.1%	50.5%	42.4%	53.1%	88.6%	73.2%	95.2%	52.5%	34.5%	68.0%	93.1%	79.0%	98.5%	88.9%	86.0%	92.8%
25.1	85.2%	60.7%	95.8%	50.1%	35.7%	65.0%	50.4%	42.4%	52.9%	88.5%	73.1%	95.2%	52.3%	34.3%	67.8%	93.1%	79.0%	98.5%	88.9%	86.0%	92.8%
25.2	85.2%	60.7%	95.8%	49.9%	35.6%	64.8%	50.2%	42.3%	52.8%	88.5%	73.0%	95.1%	52.1%	34.1%	67.6%	93.1%	78.9%	98.5%	88.9%	86.0%	92.8%
25.3	85.2%	60.7%	95.8%	49.8%	35.5%	64.7%	50.1%	42.2%	52.7%	88.4%	72.9%	95.1%	51.9%	33.9%	67.5%	93.1%	78.8%	98.5%	88.9%	85.9%	92.8%
25.4	85.2%	60.7%	95.8%	49.6%	35.3%	64.5%	50.0%	42.1%	52.5%	88.4%	72.8%	95.1%	51.7%	33.7%	67.3%	93.1%	78.8%	98.5%	88.9%	85.9%	92.8%
25.5	85.2%	60.7%	95.8%	49.5%	35.2%	64.4%	49.8%	42.0%	52.3%	88.3%	72.7%	95.1%	51.5%	33.5%	67.1%	93.1%	78.7%	98.5%	88.9%	85.9%	92.8%
25.6	85.2%	60.7%	95.8%	49.3%	35.1%	64.3%	49.7%	41.9%	52.2%	88.3%	72.6%	95.1%	51.3%	33.3%	66.9%	93.0%	78.7%	98.5%	88.9%	85.9%	92.8%
25.7	85.2%	60.7%	95.8%	49.2%	35.0%	64.1%	49.6%	41.8%	52.0%	88.3%	72.5%	95.0%	51.1%	33.2%	66.7%	93.0%	78.6%	98.5%	88.9%	85.9%	92.8%
25.8	85.2%	60.7%	95.7%	49.0%	34.8%	64.0%	49.4%	41.7%	51.8%	88.2%	72.4%	95.0%	50.9%	33.0%	66.5%	93.0%	78.5%	98.5%	88.9%	85.9%	92.8%
25.9	85.2%	60.6%	95.7%	48.9%	34.7%	63.8%	49.3%	41.6%	51.7%	88.2%	72.3%	95.0%	50.7%	32.8%	66.3%	93.0%	78.5%	98.4%	88.9%	85.9%	92.8%
26	85.2%	60.6%	95.7%	48.7%	34.6%	63.7%	49.1%	41.6%	51.5%	88.1%	72.2%	95.0%	50.5%	32.7%	66.1%	93.0%	78.5%	98.4%	88.9%	85.9%	92.8%
26.1	85.2%	60.6%	95.7%	48.6%	34.5%	63.6%	49.0%	41.5%	51.3%	88.1%	72.1%	95.0%	50.2%	32.5%	66.0%	93.0%	78.5%	98.4%	88.9%	85.9%	92.8%
26.2	85.1%	60.6%	95.7%	48.4%	34.3%	63.4%	48.8%	41.4%	51.2%	88.0%	72.0%	95.0%	50.0%	32.4%	65.8%	93.0%	78.5%	98.4%	88.9%	85.9%	92.8%
26.3	85.1%	60.6%	95.7%	48.3%	34.2%	63.3%	48.6%	41.4%	51.0%	88.0%	72.0%	94.9%	49.8%	32.2%	65.6%	93.0%	78.4%	98.4%	88.9%	85.9%	92.8%
26.4	85.1%	60.5%	95.7%	48.1%	34.1%	63.1%	48.5%	41.3%	50.9%	87.9%	71.9%	94.9%	49.6%	32.1%	65.4%	92.9%	78.3%	98.4%	88.9%	85.9%	92.8%
26.5	85.1%	60.5%	95.7%	48.0%	34.0%	63.0%	48.3%	41.3%	50.7%	87.9%	71.9%	94.9%	49.4%	31.9%	65.2%	92.9%	78.2%	98.4%	88.9%	85.9%	92.8%
26.6	85.1%	60.5%	95.7%	47.8%	33.8%	62.8%	48.1%	41.2%	50.6%	87.8%	71.8%	94.9%	49.1%	31.7%	65.0%	92.9%	78.2%	98.4%	88.9%	85.9%	92.8%
26.7	85.1%	60.5%	95.7%	47.7%	33.7%	62.7%	48.0%	41.1%	50.4%	87.8%	71.7%	94.9%	48.9%	31.6%	64.8%	92.9%	78.1%	98.4%	88.9%	85.9%	92.8%
26.8	85.1%	60.5%	95.7%	47.5%	33.6%	62.5%	47.8%	41.1%	50.3%	87.7%	71.6%	94.8%	48.7%	31.4%	64.7%	92.9%	78.1%	98.4%	88.9%	85.9%	92.8%
26.9	85.1%	60.4%	95.7%	47.4%	33.4%	62.4%	47.6%	41.0%	50.1%	87.7%	71.5%	94.8%	48.4%	31.3%	64.5%	92.9%	78.1%	98.4%	88.9%	85.9%	92.8%
27	85.1%	60.4%	95.7%	47.2%	33.3%	62.3%	47.4%	41.0%	49.9%	87.6%	71.4%	94.8%	48.2%	31.1%	64.3%	92.9%	78.1%	98.4%	88.9%	85.9%	92.8%
27.1	85.1%	60.4%	95.7%	47.1%	33.2%	62.1%	47.3%	40.9%	49.9%	87.6%	71.3%	94.8%	48.0%	30.9%	64.2%	92.9%	78.0%	98.4%	88.9%	85.9%	92.8%
27.2	85.0%	60.3%	95.7%	46.9%	33.1%	62.0%	47.1%	40.9%	49.6%	87.5%	71.2%	94.8%	47.8%	30.7%	64.0%	92.9%	78.0%	98.4%	88.9%	85.9%	92.8%
27.3	85.0%	60.3%	95.7%	46.8%	32.9%	61.8%	46.9%	40.8%	49.5%	87.5%	71.2%	94.8%	47.5%	30.6%	63.8%	92.9%	78.0%	98.4%	88.9%	85.9%	92.7%
27.4	85.0%	60.3%	95.7%	46.6%	32.8%	61.7%	46.7%	40.7%	49.4%	87.4%	71.1%	94.7%	47.3%	30.4%	63.7%	92.9%	78.0%	98.4%	88.9%	85.9%	92.7%
27.5	85.0%	60.3%	95.7%	46.5%	32.7%	61.5%	46.5%	40.6%	49.2%	87.3%	71.0%	94.7%	47.1%	30.2%	63.5%	92.9%	78.0%	98.4%	88.9%	85.9%	92.7%
27.6	85.0%	60.2%	95.7%	46.3%	32.5%	61.3%	46.3%	40.5%	49.1%	87.3%	70.9%	94.7%	46.9%	30.0%	63.4%	92.9%	78.0%	98.4%	88.9%	85.9%	92.7%
27.7	85.0%	60.2%	95.7%	46.2%	32.4%	61.2%	46.1%	40.5%	49.0%	87.2%	70.8%	94.7%	46.7%	29.9%	63.2%	92.9%	78.0%	98.4%	88.9%	85.9%	92.7%
27.8	85.0%	60.2%	95.7%	46.0%	32.3%	61.0%	45.9%	40.4%	48.8%	87.2%	70.6%	94.7%	46.5%	29.7%	63.1%	92.9%	78.0%	98.4%	88.9%	85.9%	92.7%
27.9	84.9%	60.1%	95.7%	45.9%	32.2%	60.9%	45.7%	40.3%	48.6%	87.1%	70.6%	94.6%	46.3%	29.6%	62.9%	92.9%	78.0%	98.4%	88.9%	85.9%	92.7%
28	84.9%	60.1%	95.7%	45.7%	32.0%	60.7%	45.5%	40.3%	48.4%	87.1%	70.5%	94.6%	46.1%	29.5%	62.8%	92.9%	78.0%	98.4%	88.9%	85.9%	92.7%
28.1	84.9%	60.0%	95.7%	45.6%	31.9%	60.6%	45.3%	40.2%	48.2%	87.0%	70.4%	94.6%	45.9%	29.3%	62.6%	92.9%	78.0%	98.4%	88.9%	85.9%	92.7%

28.2	84.9%	60.0%	95.7%	45.4%	31.8%	60.4%	45.1%	40.1%	48.1%	87.0%	70.3%	94.5%	45.7%	29.2%	62.4%	92.9%	78.0%	98.4%	88.9%	85.9%	92.7%
28.3	84.9%	60.0%	95.7%	45.2%	31.6%	60.3%	44.8%	40.0%	47.9%	86.9%	70.2%	94.5%	45.5%	29.1%	62.2%	92.9%	77.9%	98.4%	88.9%	85.9%	92.6%
28.4	84.9%	59.9%	95.7%	45.1%	31.5%	60.1%	44.6%	39.9%	47.7%	86.8%	70.1%	94.5%	45.3%	29.0%	62.0%	92.9%	77.9%	98.4%	88.9%	85.9%	92.6%
28.5	84.8%	59.9%	95.6%	44.9%	31.4%	59.9%	44.4%	39.8%	47.6%	86.8%	70.0%	94.5%	45.1%	28.9%	61.9%	92.9%	77.9%	98.4%	88.9%	85.9%	92.5%
28.6	84.8%	59.9%	95.6%	44.8%	31.2%	59.8%	44.2%	39.8%	47.5%	86.7%	69.9%	94.4%	44.9%	28.8%	61.7%	92.9%	77.8%	98.4%	88.9%	85.9%	92.5%
28.7	84.8%	59.8%	95.6%	44.6%	31.1%	59.6%	44.0%	39.6%	47.3%	86.7%	69.8%	94.4%	44.7%	28.7%	61.5%	92.9%	77.8%	98.4%	88.9%	85.9%	92.5%
28.8	84.8%	59.8%	95.6%	44.5%	31.0%	59.5%	43.8%	39.6%	47.1%	86.6%	69.7%	94.4%	44.5%	28.5%	61.3%	92.9%	77.8%	98.4%	88.9%	85.9%	92.5%
28.9	84.8%	59.8%	95.6%	44.3%	30.9%	59.3%	43.6%	39.4%	47.0%	86.6%	69.6%	94.4%	44.3%	28.4%	61.2%	92.9%	77.8%	98.4%	88.9%	85.9%	92.5%
29	84.7%	59.7%	95.6%	44.2%	30.7%	59.1%	43.4%	39.2%	46.8%	86.5%	69.5%	94.3%	44.2%	28.2%	61.0%	92.9%	77.8%	98.4%	88.9%	85.9%	92.5%
29.1	84.7%	59.7%	95.6%	44.0%	30.6%	59.0%	43.2%	39.2%	46.7%	86.4%	69.5%	94.3%	44.0%	28.1%	60.8%	92.9%	77.8%	98.4%	88.9%	85.9%	92.5%
29.2	84.7%	59.6%	95.6%	43.8%	30.5%	58.8%	43.0%	39.0%	46.6%	86.4%	69.4%	94.3%	43.8%	28.0%	60.7%	92.9%	77.8%	98.4%	88.9%	85.9%	92.4%
29.3	84.7%	59.6%	95.6%	43.7%	30.4%	58.7%	42.8%	38.8%	46.5%	86.3%	69.3%	94.2%	43.6%	27.8%	60.5%	92.9%	77.8%	98.4%	88.9%	85.9%	92.4%
29.4	84.6%	59.6%	95.6%	43.5%	30.3%	58.5%	42.7%	38.6%	46.3%	86.3%	69.2%	94.2%	43.5%	27.7%	60.4%	92.9%	77.8%	98.4%	88.9%	85.9%	92.4%
29.5	84.6%	59.5%	95.6%	43.4%	30.2%	58.4%	42.5%	38.6%	46.2%	86.2%	69.1%	94.2%	43.3%	27.6%	60.2%	92.9%	77.8%	98.4%	88.9%	85.9%	92.4%
29.6	84.6%	59.5%	95.6%	43.3%	30.1%	58.3%	42.3%	38.4%	46.0%	86.1%	69.0%	94.2%	43.1%	27.4%	60.1%	92.9%	77.8%	98.4%	88.9%	85.9%	92.4%
29.7	84.6%	59.4%	95.6%	43.1%	29.9%	58.1%	42.1%	38.2%	45.9%	86.1%	68.9%	94.1%	43.0%	27.3%	59.9%	92.9%	77.8%	98.4%	88.9%	85.9%	92.4%
29.8	84.5%	59.4%	95.6%	43.0%	29.8%	58.0%	41.9%	38.1%	45.8%	86.0%	68.8%	94.1%	42.8%	27.2%	59.7%	92.9%	77.7%	98.4%	88.9%	85.9%	92.3%
29.9	84.5%	59.3%	95.5%	42.8%	29.7%	57.9%	41.8%	37.9%	45.7%	86.0%	68.7%	94.1%	42.7%	27.1%	59.6%	92.9%	77.5%	98.3%	88.9%	85.9%	92.3%
30	84.5%	59.3%	95.5%	42.7%	29.6%	57.8%	41.6%	37.7%	45.6%	85.9%	68.7%	94.1%	42.5%	27.0%	59.5%	92.9%	77.4%	98.3%	88.9%	85.9%	92.3%
30.1	84.4%	59.2%	95.5%	42.6%	29.5%	57.6%	41.4%	37.6%	45.4%	85.8%	68.6%	94.0%	42.3%	26.8%	59.3%	92.9%	77.4%	98.3%	88.9%	85.9%	92.3%
30.2	84.4%	59.2%	95.5%	42.4%	29.4%	57.5%	41.3%	37.4%	45.4%	85.8%	68.5%	94.0%	42.2%	26.7%	59.2%	92.9%	77.3%	98.3%	88.9%	85.9%	92.3%
30.3	84.4%	59.1%	95.5%	42.3%	29.3%	57.4%	41.1%	37.3%	45.3%	85.7%	68.4%	94.0%	42.1%	26.6%	59.0%	92.9%	77.2%	98.3%	88.9%	85.9%	92.3%
30.4	84.4%	59.1%	95.5%	42.1%	29.2%	57.3%	41.0%	37.2%	45.1%	85.7%	68.3%	93.9%	41.9%	26.5%	58.9%	92.9%	77.1%	98.3%	88.9%	85.9%	92.2%
30.5	84.3%	59.0%	95.5%	42.0%	29.1%	57.1%	40.8%	37.0%	45.0%	85.6%	68.2%	93.9%	41.8%	26.4%	58.8%	92.9%	77.0%	98.3%	88.9%	85.9%	92.2%
30.6	84.3%	59.0%	95.5%	41.9%	29.0%	57.0%	40.7%	36.9%	44.9%	85.5%	68.1%	93.9%	41.6%	26.3%	58.6%	92.9%	76.9%	98.3%	88.9%	85.9%	92.2%
30.7	84.3%	58.9%	95.5%	41.8%	28.9%	56.9%	40.5%	36.7%	44.9%	85.5%	67.9%	93.9%	41.5%	26.2%	58.5%	92.9%	76.8%	98.3%	88.9%	85.9%	92.1%
30.8	84.2%	58.8%	95.5%	41.6%	28.8%	56.8%	40.4%	36.6%	44.8%	85.4%	67.8%	93.8%	41.4%	26.1%	58.3%	92.9%	76.7%	98.3%	88.9%	85.9%	92.1%
30.9	84.2%	58.8%	95.5%	41.5%	28.7%	56.7%	40.3%	36.5%	44.6%	85.3%	67.7%	93.8%	41.2%	26.0%	58.2%	92.9%	76.6%	98.3%	88.9%	85.9%	92.1%
31	84.2%	58.7%	95.4%	41.4%	28.6%	56.6%	40.1%	36.3%	44.5%	85.3%	67.6%	93.8%	41.1%	25.9%	58.1%	92.8%	76.6%	98.3%	88.9%	85.9%	92.1%
31.1	84.1%	58.7%	95.4%	41.3%	28.6%	56.5%	40.0%	36.1%	44.4%	85.2%	67.5%	93.7%	41.0%	25.8%	57.9%	92.8%	76.6%	98.3%	88.9%	85.9%	92.1%
31.2	84.1%	58.6%	95.4%	41.1%	28.5%	56.3%	39.9%	36.0%	44.3%	85.2%	67.4%	93.7%	40.8%	25.7%	57.8%	92.8%	76.5%	98.3%	88.9%	85.9%	92.1%
31.3	84.1%	58.5%	95.4%	41.0%	28.4%	56.2%	39.7%	35.8%	44.2%	85.1%	67.3%	93.7%	40.7%	25.6%	57.7%	92.8%	76.4%	98.3%	88.9%	85.9%	92.1%

31.4	84.0%	58.5%	95.4%	40.9%	28.3%	56.1%	39.6%	35.7%	44.1%	85.0%	67.1%	93.7%	40.6%	25.5%	57.5%	92.8%	76.3%	98.3%	88.9%	85.9%	92.1%
31.5	84.0%	58.4%	95.4%	40.8%	28.2%	56.0%	39.5%	35.6%	44.0%	85.0%	67.0%	93.6%	40.5%	25.4%	57.4%	92.8%	76.1%	98.3%	88.9%	85.9%	92.1%
31.6	84.0%	58.4%	95.4%	40.7%	28.1%	55.9%	39.4%	35.5%	43.9%	84.9%	66.9%	93.6%	40.4%	25.4%	57.3%	92.8%	76.0%	98.3%	88.9%	85.9%	92.0%
31.7	83.9%	58.3%	95.4%	40.6%	28.0%	55.8%	39.3%	35.4%	43.9%	84.8%	66.8%	93.6%	40.3%	25.3%	57.2%	92.8%	75.9%	98.2%	88.9%	85.9%	92.0%
31.8	83.9%	58.2%	95.4%	40.4%	27.9%	55.7%	39.1%	35.2%	43.8%	84.8%	66.7%	93.6%	40.1%	25.2%	57.1%	92.8%	75.8%	98.2%	88.9%	85.9%	92.0%
31.9	83.8%	58.2%	95.3%	40.3%	27.9%	55.6%	39.0%	35.1%	43.7%	84.7%	66.6%	93.6%	40.0%	25.1%	57.0%	92.8%	75.7%	98.2%	88.9%	85.9%	92.0%
32	83.8%	58.1%	95.3%	40.2%	27.8%	55.5%	38.9%	35.0%	43.7%	84.6%	66.4%	93.5%	39.9%	25.0%	56.9%	92.7%	75.6%	98.2%	88.9%	85.9%	91.9%
32.1	83.8%	58.0%	95.3%	40.1%	27.7%	55.4%	38.8%	34.8%	43.6%	84.6%	66.3%	93.5%	39.8%	25.0%	56.8%	92.7%	75.5%	98.2%	88.9%	85.9%	91.9%
32.2	83.7%	57.9%	95.3%	40.0%	27.6%	55.3%	38.7%	34.7%	43.5%	84.5%	66.2%	93.5%	39.7%	24.9%	56.7%	92.7%	75.4%	98.2%	88.9%	85.9%	91.9%
32.3	83.7%	57.9%	95.3%	39.9%	27.5%	55.2%	38.6%	34.6%	43.5%	84.4%	66.1%	93.4%	39.6%	24.8%	56.6%	92.7%	75.3%	98.2%	88.9%	85.9%	91.9%
32.4	83.6%	57.8%	95.3%	39.8%	27.5%	55.1%	38.5%	34.5%	43.4%	84.3%	66.0%	93.4%	39.5%	24.7%	56.5%	92.7%	75.2%	98.2%	88.9%	85.9%	91.9%
32.5	83.6%	57.7%	95.3%	39.7%	27.4%	55.0%	38.4%	34.4%	43.3%	84.3%	65.9%	93.4%	39.4%	24.7%	56.4%	92.7%	75.1%	98.1%	88.9%	85.8%	91.9%
32.6	83.5%	57.7%	95.3%	39.6%	27.3%	54.9%	38.4%	34.3%	43.3%	84.2%	65.7%	93.4%	39.4%	24.6%	56.3%	92.7%	75.1%	98.1%	88.9%	85.8%	91.9%
32.7	83.5%	57.6%	95.2%	39.5%	27.2%	54.8%	38.3%	34.1%	43.2%	84.1%	65.6%	93.3%	39.3%	24.5%	56.2%	92.6%	75.0%	98.1%	88.9%	85.8%	91.9%
32.8	83.5%	57.5%	95.2%	39.4%	27.2%	54.7%	38.2%	33.9%	43.2%	84.1%	65.5%	93.3%	39.2%	24.5%	56.1%	92.6%	74.9%	98.1%	88.9%	85.8%	91.9%
32.9	83.4%	57.4%	95.2%	39.3%	27.1%	54.6%	38.1%	33.8%	43.2%	84.0%	65.3%	93.2%	39.1%	24.4%	56.0%	92.6%	74.8%	98.1%	88.9%	85.8%	91.9%
33	83.4%	57.3%	95.2%	39.2%	27.0%	54.5%	38.0%	33.8%	43.1%	83.9%	65.2%	93.2%	39.0%	24.3%	55.9%	92.6%	74.8%	98.1%	88.9%	85.8%	91.9%
33.1	83.3%	57.3%	95.2%	39.1%	27.0%	54.5%	38.0%	33.7%	43.1%	83.9%	65.1%	93.2%	38.9%	24.3%	55.8%	92.6%	74.7%	98.1%	88.9%	85.8%	91.8%
33.2	83.3%	57.2%	95.2%	39.0%	26.9%	54.4%	37.9%	33.5%	43.1%	83.8%	64.9%	93.1%	38.9%	24.2%	55.8%	92.5%	74.6%	98.1%	88.9%	85.8%	91.8%
33.3	83.2%	57.1%	95.2%	38.9%	26.8%	54.3%	37.8%	33.4%	43.0%	83.7%	64.8%	93.1%	38.8%	24.2%	55.7%	92.5%	74.5%	98.1%	88.9%	85.8%	91.8%
33.4	83.2%	57.0%	95.1%	38.9%	26.7%	54.2%	37.7%	33.2%	43.0%	83.6%	64.6%	93.1%	38.7%	24.1%	55.6%	92.5%	74.5%	98.1%	88.9%	85.8%	91.8%
33.5	83.1%	56.9%	95.1%	38.8%	26.7%	54.1%	37.7%	33.1%	42.9%	83.6%	64.5%	93.0%	38.6%	24.1%	55.5%	92.5%	74.4%	98.1%	88.9%	85.8%	91.8%
33.6	83.1%	56.8%	95.1%	38.7%	26.6%	54.0%	37.6%	33.0%	42.9%	83.5%	64.4%	93.0%	38.6%	24.0%	55.5%	92.5%	74.3%	98.1%	88.9%	85.8%	91.8%
33.7	83.0%	56.8%	95.1%	38.6%	26.6%	53.9%	37.6%	32.8%	42.8%	83.4%	64.2%	93.0%	38.5%	24.0%	55.4%	92.4%	74.3%	98.1%	88.9%	85.8%	91.8%
33.8	83.0%	56.7%	95.1%	38.5%	26.5%	53.8%	37.5%	32.7%	42.7%	83.3%	64.1%	92.9%	38.4%	23.9%	55.3%	92.4%	74.2%	98.1%	88.9%	85.8%	91.8%
33.9	82.9%	56.6%	95.1%	38.4%	26.4%	53.8%	37.5%	32.6%	42.7%	83.3%	63.9%	92.9%	38.4%	23.9%	55.2%	92.4%	74.2%	98.1%	88.9%	85.8%	91.8%
34	82.9%	56.5%	95.0%	38.4%	26.4%	53.7%	37.4%	32.5%	42.7%	83.2%	63.8%	92.9%	38.3%	23.8%	55.2%	92.4%	74.1%	98.0%	88.9%	85.8%	91.7%
34.1	82.8%	56.4%	95.0%	38.3%	26.3%	53.6%	37.4%	32.5%	42.7%	83.1%	63.6%	92.8%	38.3%	23.8%	55.1%	92.4%	74.0%	98.0%	88.9%	85.8%	91.7%
34.2	82.7%	56.3%	95.0%	38.2%	26.3%	53.5%	37.3%	32.3%	42.6%	83.0%	63.5%	92.8%	38.2%	23.8%	55.1%	92.3%	74.0%	98.0%	88.9%	85.8%	91.7%
34.3	82.7%	56.2%	95.0%	38.1%	26.2%	53.4%	37.3%	32.2%	42.6%	83.0%	63.3%	92.7%	38.2%	23.7%	55.0%	92.3%	73.9%	98.0%	88.9%	85.8%	91.7%
34.4	82.6%	56.1%	95.0%	38.0%	26.1%	53.3%	37.2%	32.1%	42.6%	82.9%	63.2%	92.7%	38.1%	23.7%	54.9%	92.3%	73.9%	98.0%	88.9%	85.8%	91.7%
34.5	82.6%	56.0%	95.0%	38.0%	26.1%	53.2%	37.2%	32.0%	42.6%	82.8%	63.1%	92.7%	38.1%	23.7%	54.9%	92.3%	73.8%	98.0%	88.9%	85.8%	91.7%

34.6	82.5%	55.9%	94.9%	37.9%	26.0%	53.2%	37.2%	31.9%	42.6%	82.7%	62.9%	92.6%	38.0%	23.6%	54.8%	92.2%	73.8%	98.0%	88.9%	85.8%	91.7%
34.7	82.5%	55.8%	94.9%	37.8%	26.0%	53.1%	37.2%	31.8%	42.6%	82.7%	62.8%	92.6%	38.0%	23.6%	54.8%	92.2%	73.7%	98.0%	88.9%	85.8%	91.6%
34.8	82.4%	55.8%	94.9%	37.8%	25.9%	53.0%	37.1%	31.6%	42.6%	82.6%	62.6%	92.6%	37.9%	23.6%	54.7%	92.2%	73.7%	98.0%	88.9%	85.8%	91.6%
34.9	82.3%	55.6%	94.9%	37.7%	25.9%	52.9%	37.1%	31.5%	42.6%	82.5%	62.5%	92.5%	37.9%	23.5%	54.7%	92.2%	73.6%	98.0%	88.9%	85.8%	91.6%
35	82.3%	55.5%	94.9%	37.6%	25.8%	52.8%	37.1%	31.3%	42.5%	82.4%	62.3%	92.5%	37.9%	23.5%	54.6%	92.1%	73.6%	98.0%	88.9%	85.8%	91.6%
35.1	82.2%	55.4%	94.9%	37.6%	25.8%	52.8%	37.1%	31.1%	42.5%	82.4%	62.2%	92.4%	37.8%	23.5%	54.6%	92.1%	73.5%	97.9%	88.9%	85.8%	91.6%
35.2	82.2%	55.3%	94.8%	37.5%	25.7%	52.7%	37.0%	30.9%	42.5%	82.3%	62.0%	92.4%	37.8%	23.5%	54.6%	92.1%	73.5%	97.9%	88.9%	85.8%	91.5%
35.3	82.1%	55.2%	94.8%	37.5%	25.7%	52.6%	37.0%	30.7%	42.5%	82.2%	61.9%	92.4%	37.8%	23.4%	54.5%	92.0%	73.5%	97.9%	88.9%	85.8%	91.5%
35.4	82.0%	55.1%	94.8%	37.4%	25.6%	52.6%	37.0%	30.6%	42.5%	82.1%	61.7%	92.3%	37.7%	23.4%	54.5%	92.0%	73.4%	97.9%	88.9%	85.8%	91.5%
35.5	82.0%	55.0%	94.8%	37.3%	25.6%	52.5%	37.0%	30.4%	42.5%	82.0%	61.6%	92.3%	37.7%	23.3%	54.5%	92.0%	73.4%	97.9%	88.9%	85.8%	91.5%
35.6	81.9%	54.9%	94.8%	37.3%	25.5%	52.4%	37.0%	30.2%	42.5%	82.0%	61.4%	92.2%	37.7%	23.3%	54.4%	92.0%	73.4%	97.9%	88.9%	85.8%	91.4%
35.7	81.8%	54.8%	94.7%	37.2%	25.5%	52.4%	37.0%	30.0%	42.4%	81.9%	61.3%	92.2%	37.7%	23.3%	54.4%	91.9%	73.3%	97.9%	88.9%	85.8%	91.4%
35.8	81.8%	54.7%	94.7%	37.2%	25.5%	52.3%	37.0%	29.9%	42.5%	81.8%	61.1%	92.1%	37.7%	23.2%	54.4%	91.9%	73.3%	97.9%	88.9%	85.7%	91.4%
35.9	81.7%	54.6%	94.7%	37.1%	25.4%	52.3%	37.0%	29.7%	42.5%	81.7%	61.0%	92.1%	37.6%	23.2%	54.4%	91.9%	73.3%	97.9%	88.9%	85.7%	91.4%
36	81.6%	54.4%	94.7%	37.1%	25.4%	52.2%	37.0%	29.5%	42.5%	81.6%	60.8%	92.1%	37.6%	23.2%	54.3%	91.8%	73.2%	97.9%	88.9%	85.7%	91.3%
36.1	81.6%	54.3%	94.6%	37.0%	25.3%	52.2%	37.0%	29.2%	42.5%	81.5%	60.6%	92.0%	37.6%	23.2%	54.3%	91.8%	73.2%	97.9%	88.9%	85.7%	91.3%
36.2	81.5%	54.2%	94.6%	37.0%	25.3%	52.1%	37.0%	29.0%	42.5%	81.5%	60.5%	92.0%	37.6%	23.2%	54.3%	91.8%	73.1%	97.9%	88.9%	85.6%	91.3%
36.3	81.4%	54.1%	94.6%	36.9%	25.2%	52.1%	37.0%	28.8%	42.5%	81.4%	60.3%	91.9%	37.6%	23.2%	54.3%	91.7%	73.1%	97.9%	88.9%	85.6%	91.3%
36.4	81.4%	54.0%	94.6%	36.9%	25.2%	52.1%	37.0%	28.6%	42.5%	81.3%	60.2%	91.9%	37.6%	23.2%	54.3%	91.7%	73.0%	97.8%	88.9%	85.6%	91.3%
36.5	81.3%	53.9%	94.5%	36.8%	25.2%	52.0%	37.0%	28.5%	42.4%	81.2%	60.1%	91.8%	37.6%	23.2%	54.2%	91.6%	73.0%	97.8%	88.9%	85.6%	91.2%
36.6	81.2%	53.8%	94.5%	36.8%	25.1%	52.0%	37.0%	28.3%	42.4%	81.1%	60.0%	91.8%	37.6%	23.2%	54.2%	91.6%	72.9%	97.8%	88.9%	85.6%	91.2%
36.7	81.2%	53.7%	94.5%	36.7%	25.1%	51.9%	37.0%	28.1%	42.4%	81.0%	59.9%	91.8%	37.6%	23.3%	54.2%	91.6%	72.9%	97.8%	88.9%	85.6%	91.2%
36.8	81.1%	53.6%	94.5%	36.7%	25.0%	51.9%	37.0%	27.9%	42.4%	81.0%	59.8%	91.7%	37.6%	23.3%	54.1%	91.5%	72.8%	97.8%	88.9%	85.6%	91.2%
36.9	81.0%	53.5%	94.5%	36.7%	25.0%	51.8%	37.0%	27.7%	42.4%	80.9%	59.6%	91.7%	37.6%	23.3%	54.1%	91.5%	72.7%	97.8%	88.9%	85.6%	91.2%
37	80.9%	53.4%	94.4%	36.6%	25.0%	51.8%	37.0%	27.5%	42.4%	80.8%	59.5%	91.6%	37.6%	23.3%	54.1%	91.5%	72.7%	97.7%	88.9%	85.6%	91.1%
37.1	80.9%	53.3%	94.4%	36.6%	24.9%	51.8%	37.0%	27.3%	42.4%	80.7%	59.4%	91.6%	37.6%	23.3%	54.1%	91.4%	72.6%	97.7%	88.9%	85.6%	91.1%
37.2	80.8%	53.1%	94.4%	36.6%	24.9%	51.7%	37.0%	27.2%	42.4%	80.6%	59.3%	91.5%	37.7%	23.2%	54.1%	91.4%	72.6%	97.7%	88.9%	85.6%	91.1%
37.3	80.7%	53.0%	94.4%	36.5%	24.9%	51.7%	37.0%	27.0%	42.4%	80.5%	59.2%	91.5%	37.7%	23.2%	54.1%	91.3%	72.6%	97.7%	88.9%	85.5%	91.1%
37.4	80.7%	52.9%	94.3%	36.5%	24.8%	51.7%	37.0%	26.8%	42.4%	80.5%	59.1%	91.5%	37.7%	23.2%	54.1%	91.3%	72.5%	97.7%	88.9%	85.5%	91.1%
37.5	80.6%	52.8%	94.3%	36.5%	24.8%	51.7%	37.0%	26.7%	42.4%	80.4%	59.0%	91.5%	37.7%	23.2%	54.1%	91.3%	72.5%	97.7%	88.9%	85.5%	91.1%
37.6	80.5%	52.7%	94.3%	36.4%	24.8%	51.6%	37.0%	26.5%	42.4%	80.3%	58.9%	91.4%	37.7%	23.2%	54.1%	91.2%	72.4%	97.7%	88.9%	85.5%	91.1%
37.7	80.4%	52.6%	94.3%	36.4%	24.8%	51.6%	37.0%	26.4%	42.4%	80.2%	58.8%	91.4%	37.7%	23.2%	54.2%	91.2%	72.4%	97.7%	88.9%	85.5%	91.1%

37.8	80.4%	52.5%	94.2%	36.4%	24.7%	51.6%	37.0%	26.2%	42.4%	80.1%	58.7%	91.3%	37.7%	23.2%	54.2%	91.1%	72.3%	97.7%	88.9%	85.5%	91.0%
37.9	80.3%	52.3%	94.2%	36.4%	24.7%	51.5%	37.0%	26.1%	42.3%	80.0%	58.5%	91.3%	37.7%	23.2%	54.2%	91.1%	72.2%	97.7%	88.9%	85.5%	91.0%
38	80.2%	52.2%	94.2%	36.3%	24.7%	51.5%	37.0%	25.9%	42.3%	80.0%	58.4%	91.3%	37.7%	23.2%	54.2%	91.0%	72.2%	97.7%	88.9%	85.4%	91.0%
38.1	80.1%	52.1%	94.2%	36.3%	24.7%	51.5%	37.0%	25.8%	42.3%	79.9%	58.3%	91.2%	37.7%	23.2%	54.2%	91.0%	72.2%	97.7%	88.9%	85.4%	91.0%
38.2	80.1%	52.0%	94.1%	36.3%	24.6%	51.5%	37.0%	25.6%	42.3%	79.8%	58.2%	91.2%	37.8%	23.2%	54.2%	90.9%	72.1%	97.7%	88.9%	85.4%	91.0%
38.3	80.0%	51.9%	94.1%	36.3%	24.6%	51.5%	37.0%	25.4%	42.3%	79.7%	58.0%	91.1%	37.8%	23.1%	54.2%	90.9%	72.0%	97.7%	88.9%	85.4%	91.0%
38.4	79.9%	51.8%	94.1%	36.3%	24.6%	51.4%	37.0%	25.3%	42.3%	79.6%	57.8%	91.1%	37.8%	23.1%	54.2%	90.9%	72.0%	97.6%	88.9%	85.3%	91.0%
38.5	79.8%	51.6%	94.0%	36.2%	24.6%	51.4%	37.0%	25.2%	42.3%	79.6%	57.7%	91.0%	37.8%	23.1%	54.2%	90.8%	71.9%	97.6%	88.9%	85.3%	91.0%
38.6	79.7%	51.5%	94.0%	36.2%	24.6%	51.4%	37.0%	25.1%	42.3%	79.5%	57.6%	90.9%	37.8%	23.1%	54.2%	90.8%	71.9%	97.6%	88.9%	85.3%	91.0%
38.7	79.7%	51.4%	94.0%	36.2%	24.5%	51.4%	37.0%	24.9%	42.3%	79.4%	57.5%	90.9%	37.8%	23.1%	54.2%	90.7%	71.8%	97.6%	88.9%	85.3%	91.0%
38.8	79.6%	51.3%	94.0%	36.2%	24.5%	51.4%	37.0%	24.8%	42.2%	79.3%	57.4%	90.8%	37.8%	23.1%	54.2%	90.7%	71.7%	97.6%	88.9%	85.3%	91.0%
38.9	79.5%	51.1%	93.9%	36.2%	24.5%	51.4%	37.0%	24.7%	42.2%	79.3%	57.3%	90.8%	37.8%	23.0%	54.2%	90.7%	71.7%	97.6%	88.9%	85.2%	91.0%
39	79.4%	51.0%	93.9%	36.2%	24.5%	51.4%	37.0%	24.6%	42.2%	79.2%	57.2%	90.8%	37.8%	23.0%	54.2%	90.6%	71.6%	97.6%	88.9%	85.2%	91.0%
39.1	79.3%	50.9%	93.9%	36.2%	24.5%	51.4%	37.0%	24.5%	42.2%	79.1%	57.1%	90.7%	37.8%	23.0%	54.2%	90.6%	71.4%	97.6%	88.9%	85.1%	91.0%
39.2	79.3%	50.8%	93.9%	36.2%	24.5%	51.3%	37.0%	24.4%	42.2%	79.0%	57.0%	90.7%	37.8%	23.0%	54.2%	90.5%	71.1%	97.6%	88.9%	85.1%	91.0%
39.3	79.2%	50.7%	93.8%	36.2%	24.5%	51.3%	37.0%	24.3%	42.2%	78.9%	56.9%	90.7%	37.8%	22.9%	54.2%	90.5%	70.9%	97.6%	88.9%	85.0%	91.0%
39.4	79.1%	50.5%	93.8%	36.1%	24.4%	51.3%	37.0%	24.2%	42.2%	78.9%	56.8%	90.7%	37.8%	22.9%	54.2%	90.4%	70.7%	97.6%	88.9%	84.9%	91.0%
39.5	79.0%	50.4%	93.8%	36.1%	24.4%	51.3%	37.0%	24.1%	42.2%	78.8%	56.7%	90.6%	37.8%	22.9%	54.2%	90.4%	70.5%	97.5%	88.9%	84.9%	91.0%
39.6	78.9%	50.3%	93.7%	36.1%	24.4%	51.3%	37.0%	24.0%	42.2%	78.7%	56.6%	90.6%	37.7%	22.8%	54.2%	90.4%	70.3%	97.5%	88.9%	84.9%	91.0%
39.7	78.9%	50.2%	93.7%	36.1%	24.4%	51.3%	37.0%	23.9%	42.2%	78.6%	56.5%	90.6%	37.7%	22.8%	54.2%	90.3%	70.1%	97.5%	88.9%	84.8%	90.9%
39.8	78.8%	50.0%	93.7%	36.1%	24.4%	51.3%	37.0%	23.7%	42.2%	78.6%	56.3%	90.5%	37.7%	22.8%	54.1%	90.3%	69.9%	97.5%	88.9%	84.8%	90.9%
39.9	78.7%	49.9%	93.7%	36.1%	24.4%	51.3%	37.0%	23.6%	42.2%	78.5%	56.2%	90.5%	37.7%	22.8%	54.1%	90.3%	69.7%	97.5%	88.9%	84.7%	90.9%
40	78.6%	49.8%	93.6%	36.1%	24.4%	51.3%	37.0%	23.5%	42.2%	78.4%	56.1%	90.5%	37.7%	22.8%	54.1%	90.2%	69.7%	97.5%	88.9%	84.7%	90.9%
40.1	78.5%	49.6%	93.6%	36.2%	24.4%	51.3%	37.0%	23.4%	42.2%	78.4%	55.9%	90.4%	37.7%	22.8%	54.1%	90.2%	69.7%	97.5%	88.9%	84.6%	90.9%
40.2	78.4%	49.5%	93.6%	36.2%	24.4%	51.3%	37.0%	23.3%	42.2%	78.3%	55.8%	90.4%	37.7%	22.7%	54.1%	90.1%	69.7%	97.5%	88.9%	84.6%	90.9%
40.3	78.3%	49.4%	93.5%	36.2%	24.4%	51.4%	37.0%	23.2%	42.2%	78.2%	55.7%	90.4%	37.7%	22.7%	54.0%	90.1%	69.7%	97.4%	88.9%	84.6%	90.9%
40.4	78.3%	49.2%	93.5%	36.2%	24.4%	51.4%	37.0%	23.1%	42.2%	78.1%	55.6%	90.3%	37.7%	22.7%	54.0%	90.1%	69.6%	97.4%	88.9%	84.5%	90.9%
40.5	78.2%	49.1%	93.5%	36.2%	24.4%	51.4%	37.0%	22.9%	42.2%	78.1%	55.4%	90.3%	37.6%	22.7%	54.0%	90.0%	69.4%	97.4%	88.9%	84.4%	90.9%
40.6	78.1%	49.0%	93.4%	36.2%	24.4%	51.4%	37.0%	22.8%	42.2%	78.0%	55.3%	90.3%	37.6%	22.6%	54.0%	90.0%	69.4%	97.4%	88.9%	84.3%	90.9%
40.7	78.0%	48.9%	93.4%	36.2%	24.4%	51.4%	37.0%	22.7%	42.2%	77.9%	55.2%	90.2%	37.6%	22.6%	54.0%	90.0%	69.3%	97.4%	88.9%	84.2%	90.9%
40.8	77.9%	48.7%	93.4%	36.2%	24.4%	51.4%	37.0%	22.6%	42.2%	77.9%	55.1%	90.2%	37.6%	22.6%	53.9%	89.9%	69.3%	97.4%	88.9%	84.2%	90.9%
40.9	77.8%	48.6%	93.3%	36.2%	24.4%	51.4%	37.0%	22.6%	42.2%	77.8%	54.9%	90.1%	37.6%	22.6%	53.9%	89.9%	69.2%	97.4%	88.9%	84.0%	90.9%

41	77.7%	48.5%	93.3%	36.2%	24.4%	51.4%	37.0%	22.5%	42.2%	77.7%	54.8%	90.1%	37.6%	22.5%	53.9%	89.9%	69.2%	97.4%	88.9%	84.0%	90.9%
41.1	77.6%	48.3%	93.3%	36.2%	24.4%	51.3%	37.0%	22.4%	42.2%	77.6%	54.7%	90.1%	37.5%	22.5%	53.9%	89.8%	69.1%	97.4%	88.9%	83.9%	90.9%
41.2	77.5%	48.2%	93.2%	36.2%	24.4%	51.3%	37.0%	22.4%	42.2%	77.6%	54.6%	90.0%	37.5%	22.5%	53.8%	89.8%	69.1%	97.4%	88.9%	83.8%	90.9%
41.3	77.4%	48.0%	93.2%	36.2%	24.4%	51.3%	37.0%	22.3%	42.2%	77.5%	54.5%	90.0%	37.5%	22.4%	53.8%	89.8%	69.0%	97.4%	88.9%	83.7%	90.9%
41.4	77.3%	47.9%	93.2%	36.2%	24.4%	51.3%	37.0%	22.3%	42.2%	77.4%	54.4%	90.0%	37.5%	22.4%	53.8%	89.7%	68.9%	97.4%	88.9%	83.5%	90.8%
41.5	77.3%	47.8%	93.2%	36.2%	24.4%	51.3%	37.0%	22.2%	42.2%	77.4%	54.3%	89.9%	37.5%	22.4%	53.8%	89.7%	68.6%	97.4%	88.9%	83.5%	90.8%
41.6	77.2%	47.6%	93.1%	36.3%	24.4%	51.3%	37.0%	22.2%	42.2%	77.3%	54.2%	89.9%	37.4%	22.3%	53.7%	89.7%	68.6%	97.4%	88.9%	83.4%	90.8%
41.7	77.1%	47.5%	93.1%	36.3%	24.4%	51.3%	37.0%	22.1%	42.2%	77.2%	54.1%	89.9%	37.4%	22.3%	53.7%	89.6%	68.6%	97.3%	88.9%	83.3%	90.8%
41.8	77.0%	47.4%	93.1%	36.3%	24.4%	51.3%	37.0%	22.1%	42.2%	77.2%	54.0%	89.8%	37.4%	22.3%	53.7%	89.6%	68.5%	97.3%	88.9%	83.1%	90.8%
41.9	76.9%	47.2%	93.0%	36.3%	24.4%	51.3%	37.0%	22.0%	42.2%	77.1%	53.9%	89.8%	37.3%	22.3%	53.7%	89.6%	68.5%	97.3%	88.8%	83.0%	90.8%
42	76.8%	47.1%	93.0%	36.3%	24.4%	51.3%	37.0%	22.0%	42.2%	77.1%	53.9%	89.8%	37.3%	22.2%	53.6%	89.6%	68.5%	97.2%	88.8%	82.9%	90.8%
42.1	76.7%	46.9%	92.9%	36.3%	24.4%	51.3%	37.0%	22.0%	42.2%	77.0%	53.8%	89.7%	37.3%	22.2%	53.6%	89.5%	68.4%	97.2%	88.8%	82.8%	90.8%
42.2	76.6%	46.8%	92.9%	36.3%	24.4%	51.3%	37.0%	21.9%	42.2%	76.9%	53.7%	89.7%	37.3%	22.2%	53.5%	89.5%	68.4%	97.2%	88.8%	82.7%	90.8%
42.3	76.5%	46.7%	92.9%	36.3%	24.4%	51.3%	37.0%	21.9%	42.2%	76.9%	53.6%	89.7%	37.2%	22.1%	53.5%	89.5%	68.3%	97.2%	88.8%	82.6%	90.8%
42.4	76.4%	46.5%	92.8%	36.3%	24.4%	51.3%	37.0%	21.9%	42.2%	76.8%	53.5%	89.6%	37.2%	22.1%	53.4%	89.4%	68.3%	97.2%	88.8%	82.5%	90.8%
42.5	76.3%	46.4%	92.8%	36.3%	24.4%	51.3%	37.0%	21.9%	42.2%	76.7%	53.4%	89.6%	37.2%	22.1%	53.4%	89.4%	68.2%	97.2%	88.8%	82.4%	90.7%
42.6	76.2%	46.2%	92.8%	36.3%	24.4%	51.3%	37.0%	21.9%	42.2%	76.7%	53.3%	89.6%	37.1%	22.0%	53.3%	89.4%	68.1%	97.2%	88.8%	82.3%	90.7%
42.7	76.1%	46.1%	92.7%	36.3%	24.4%	51.3%	37.0%	21.8%	42.2%	76.6%	53.3%	89.5%	37.1%	22.0%	53.3%	89.4%	68.0%	97.2%	88.7%	82.2%	90.7%
42.8	76.0%	45.9%	92.7%	36.3%	24.4%	51.3%	37.0%	21.8%	42.2%	76.6%	53.2%	89.5%	37.1%	22.0%	53.2%	89.3%	67.9%	97.2%	88.7%	82.1%	90.7%
42.9	75.9%	45.8%	92.7%	36.3%	24.4%	51.3%	37.0%	21.8%	42.2%	76.5%	53.1%	89.5%	37.0%	21.9%	53.2%	89.3%	67.7%	97.2%	88.7%	82.1%	90.7%
43	75.8%	45.7%	92.6%	36.3%	24.4%	51.3%	37.0%	21.8%	42.2%	76.4%	53.0%	89.4%	37.0%	21.9%	53.1%	89.3%	67.6%	97.1%	88.7%	82.0%	90.6%
43.1	75.7%	45.5%	92.6%	36.3%	24.4%	51.3%	37.0%	21.8%	42.2%	76.4%	52.9%	89.4%	36.9%	21.9%	53.1%	89.3%	67.5%	97.1%	88.7%	81.9%	90.6%
43.2	75.6%	45.4%	92.6%	36.3%	24.4%	51.3%	37.0%	21.8%	42.2%	76.3%	52.8%	89.4%	36.9%	21.8%	53.0%	89.2%	67.2%	97.1%	88.7%	81.8%	90.6%
43.3	75.5%	45.2%	92.5%	36.3%	24.4%	51.3%	37.0%	21.8%	42.2%	76.3%	52.7%	89.3%	36.9%	21.8%	53.0%	89.2%	66.9%	97.1%	88.6%	81.7%	90.6%
43.4	75.4%	45.1%	92.5%	36.3%	24.4%	51.3%	37.0%	21.8%	42.2%	76.2%	52.6%	89.3%	36.8%	21.7%	52.9%	89.2%	66.7%	97.1%	88.6%	81.6%	90.6%
43.5	75.3%	44.9%	92.4%	36.3%	24.4%	51.3%	37.0%	21.8%	42.2%	76.1%	52.5%	89.3%	36.8%	21.7%	52.9%	89.2%	66.6%	97.1%	88.6%	81.5%	90.5%
43.6	75.2%	44.8%	92.4%	36.3%	24.4%	51.3%	37.0%	21.8%	42.2%	76.1%	52.4%	89.3%	36.7%	21.7%	52.8%	89.1%	66.6%	97.1%	88.6%	81.4%	90.5%
43.7	75.1%	44.6%	92.4%	36.3%	24.4%	51.3%	37.0%	21.8%	42.2%	76.0%	52.3%	89.2%	36.7%	21.7%	52.8%	89.1%	66.5%	97.1%	88.6%	81.4%	90.5%
43.8	75.0%	44.5%	92.3%	36.3%	24.4%	51.3%	37.0%	21.8%	42.2%	76.0%	52.3%	89.2%	36.6%	21.6%	52.7%	89.1%	66.5%	97.1%	88.5%	81.3%	90.4%
43.9	74.8%	44.3%	92.3%	36.3%	24.4%	51.3%	37.0%	21.8%	42.2%	75.9%	52.2%	89.2%	36.6%	21.6%	52.7%	89.1%	66.2%	97.1%	88.5%	81.2%	90.4%
44	74.7%	44.2%	92.2%	36.3%	24.4%	51.3%	37.0%	21.8%	42.2%	75.9%	52.2%	89.1%	36.6%	21.5%	52.6%	89.1%	66.0%	97.1%	88.5%	81.1%	90.4%
44.1	74.6%	44.1%	92.2%	36.3%	24.4%	51.3%	37.0%	21.8%	42.2%	75.8%	52.1%	89.1%	36.5%	21.5%	52.5%	89.0%	66.0%	97.1%	88.5%	81.0%	90.4%

44.2	74.5%	44.0%	92.2%	36.3%	24.4%	51.2%	37.0%	21.8%	42.2%	75.8%	52.1%	89.1%	36.5%	21.5%	52.5%	89.0%	65.9%	97.1%	88.4%	81.0%	90.3%
44.3	74.4%	43.8%	92.1%	36.3%	24.4%	51.2%	37.0%	21.8%	42.2%	75.7%	52.0%	89.1%	36.4%	21.4%	52.4%	89.0%	65.9%	97.1%	88.4%	80.9%	90.3%
44.4	74.3%	43.7%	92.1%	36.3%	24.4%	51.2%	37.0%	21.8%	42.2%	75.7%	51.9%	89.0%	36.4%	21.4%	52.4%	89.0%	65.8%	97.1%	88.4%	80.8%	90.3%
44.5	74.2%	43.5%	92.0%	36.3%	24.4%	51.2%	37.0%	21.7%	42.2%	75.6%	51.8%	89.0%	36.3%	21.4%	52.3%	89.0%	65.8%	97.1%	88.4%	80.7%	90.2%
44.6	74.1%	43.4%	92.0%	36.3%	24.4%	51.2%	37.0%	21.7%	42.2%	75.6%	51.8%	89.0%	36.3%	21.3%	52.2%	89.0%	65.7%	97.1%	88.3%	80.6%	90.2%
44.7	74.0%	43.2%	91.9%	36.3%	24.4%	51.2%	37.0%	21.7%	42.1%	75.5%	51.7%	89.0%	36.2%	21.3%	52.2%	88.9%	65.7%	97.1%	88.3%	80.5%	90.2%
44.8	73.9%	43.1%	91.9%	36.3%	24.4%	51.2%	37.0%	21.6%	42.1%	75.5%	51.6%	88.9%	36.2%	21.2%	52.1%	88.9%	65.5%	97.1%	88.3%	80.5%	90.2%
44.9	73.7%	42.9%	91.8%	36.3%	24.4%	51.2%	37.0%	21.6%	42.1%	75.4%	51.6%	88.9%	36.1%	21.2%	52.1%	88.9%	65.1%	97.1%	88.3%	80.4%	90.1%
45	73.6%	42.8%	91.8%	36.3%	24.4%	51.2%	37.0%	21.6%	42.1%	75.4%	51.5%	88.9%	36.0%	21.2%	52.0%	88.9%	65.1%	97.1%	88.2%	80.3%	90.1%
45.1	73.5%	42.6%	91.7%	36.3%	24.4%	51.2%	37.0%	21.6%	42.1%	75.3%	51.4%	88.9%	36.0%	21.1%	51.9%	88.9%	65.1%	97.1%	88.2%	80.2%	90.1%
45.2	73.4%	42.5%	91.7%	36.3%	24.4%	51.1%	37.0%	21.6%	42.1%	75.3%	51.4%	88.8%	35.9%	21.1%	51.9%	88.9%	64.6%	97.1%	88.2%	80.1%	90.0%
45.3	73.3%	42.3%	91.6%	36.3%	24.4%	51.1%	37.0%	21.6%	42.1%	75.2%	51.3%	88.8%	35.9%	21.0%	51.8%	88.9%	64.0%	97.1%	88.1%	80.0%	90.0%
45.4	73.2%	42.2%	91.6%	36.3%	24.4%	51.1%	37.0%	21.6%	42.1%	75.2%	51.2%	88.8%	35.8%	21.0%	51.8%	88.8%	63.5%	97.0%	88.1%	80.0%	90.0%
45.5	73.0%	42.0%	91.5%	36.3%	24.4%	51.1%	37.0%	21.6%	42.1%	75.1%	51.2%	88.8%	35.8%	21.0%	51.7%	88.8%	62.9%	97.0%	88.1%	79.9%	89.9%
45.6	72.9%	41.9%	91.5%	36.3%	24.4%	51.1%	37.0%	21.6%	42.1%	75.1%	51.1%	88.7%	35.7%	20.9%	51.6%	88.8%	62.3%	97.0%	88.0%	79.8%	89.9%
45.7	72.8%	41.7%	91.4%	36.3%	24.3%	51.1%	37.0%	21.5%	42.1%	75.1%	51.1%	88.7%	35.6%	20.9%	51.6%	88.8%	61.6%	97.0%	88.0%	79.7%	89.9%
45.8	72.7%	41.6%	91.3%	36.3%	24.3%	51.1%	37.0%	21.4%	42.1%	75.0%	51.0%	88.6%	35.6%	20.8%	51.5%	88.8%	61.5%	97.0%	88.0%	79.7%	89.8%
45.9	72.6%	41.5%	91.3%	36.3%	24.3%	51.0%	37.0%	21.3%	42.1%	75.0%	51.0%	88.6%	35.5%	20.8%	51.4%	88.8%	61.5%	97.0%	87.9%	79.6%	89.8%
46	72.5%	41.3%	91.2%	36.3%	24.3%	51.0%	37.0%	21.2%	42.1%	74.9%	50.9%	88.6%	35.4%	20.8%	51.4%	88.8%	61.4%	97.0%	87.9%	79.6%	89.8%
46.1	72.3%	41.2%	91.2%	36.3%	24.3%	51.0%	37.0%	21.2%	42.1%	74.9%	50.9%	88.6%	35.4%	20.7%	51.3%	88.8%	61.3%	97.0%	87.9%	79.5%	89.7%
46.2	72.2%	41.1%	91.1%	36.3%	24.3%	51.0%	37.0%	21.2%	42.1%	74.8%	50.8%	88.6%	35.3%	20.7%	51.2%	88.8%	61.1%	97.0%	87.8%	79.4%	89.7%
46.3	72.1%	41.0%	91.1%	36.2%	24.3%	51.0%	37.0%	21.2%	42.1%	74.8%	50.8%	88.6%	35.2%	20.6%	51.2%	88.8%	61.1%	97.0%	87.8%	79.4%	89.6%
46.4	72.0%	40.9%	91.0%	36.2%	24.3%	51.0%	37.0%	21.2%	42.0%	74.8%	50.7%	88.5%	35.2%	20.5%	51.1%	88.7%	61.0%	97.0%	87.8%	79.3%	89.6%
46.5	71.8%	40.7%	91.0%	36.2%	24.3%	50.9%	37.0%	21.1%	42.0%	74.7%	50.7%	88.5%	35.1%	20.4%	51.0%	88.7%	60.9%	97.0%	87.7%	79.3%	89.6%
46.6	71.7%	40.6%	90.9%	36.2%	24.3%	50.9%	37.0%	21.0%	42.0%	74.7%	50.6%	88.5%	35.0%	20.4%	51.0%	88.7%	60.7%	97.0%	87.7%	79.2%	89.6%
46.7	71.6%	40.5%	90.8%	36.2%	24.3%	50.9%	37.0%	20.9%	42.0%	74.7%	50.6%	88.5%	35.0%	20.4%	50.9%	88.7%	60.5%	97.0%	87.6%	79.2%	89.6%
46.8	71.5%	40.4%	90.8%	36.2%	24.3%	50.9%	37.0%	20.9%	42.0%	74.6%	50.5%	88.5%	34.9%	20.3%	50.8%	88.7%	59.8%	97.0%	87.6%	79.1%	89.5%
46.9	71.4%	40.3%	90.7%	36.2%	24.3%	50.9%	37.0%	20.9%	42.0%	74.6%	50.5%	88.5%	34.8%	20.3%	50.8%	88.7%	59.5%	97.0%	87.6%	79.1%	89.5%
47	71.2%	40.2%	90.7%	36.2%	24.2%	50.9%	37.0%	20.9%	42.0%	74.5%	50.4%	88.4%	34.8%	20.3%	50.7%	88.7%	59.4%	96.9%	87.5%	79.1%	89.4%
47.1	71.1%	40.0%	90.6%	36.2%	24.2%	50.9%	37.0%	20.9%	42.0%	74.5%	50.4%	88.4%	34.7%	20.2%	50.6%	88.7%	58.8%	96.9%	87.5%	79.0%	89.4%
47.2	71.0%	39.9%	90.6%	36.2%	24.2%	50.9%	37.0%	20.9%	42.0%	74.5%	50.4%	88.4%	34.6%	20.2%	50.5%	88.7%	58.3%	96.9%	87.4%	79.0%	89.3%
47.3	70.9%	39.8%	90.5%	36.2%	24.2%	50.9%	37.0%	20.9%	42.0%	74.4%	50.3%	88.4%	34.5%	20.1%	50.5%	88.7%	58.1%	96.9%	87.4%	79.0%	89.3%

47.4	70.8%	39.7%	90.5%	36.2%	24.2%	50.9%	37.0%	20.9%	42.0%	74.4%	50.3%	88.4%	34.5%	20.1%	50.4%	88.7%	57.9%	96.9%	87.3%	78.9%	89.3%
47.5	70.6%	39.6%	90.4%	36.2%	24.2%	50.9%	37.0%	20.9%	42.0%	74.4%	50.2%	88.3%	34.4%	20.1%	50.3%	88.7%	57.4%	96.9%	87.3%	78.9%	89.2%
47.6	70.5%	39.5%	90.4%	36.2%	24.2%	50.9%	37.0%	20.9%	42.0%	74.3%	50.2%	88.3%	34.3%	20.0%	50.2%	88.7%	56.8%	96.9%	87.2%	78.9%	89.2%
47.7	70.4%	39.3%	90.3%	36.1%	24.2%	50.9%	37.0%	20.8%	42.0%	74.3%	50.1%	88.3%	34.2%	20.0%	50.1%	88.7%	56.6%	96.9%	87.2%	78.9%	89.1%
47.8	70.3%	39.2%	90.3%	36.1%	24.2%	50.9%	37.0%	20.8%	42.0%	74.2%	50.1%	88.3%	34.2%	20.0%	50.0%	88.7%	55.9%	96.9%	87.1%	78.8%	89.1%
47.9	70.2%	39.1%	90.2%	36.1%	24.2%	50.9%	37.0%	20.8%	42.0%	74.2%	50.0%	88.3%	34.1%	19.9%	49.9%	88.7%	55.5%	96.9%	87.1%	78.8%	89.0%
48	70.1%	39.0%	90.2%	36.1%	24.2%	50.9%	37.0%	20.8%	42.0%	74.2%	50.0%	88.2%	34.0%	19.9%	49.8%	88.7%	55.1%	96.9%	87.0%	78.8%	89.0%
48.1	69.9%	38.9%	90.1%	36.1%	24.2%	50.9%	37.0%	20.8%	42.0%	74.1%	49.9%	88.2%	33.9%	19.8%	49.8%	88.7%	54.7%	96.9%	87.0%	78.7%	88.9%
48.2	69.8%	38.8%	90.1%	36.1%	24.2%	50.9%	37.0%	20.8%	42.0%	74.1%	49.9%	88.2%	33.8%	19.8%	49.7%	88.7%	53.9%	96.9%	86.9%	78.7%	88.9%
48.3	69.7%	38.6%	90.0%	36.1%	24.2%	50.9%	37.0%	20.8%	42.0%	74.1%	49.8%	88.2%	33.8%	19.8%	49.6%	88.7%	52.9%	96.8%	86.9%	78.7%	88.8%
48.4	69.6%	38.5%	90.0%	36.1%	24.2%	50.9%	37.0%	20.8%	42.0%	74.0%	49.8%	88.2%	33.7%	19.7%	49.5%	88.7%	52.0%	96.8%	86.8%	78.7%	88.8%
48.5	69.5%	38.4%	89.9%	36.1%	24.2%	50.9%	37.0%	20.8%	42.0%	74.0%	49.7%	88.1%	33.6%	19.7%	49.5%	88.7%	51.0%	96.8%	86.8%	78.7%	88.7%
48.6	69.3%	38.3%	89.9%	36.1%	24.1%	50.9%	37.0%	20.8%	42.0%	74.0%	49.6%	88.1%	33.5%	19.6%	49.4%	88.7%	50.3%	96.8%	86.7%	78.7%	88.7%
48.7	69.2%	38.2%	89.8%	36.1%	24.1%	50.9%	37.0%	20.8%	42.0%	73.9%	49.5%	88.1%	33.5%	19.6%	49.3%	88.7%	50.3%	96.8%	86.7%	78.7%	88.6%
48.8	69.1%	38.0%	89.8%	36.0%	24.1%	50.9%	37.0%	20.8%	42.0%	73.9%	49.5%	88.1%	33.4%	19.5%	49.2%	88.7%	50.2%	96.7%	86.6%	78.6%	88.5%
48.9	69.0%	37.9%	89.8%	36.0%	24.1%	50.9%	37.0%	20.8%	42.0%	73.8%	49.4%	88.1%	33.3%	19.5%	49.2%	88.7%	50.2%	96.7%	86.6%	78.6%	88.5%
49	68.9%	37.8%	89.7%	36.0%	24.1%	50.9%	37.0%	20.8%	42.0%	73.8%	49.3%	88.0%	33.2%	19.4%	49.1%	88.7%	49.8%	96.7%	86.5%	78.6%	88.4%
49.1	68.8%	37.6%	89.7%	36.0%	24.1%	50.9%	37.0%	20.8%	42.0%	73.8%	49.2%	88.0%	33.2%	19.4%	49.0%	88.7%	49.1%	96.7%	86.4%	78.6%	88.4%
49.2	68.6%	37.5%	89.6%	36.0%	24.1%	50.9%	37.0%	20.8%	42.0%	73.7%	49.1%	88.0%	33.1%	19.4%	48.9%	88.7%	48.5%	96.6%	86.4%	78.6%	88.3%
49.3	68.5%	37.4%	89.6%	36.0%	24.1%	50.9%	37.0%	20.8%	42.0%	73.7%	49.0%	88.0%	33.0%	19.3%	48.8%	88.7%	48.3%	96.6%	86.3%	78.6%	88.2%
49.4	68.4%	37.3%	89.5%	36.0%	24.1%	50.9%	37.0%	20.8%	42.0%	73.7%	48.9%	87.9%	32.9%	19.3%	48.8%	88.7%	48.3%	96.6%	86.3%	78.6%	88.2%
49.5	68.3%	37.1%	89.5%	36.0%	24.1%	50.9%	37.0%	20.8%	42.0%	73.6%	48.9%	87.9%	32.9%	19.2%	48.7%	88.7%	47.9%	96.6%	86.2%	78.6%	88.1%
49.6	68.2%	37.0%	89.4%	35.9%	24.1%	50.8%	37.0%	20.7%	42.0%	73.6%	48.8%	87.9%	32.8%	19.2%	48.6%	88.7%	47.9%	96.6%	86.1%	78.6%	88.1%
49.7	68.1%	36.9%	89.4%	35.9%	24.0%	50.8%	37.0%	20.7%	42.0%	73.5%	48.7%	87.9%	32.7%	19.1%	48.5%	88.7%	47.7%	96.6%	86.1%	78.6%	88.0%
49.8	67.9%	36.8%	89.3%	35.9%	24.0%	50.8%	37.0%	20.7%	42.0%	73.5%	48.6%	87.9%	32.6%	19.1%	48.5%	88.7%	47.0%	96.6%	86.0%	78.6%	88.0%
49.9	67.8%	36.6%	89.3%	35.9%	24.0%	50.8%	37.0%	20.7%	42.0%	73.5%	48.5%	87.9%	32.6%	19.0%	48.4%	88.7%	46.3%	96.6%	85.9%	78.5%	87.9%
50	67.7%	36.5%	89.2%	35.9%	24.0%	50.8%	37.0%	20.7%	42.0%	73.4%	48.4%	87.8%	32.5%	18.9%	48.3%	88.7%	45.7%	96.6%	85.9%	78.5%	87.9%
50.1	67.6%	36.4%	89.2%	35.9%	23.9%	50.7%	37.0%	20.7%	42.0%	73.4%	48.3%	87.8%	32.4%	18.9%	48.2%	88.7%	45.0%	96.6%	85.8%	78.5%	87.8%
50.2	67.5%	36.3%	89.1%	35.9%	23.9%	50.7%	37.0%	20.7%	42.0%	73.4%	48.3%	87.8%	32.3%	18.8%	48.1%	88.7%	44.5%	96.6%	85.8%	78.5%	87.8%
50.3	67.4%	36.2%	89.1%	35.9%	23.9%	50.7%	37.0%	20.7%	42.0%	73.3%	48.2%	87.8%	32.3%	18.8%	48.1%	88.7%	43.6%	96.6%	85.7%	78.5%	87.7%
50.4	67.2%	36.0%	89.0%	35.8%	23.9%	50.7%	37.0%	20.7%	42.0%	73.3%	48.1%	87.8%	32.2%	18.8%	48.0%	88.7%	43.0%	96.5%	85.6%	78.5%	87.6%
50.5	67.1%	35.9%	89.0%	35.8%	23.8%	50.7%	37.0%	20.7%	42.0%	73.2%	48.0%	87.7%	32.1%	18.8%	47.9%	88.7%	42.3%	96.5%	85.6%	78.5%	87.6%

50.6	67.0%	35.8%	88.9%	35.8%	23.8%	50.6%	37.0%	20.7%	42.0%	73.2%	47.9%	87.7%	32.0%	18.7%	47.8%	88.7%	41.6%	96.5%	85.5%	78.4%	87.6%
50.7	66.9%	35.7%	88.9%	35.8%	23.8%	50.6%	37.0%	20.7%	42.0%	73.2%	47.9%	87.6%	32.0%	18.7%	47.8%	88.7%	41.0%	96.5%	85.5%	78.4%	87.5%
50.8	66.8%	35.5%	88.8%	35.8%	23.8%	50.6%	37.0%	20.7%	42.0%	73.1%	47.8%	87.6%	31.9%	18.6%	47.7%	88.7%	40.5%	96.5%	85.4%	78.4%	87.5%
50.9	66.7%	35.4%	88.8%	35.8%	23.7%	50.6%	37.0%	20.7%	41.9%	73.1%	47.7%	87.6%	31.8%	18.6%	47.6%	88.7%	40.2%	96.5%	85.3%	78.4%	87.4%
51	66.6%	35.3%	88.7%	35.7%	23.7%	50.6%	37.0%	20.7%	41.9%	73.1%	47.7%	87.5%	31.7%	18.5%	47.5%	88.7%	39.8%	96.5%	85.3%	78.4%	87.3%
51.1	66.5%	35.2%	88.7%	35.7%	23.7%	50.5%	37.0%	20.7%	41.9%	73.0%	47.6%	87.5%	31.7%	18.5%	47.4%	88.7%	39.5%	96.5%	85.2%	78.4%	87.3%
51.2	66.3%	35.1%	88.6%	35.7%	23.7%	50.5%	37.0%	20.7%	41.9%	73.0%	47.5%	87.5%	31.6%	18.4%	47.4%	88.7%	39.0%	96.5%	85.1%	78.4%	87.2%
51.3	66.2%	35.0%	88.6%	35.7%	23.6%	50.5%	37.0%	20.7%	41.9%	72.9%	47.5%	87.4%	31.5%	18.4%	47.3%	88.7%	38.6%	96.5%	85.1%	78.4%	87.2%
51.4	66.1%	34.9%	88.5%	35.7%	23.6%	50.5%	37.0%	20.7%	41.9%	72.9%	47.4%	87.4%	31.4%	18.3%	47.2%	88.7%	37.9%	96.5%	85.0%	78.4%	87.1%
51.5	66.0%	34.7%	88.5%	35.7%	23.6%	50.4%	37.0%	20.7%	41.9%	72.9%	47.4%	87.3%	31.4%	18.3%	47.1%	88.7%	37.3%	96.5%	84.9%	78.4%	87.1%
51.6	65.9%	34.6%	88.4%	35.6%	23.5%	50.4%	37.0%	20.7%	41.9%	72.8%	47.3%	87.3%	31.3%	18.2%	47.1%	88.7%	36.7%	96.5%	84.9%	78.3%	87.0%
51.7	65.8%	34.5%	88.4%	35.6%	23.5%	50.4%	37.0%	20.7%	41.9%	72.8%	47.2%	87.3%	31.2%	18.1%	47.0%	88.7%	36.1%	96.5%	84.8%	78.3%	87.0%
51.8	65.7%	34.4%	88.3%	35.6%	23.5%	50.4%	37.0%	20.7%	41.9%	72.8%	47.2%	87.2%	31.1%	18.1%	46.9%	88.7%	35.4%	96.5%	84.7%	78.3%	86.9%
51.9	65.6%	34.3%	88.3%	35.6%	23.4%	50.3%	37.0%	20.7%	41.9%	72.7%	47.2%	87.2%	31.1%	18.0%	46.8%	88.7%	34.8%	96.5%	84.7%	78.2%	86.9%
52	65.4%	34.2%	88.2%	35.6%	23.4%	50.3%	37.0%	20.7%	41.9%	72.7%	47.2%	87.2%	31.0%	17.9%	46.7%	88.7%	34.2%	96.5%	84.6%	78.2%	86.9%
52.1	65.3%	34.1%	88.2%	35.6%	23.4%	50.3%	37.0%	20.7%	41.9%	72.6%	47.2%	87.2%	30.9%	17.9%	46.7%	88.7%	33.5%	96.5%	84.5%	78.1%	86.8%
52.2	65.2%	34.0%	88.1%	35.5%	23.4%	50.3%	37.0%	20.7%	41.9%	72.6%	47.1%	87.1%	30.8%	17.8%	46.6%	88.7%	32.6%	96.5%	84.5%	78.1%	86.8%
52.3	65.1%	33.9%	88.1%	35.5%	23.4%	50.2%	37.0%	20.7%	41.9%	72.6%	47.1%	87.1%	30.8%	17.7%	46.5%	88.7%	31.7%	96.5%	84.4%	78.1%	86.8%
52.4	65.0%	33.8%	88.0%	35.5%	23.4%	50.2%	37.0%	20.7%	41.9%	72.5%	47.1%	87.1%	30.7%	17.6%	46.4%	88.7%	30.9%	96.4%	84.3%	78.1%	86.7%
52.5	64.9%	33.7%	88.0%	35.5%	23.4%	50.2%	37.0%	20.7%	41.9%	72.5%	47.1%	87.1%	30.6%	17.6%	46.4%	88.7%	30.5%	96.4%	84.3%	78.1%	86.7%
52.6	64.8%	33.6%	87.9%	35.5%	23.4%	50.2%	37.0%	20.7%	41.9%	72.4%	47.1%	87.1%	30.6%	17.5%	46.3%	88.7%	29.8%	96.4%	84.2%	78.1%	86.7%
52.7	64.7%	33.5%	87.9%	35.5%	23.4%	50.2%	37.0%	20.7%	41.9%	72.4%	47.1%	87.0%	30.5%	17.4%	46.2%	88.7%	29.1%	96.4%	84.1%	78.1%	86.6%
52.8	64.6%	33.4%	87.8%	35.4%	23.4%	50.1%	37.0%	20.7%	41.9%	72.4%	47.0%	87.0%	30.4%	17.4%	46.1%	88.7%	28.6%	96.4%	84.1%	78.1%	86.6%
52.9	64.5%	33.3%	87.8%	35.4%	23.4%	50.1%	37.0%	20.7%	41.9%	72.3%	47.0%	87.0%	30.3%	17.4%	46.1%	88.7%	28.2%	96.4%	84.0%	78.0%	86.5%
53	64.4%	33.2%	87.7%	35.4%	23.4%	50.1%	37.0%	20.7%	41.9%	72.3%	47.0%	87.0%	30.3%	17.3%	46.0%	88.7%	27.7%	96.4%	83.9%	78.0%	86.5%
53.1	64.3%	33.1%	87.7%	35.4%	23.4%	50.1%	37.0%	20.7%	41.9%	72.3%	47.0%	86.9%	30.2%	17.3%	45.9%	88.7%	27.1%	96.4%	83.9%	78.0%	86.5%
53.2	64.1%	33.0%	87.6%	35.4%	23.4%	50.1%	37.0%	20.7%	41.9%	72.2%	47.0%	86.9%	30.1%	17.2%	45.8%	88.7%	26.5%	96.4%	83.8%	78.0%	86.4%
53.3	64.0%	32.9%	87.6%	35.4%	23.4%	50.0%	37.0%	20.7%	41.9%	72.2%	47.0%	86.9%	30.0%	17.2%	45.7%	88.7%	25.9%	96.4%	83.7%	77.9%	86.4%
53.4	63.9%	32.8%	87.5%	35.4%	23.3%	50.0%	37.0%	20.7%	41.9%	72.1%	46.9%	86.9%	30.0%	17.1%	45.6%	88.7%	25.2%	96.4%	83.7%	77.9%	86.4%
53.5	63.8%	32.7%	87.5%	35.3%	23.3%	50.0%	37.0%	20.7%	41.9%	72.1%	46.9%	86.9%	29.9%	17.1%	45.5%	88.7%	24.6%	96.4%	83.6%	77.9%	86.3%
53.6	63.7%	32.6%	87.4%	35.3%	23.3%	50.0%	37.0%	20.7%	41.9%	72.1%	46.9%	86.9%	29.8%	17.0%	45.4%	88.7%	24.0%	96.4%	83.5%	77.9%	86.3%
53.7	63.6%	32.5%	87.4%	35.3%	23.3%	50.0%	37.0%	20.6%	41.9%	72.0%	46.9%	86.8%	29.8%	17.0%	45.3%	88.7%	23.4%	96.4%	83.5%	77.8%	86.2%

53.8	63.5%	32.4%	87.3%	35.3%	23.3%	49.9%	37.0%	20.5%	41.9%	72.0%	46.9%	86.8%	29.7%	16.9%	45.2%	88.7%	22.9%	96.4%	83.4%	77.8%	86.2%
53.9	63.4%	32.3%	87.3%	35.3%	23.3%	49.9%	37.0%	20.5%	41.9%	71.9%	46.9%	86.8%	29.6%	16.8%	45.2%	88.7%	22.4%	96.4%	83.3%	77.7%	86.2%
54	63.3%	32.2%	87.2%	35.3%	23.3%	49.9%	37.0%	20.5%	41.9%	71.9%	46.8%	86.8%	29.5%	16.8%	45.1%	88.7%	21.9%	96.4%	83.2%	77.6%	86.1%
54.1	63.2%	32.2%	87.2%	35.3%	23.3%	49.9%	37.0%	20.5%	41.9%	71.9%	46.8%	86.8%	29.5%	16.7%	45.0%	88.7%	21.5%	96.4%	83.2%	77.6%	86.1%
54.2	63.1%	32.1%	87.1%	35.3%	23.3%	49.9%	37.0%	20.5%	41.9%	71.8%	46.8%	86.8%	29.4%	16.7%	44.9%	88.7%	21.0%	96.4%	83.1%	77.6%	86.0%
54.3	63.0%	32.0%	87.1%	35.2%	23.3%	49.8%	37.0%	20.5%	41.9%	71.8%	46.8%	86.8%	29.3%	16.6%	44.8%	88.7%	20.5%	96.4%	83.0%	77.6%	86.0%
54.4	62.9%	31.9%	87.0%	35.2%	23.3%	49.8%	37.0%	20.5%	41.9%	71.8%	46.8%	86.7%	29.3%	16.6%	44.7%	88.7%	20.1%	96.4%	83.0%	77.6%	86.0%
54.5	62.8%	31.8%	87.0%	35.2%	23.3%	49.8%	37.0%	20.5%	41.9%	71.7%	46.8%	86.7%	29.2%	16.5%	44.6%	88.7%	19.7%	96.4%	82.9%	77.6%	85.9%
54.6	62.7%	31.7%	86.9%	35.2%	23.3%	49.8%	37.0%	20.4%	41.9%	71.7%	46.7%	86.7%	29.1%	16.4%	44.5%	88.7%	19.3%	96.4%	82.8%	77.5%	85.8%
54.7	62.6%	31.6%	86.9%	35.2%	23.3%	49.8%	37.0%	20.3%	41.9%	71.6%	46.7%	86.7%	29.0%	16.4%	44.4%	88.7%	18.8%	96.4%	82.7%	77.4%	85.8%
54.8	62.5%	31.5%	86.8%	35.2%	23.3%	49.8%	37.0%	20.3%	41.9%	71.6%	46.6%	86.7%	29.0%	16.3%	44.3%	88.7%	18.4%	96.4%	82.7%	77.4%	85.8%
54.9	62.4%	31.4%	86.8%	35.2%	23.3%	49.7%	37.0%	20.3%	41.9%	71.6%	46.6%	86.7%	28.9%	16.3%	44.3%	88.7%	18.0%	96.4%	82.6%	77.3%	85.8%
55	62.3%	31.3%	86.7%	35.2%	23.2%	49.7%	37.0%	20.3%	41.9%	71.5%	46.5%	86.6%	28.8%	16.2%	44.2%	88.7%	17.6%	96.4%	82.5%	77.3%	85.7%
55.1	62.2%	31.2%	86.7%	35.1%	23.2%	49.7%	37.0%	20.3%	41.9%	71.5%	46.4%	86.6%	28.8%	16.2%	44.1%	88.7%	17.2%	96.4%	82.5%	77.2%	85.7%
55.2	62.1%	31.1%	86.7%	35.1%	23.2%	49.7%	37.0%	20.3%	41.9%	71.4%	46.3%	86.5%	28.7%	16.1%	44.0%	88.7%	16.8%	96.4%	82.4%	77.2%	85.7%
55.3	62.0%	31.0%	86.6%	35.1%	23.2%	49.7%	37.0%	20.2%	41.9%	71.4%	46.3%	86.5%	28.6%	16.1%	43.9%	88.7%	16.4%	96.4%	82.3%	77.2%	85.6%
55.4	61.9%	30.9%	86.6%	35.1%	23.2%	49.7%	37.0%	20.2%	41.9%	71.4%	46.2%	86.5%	28.6%	16.0%	43.8%	88.7%	16.1%	96.4%	82.2%	77.1%	85.6%
55.5	61.8%	30.8%	86.5%	35.1%	23.2%	49.7%	37.0%	20.1%	41.9%	71.3%	46.1%	86.4%	28.5%	15.9%	43.7%	88.7%	15.6%	96.4%	82.2%	77.0%	85.6%
55.6	61.7%	30.7%	86.5%	35.1%	23.2%	49.6%	37.0%	20.0%	41.9%	71.3%	46.1%	86.4%	28.4%	15.9%	43.6%	88.7%	15.2%	96.4%	82.1%	76.9%	85.6%
55.7	61.6%	30.5%	86.4%	35.1%	23.2%	49.6%	37.0%	19.9%	41.9%	71.2%	46.0%	86.4%	28.3%	15.8%	43.5%	88.7%	14.8%	96.4%	82.0%	76.9%	85.5%
55.8	61.5%	30.4%	86.4%	35.1%	23.2%	49.6%	37.0%	19.8%	41.9%	71.2%	45.9%	86.3%	28.3%	15.8%	43.4%	88.7%	14.4%	96.4%	81.9%	76.9%	85.5%
55.9	61.4%	30.3%	86.3%	35.1%	23.2%	49.6%	37.0%	19.8%	41.9%	71.2%	45.8%	86.3%	28.2%	15.7%	43.4%	88.7%	14.0%	96.4%	81.9%	76.8%	85.4%
56	61.3%	30.2%	86.3%	35.1%	23.2%	49.6%	37.0%	19.8%	41.9%	71.1%	45.8%	86.3%	28.1%	15.7%	43.3%	88.7%	13.6%	96.4%	81.8%	76.7%	85.4%
56.1	61.2%	30.1%	86.2%	35.0%	23.1%	49.6%	37.0%	19.8%	41.9%	71.1%	45.7%	86.3%	28.1%	15.6%	43.2%	88.7%	13.2%	96.4%	81.7%	76.6%	85.4%
56.2	61.1%	30.0%	86.2%	35.0%	23.1%	49.6%	37.0%	19.7%	41.9%	71.0%	45.6%	86.3%	28.0%	15.6%	43.1%	88.7%	12.9%	96.4%	81.6%	76.5%	85.4%
56.3	61.0%	29.9%	86.1%	35.0%	23.1%	49.5%	37.0%	19.7%	41.9%	71.0%	45.5%	86.3%	27.9%	15.5%	43.0%	88.7%	12.5%	96.4%	81.6%	76.4%	85.3%
56.4	60.9%	29.8%	86.1%	35.0%	23.1%	49.5%	37.0%	19.7%	41.9%	71.0%	45.5%	86.3%	27.9%	15.5%	43.0%	88.7%	12.2%	96.4%	81.5%	76.4%	85.3%
56.5	60.8%	29.7%	86.0%	35.0%	23.1%	49.5%	37.0%	19.7%	41.9%	70.9%	45.4%	86.2%	27.8%	15.4%	42.9%	88.7%	12.0%	96.4%	81.4%	76.3%	85.3%
56.6	60.7%	29.6%	86.0%	35.0%	23.1%	49.5%	37.0%	19.7%	41.9%	70.9%	45.3%	86.2%	27.7%	15.4%	42.8%	88.7%	11.7%	96.4%	81.3%	76.2%	85.2%
56.7	60.6%	29.5%	85.9%	35.0%	23.1%	49.5%	37.0%	19.7%	41.9%	70.8%	45.3%	86.2%	27.7%	15.4%	42.8%	88.7%	11.4%	96.4%	81.2%	76.1%	85.2%
56.8	60.5%	29.4%	85.9%	35.0%	23.1%	49.5%	37.0%	19.7%	41.9%	70.8%	45.2%	86.2%	27.6%	15.3%	42.7%	88.7%	11.1%	96.4%	81.2%	76.0%	85.2%
56.9	60.4%	29.3%	85.8%	35.0%	23.1%	49.5%	37.0%	19.7%	41.9%	70.8%	45.1%	86.2%	27.5%	15.3%	42.6%	88.7%	10.8%	96.4%	81.1%	76.0%	85.2%

57	60.3%	29.2%	85.8%	35.0%	23.1%	49.5%	37.0%	19.7%	41.9%	70.7%	45.0%	86.2%	27.4%	15.2%	42.5%	88.7%	10.5%	96.4%	81.0%	75.9%	85.1%
57.1	60.2%	29.1%	85.7%	35.0%	23.1%	49.5%	37.0%	19.7%	41.9%				27.4%	15.2%	42.4%	88.7%	10.2%	96.4%	80.9%	75.8%	85.1%
57.2	60.2%	29.0%	85.7%	35.0%	23.1%	49.4%	37.0%	19.7%	41.9%				27.3%	15.1%	42.3%	88.7%	10.0%	96.4%	80.9%	75.7%	85.0%
57.3	60.1%	28.9%	85.6%	35.0%	23.1%	49.4%	37.0%	19.7%	41.8%				27.2%	15.1%	42.2%	88.7%	9.7%	96.4%	80.8%	75.6%	85.0%
57.4	60.0%	28.8%	85.6%	34.9%	23.1%	49.4%	37.0%	19.7%	41.8%				27.2%	15.0%	42.1%	88.7%	9.5%	96.4%	80.7%	75.5%	85.0%
57.5	59.9%	28.7%	85.5%	34.9%	23.1%	49.4%	37.0%	19.7%	41.8%				27.1%	14.9%	42.0%	88.7%	9.2%	96.4%	80.6%	75.3%	84.9%
57.6	59.8%	28.6%	85.5%	34.9%	23.1%	49.4%	37.0%	19.7%	41.8%				27.0%	14.9%	41.9%	88.7%	8.9%	96.4%	80.5%	75.2%	84.9%
57.7	59.7%	28.5%	85.4%	34.9%	23.1%	49.4%	37.0%	19.7%	41.8%				27.0%	14.8%	41.8%	88.7%	8.7%	96.4%	80.5%	75.1%	84.8%
57.8	59.6%	28.4%	85.4%	34.9%	23.1%	49.4%	37.0%	19.7%	41.8%				26.9%	14.8%	41.7%	88.7%	8.4%	96.4%	80.4%	75.0%	84.8%
57.9	59.5%	28.3%	85.4%	34.9%	23.1%	49.4%	37.0%	19.7%	41.8%				26.8%	14.7%	41.6%	88.7%	8.2%	96.4%	80.3%	74.8%	84.8%
58	59.5%	28.3%	85.3%	34.9%	23.1%	49.4%	37.0%	19.7%	41.8%				26.8%	14.7%	41.5%	88.7%	8.0%	96.4%	80.2%	74.7%	84.8%
58.1	59.4%	28.2%	85.3%	34.9%	23.1%	49.4%															
58.2	59.3%	28.1%	85.2%	34.9%	23.1%	49.4%															
58.3	59.2%	28.1%	85.2%	34.9%	23.1%	49.3%															
58.4	59.1%	28.0%	85.1%	34.9%	23.1%	49.3%															
58.5	59.0%	27.9%	85.1%	34.9%	23.1%	49.3%															
58.6	59.0%	27.9%	85.0%	34.9%	23.1%	49.3%															
58.7	58.9%	27.8%	85.0%	34.9%	23.1%	49.3%															
58.8	58.8%	27.7%	85.0%	34.9%	23.1%	49.3%															
58.9	58.7%	27.6%	84.9%	34.9%	23.1%	49.3%															
59	58.7%	27.5%	84.9%	34.9%	23.1%	49.3%															
59.1	58.6%	27.5%	84.8%	34.9%	23.1%	49.3%															
59.2	58.5%	27.4%	84.8%	34.9%	23.1%	49.3%															
59.3	58.4%	27.3%	84.7%	34.9%	23.1%	49.3%															
59.4	58.4%	27.3%	84.7%	34.9%	23.1%	49.3%															
59.5	58.3%	27.2%	84.6%	34.9%	23.1%	49.3%															
59.6	58.2%	27.1%	84.6%	34.9%	23.1%	49.3%															
59.7	58.2%	27.0%	84.6%	34.9%	23.1%	49.3%															
59.8	58.1%	27.0%	84.5%	34.8%	23.1%	49.3%															
59.9	58.0%	26.9%	84.5%	34.8%	23.0%	49.3%															
60	58.0%	26.8%	84.4%	34.8%	23.0%	49.3%															
60.1	57.9%	26.7%	84.4%	34.8%	23.0%	49.2%															

60.2	57.8%	26.7%	84.4%	34.8%	23.0%	49.2%
60.3	57.8%	26.6%	84.3%	34.8%	23.0%	49.1%
60.4	57.7%	26.5%	84.3%	34.8%	22.9%	49.1%
60.5	57.6%	26.4%	84.2%	34.8%	22.9%	49.0%
60.6	57.6%	26.3%	84.2%	34.8%	22.9%	49.0%
60.7	57.5%	26.3%	84.2%	34.8%	22.8%	49.0%
60.8	57.4%	26.2%	84.1%	34.8%	22.8%	49.0%
60.9	57.4%	26.2%	84.1%	34.8%	22.7%	49.0%
61	57.3%	26.1%	84.0%	34.8%	22.7%	49.0%
61.1	57.3%	26.1%	84.0%	34.8%	22.7%	49.0%
61.2	57.2%	26.0%	84.0%	34.8%	22.6%	49.0%
61.3	57.2%	26.0%	83.9%	34.8%	22.6%	49.0%
61.4	57.1%	25.9%	83.9%	34.8%	22.6%	49.0%
61.5	57.1%	25.8%	83.8%	34.8%	22.6%	48.9%
61.6	57.0%	25.8%	83.8%	34.8%	22.6%	48.9%
61.7	56.9%	25.7%	83.8%	34.8%	22.6%	48.8%
61.8	56.9%	25.7%	83.7%	34.9%	22.6%	48.8%
61.9	56.8%	25.6%	83.7%	34.9%	22.6%	48.8%
62	56.8%	25.6%	83.6%	34.9%	22.6%	48.8%
62.1	56.7%	25.6%	83.6%	34.9%	22.6%	48.8%
62.2	56.7%	25.5%	83.6%	34.9%	22.6%	48.8%
62.3	56.7%	25.5%	83.5%	34.9%	22.6%	48.8%
62.4	56.6%	25.4%	83.5%	34.9%	22.6%	48.8%
62.5	56.6%	25.4%	83.5%	34.9%	22.6%	48.8%
62.6	56.5%	25.4%	83.4%	34.9%	22.6%	48.8%
62.7	56.5%	25.3%	83.4%	34.9%	22.6%	48.8%
62.8	56.4%	25.3%	83.4%	34.9%	22.6%	48.7%
62.9	56.4%	25.3%	83.3%	34.9%	22.6%	48.7%
63	56.4%	25.3%	83.3%	34.9%	22.6%	48.7%
63.1	56.3%	25.2%	83.3%	34.9%	22.6%	48.7%
63.2	56.3%	25.2%	83.2%	34.9%	22.6%	48.7%
63.3	56.2%	25.2%	83.2%	34.9%	22.6%	48.7%

63.4	56.2%	25.1%	83.2%	34.9%	22.6%	48.7%
63.5	56.2%	25.1%	83.1%	34.9%	22.6%	48.7%
63.6	56.1%	25.1%	83.1%	34.9%	22.6%	48.6%
63.7	56.1%	25.1%	83.1%	34.9%	22.6%	48.6%
63.8	56.1%	25.0%	83.0%	34.9%	22.6%	48.6%
63.9	56.0%	25.0%	83.0%	34.9%	22.6%	48.5%
64	56.0%	25.0%	83.0%	34.9%	22.6%	48.5%
64.1	56.0%	25.0%	83.0%	34.9%	22.6%	48.5%
64.2	55.9%	25.0%	83.0%	34.9%	22.6%	48.5%
64.3	55.9%	24.9%	82.9%	34.9%	22.6%	48.5%
64.4	55.9%	24.9%	82.9%	34.9%	22.6%	48.5%
64.5	55.9%	24.9%	82.9%	34.9%	22.6%	48.4%
64.6	55.8%	24.8%	82.9%	34.9%	22.6%	48.4%
64.7	55.8%	24.8%	82.9%	34.9%	22.6%	48.3%
64.8	55.8%	24.7%	82.9%	34.9%	22.5%	48.2%
64.9	55.8%	24.6%	82.8%	34.9%	22.5%	48.2%
65	55.7%	24.6%	82.8%	34.9%	22.5%	48.2%
65.1	55.7%	24.5%	82.8%	34.9%	22.5%	48.2%
65.2	55.7%	24.5%	82.8%	34.9%	22.5%	48.2%
65.3	55.7%	24.4%	82.8%	34.9%	22.5%	48.2%
65.4	55.7%	24.3%	82.8%	34.9%	22.5%	48.2%
65.5	55.6%	24.3%	82.8%	34.8%	22.5%	48.2%
65.6	55.6%	24.2%	82.8%	34.8%	22.5%	48.2%
65.7	55.6%	24.2%	82.7%	34.8%	22.4%	48.2%
65.8	55.6%	24.2%	82.7%	34.8%	22.4%	48.2%
65.9	55.6%	24.2%	82.7%	34.8%	22.4%	48.2%
66	55.6%	24.2%	82.7%	34.8%	22.4%	48.2%
66.1	55.6%	24.2%	82.7%	34.8%	22.4%	48.2%
66.2	55.5%	24.2%	82.7%	34.8%	22.4%	48.2%
66.3	55.5%	24.2%	82.7%	34.8%	22.4%	48.1%
66.4	55.5%	24.2%	82.7%	34.7%	22.3%	48.1%
66.5	55.5%	24.1%	82.7%	34.7%	22.3%	48.1%

66.6	55.5%	24.0%	82.7%	34.7%	22.3%	48.1%
66.7	55.5%	24.0%	82.7%	34.7%	22.3%	48.0%
66.8	55.5%	23.9%	82.7%	34.7%	22.3%	48.0%
66.9	55.5%	23.8%	82.7%	34.7%	22.2%	48.0%
67	55.5%	23.8%	82.7%	34.7%	22.2%	47.9%
67.1	55.5%	23.7%	82.7%	34.6%	22.1%	47.9%
67.2	55.5%	23.7%	82.7%	34.6%	22.1%	47.8%
67.3	55.5%	23.7%	82.7%	34.6%	22.1%	47.8%
67.4	55.5%	23.7%	82.7%	34.6%	22.1%	47.8%
67.5	55.5%	23.7%	82.7%	34.6%	22.1%	47.7%
67.6	55.5%	23.6%	82.7%	34.5%	22.1%	47.7%
67.7	55.5%	23.6%	82.7%	34.5%	22.1%	47.6%
67.8	55.5%	23.6%	82.7%	34.5%	22.0%	47.6%
67.9	55.5%	23.6%	82.7%	34.5%	22.0%	47.5%
68	55.5%	23.5%	82.7%	34.5%	22.0%	47.5%
68.1	55.5%	23.5%	82.7%	34.4%	22.0%	47.5%
68.2	55.5%	23.5%	82.6%	34.4%	22.0%	47.4%
68.3	55.5%	23.5%	82.6%	34.4%	22.0%	47.4%
68.4	55.5%	23.5%	82.6%	34.4%	22.0%	47.4%
68.5	55.5%	23.4%	82.6%	34.3%	22.0%	47.4%
68.6	55.5%	23.4%	82.6%	34.3%	22.0%	47.4%
68.7	55.5%	23.4%	82.6%	34.3%	22.0%	47.4%
68.8	55.5%	23.4%	82.6%	34.3%	22.0%	47.4%
68.9	55.5%	23.4%	82.5%	34.2%	22.0%	47.4%
69	55.5%	23.3%	82.5%	34.2%	22.0%	47.4%
69.1	55.5%	23.3%	82.5%	34.2%	22.0%	47.4%
69.2	55.5%	23.3%	82.5%	34.1%	22.0%	47.4%
69.3	55.5%	23.3%	82.5%	34.1%	21.9%	47.4%
69.4	55.5%	23.3%	82.5%	34.1%	21.9%	47.4%
69.5	55.5%	23.2%	82.5%	34.1%	21.8%	47.4%
69.6	55.5%	23.2%	82.4%	34.0%	21.7%	47.4%
69.7	55.5%	23.2%	82.4%	34.0%	21.7%	47.3%

69.8	55.5%	23.2%	82.4%	34.0%	21.6%	47.3%
69.9	55.5%	23.2%	82.4%	33.9%	21.6%	47.2%
70	55.5%	23.1%	82.4%	33.9%	21.5%	47.1%
70.1	55.5%	23.1%	82.4%	33.9%	21.4%	47.0%
70.2	55.5%	23.1%	82.4%	33.8%	21.4%	47.0%
70.3	55.5%	23.1%	82.3%	33.8%	21.3%	47.0%
70.4	55.5%	23.1%	82.3%	33.7%	21.3%	47.0%
70.5	55.5%	23.0%	82.3%	33.7%	21.2%	46.9%
70.6	55.5%	23.0%	82.3%	33.7%	21.1%	46.9%
70.7	55.5%	23.0%	82.3%	33.6%	21.1%	46.8%
70.8	55.5%	22.9%	82.3%	33.6%	21.0%	46.8%
70.9	55.5%	22.8%	82.3%	33.6%	21.0%	46.7%
71	55.5%	22.8%	82.2%	33.5%	20.9%	46.7%
71.1	55.5%	22.7%	82.2%	33.5%	20.9%	46.6%
71.2	55.5%	22.6%	82.2%	33.4%	20.9%	46.6%
71.3	55.5%	22.6%	82.2%	33.4%	20.8%	46.6%
71.4	55.5%	22.5%	82.2%	33.4%	20.8%	46.6%
71.5	55.5%	22.4%	82.2%	33.3%	20.8%	46.6%
71.6	55.5%	22.4%	82.2%	33.3%	20.7%	46.5%
71.7	55.5%	22.4%	82.1%	33.2%	20.7%	46.4%
71.8	55.5%	22.4%	82.1%	33.2%	20.6%	46.3%
71.9	55.5%	22.4%	82.1%	33.2%	20.6%	46.2%
72	55.5%	22.4%	82.1%	33.1%	20.6%	46.1%
72.1	55.5%	22.4%	82.1%	33.1%	20.5%	46.1%
72.2	55.5%	22.4%	82.1%	33.0%	20.5%	46.1%
72.3	55.5%	22.4%	82.1%	33.0%	20.4%	46.1%
72.4	55.5%	22.3%	82.0%	32.9%	20.4%	46.1%
72.5	55.5%	22.3%	82.0%	32.9%	20.3%	46.0%
72.6	55.5%	22.3%	82.0%	32.8%	20.3%	46.0%
72.7	55.5%	22.2%	82.0%	32.8%	20.2%	46.0%
72.8	55.5%	22.2%	82.0%	32.7%	20.2%	46.0%
72.9	55.5%	22.2%	82.0%	32.7%	20.1%	46.0%

73	55.5%	22.2%	82.0%	32.6%	20.1%	46.0%
73.1	55.5%	22.2%	82.0%	32.6%	20.0%	46.0%
73.2	55.5%	22.1%	82.0%	32.5%	20.0%	46.0%
73.3	55.5%	22.1%	81.9%	32.5%	19.9%	46.0%
73.4	55.5%	22.0%	81.9%	32.4%	19.9%	46.0%
73.5	55.5%	21.9%	81.9%	32.4%	19.8%	46.0%
73.6	55.5%	21.9%	81.9%	32.3%	19.8%	45.9%
73.7	55.5%	21.8%	81.9%	32.3%	19.7%	45.9%
73.8	55.5%	21.8%	81.8%	32.2%	19.7%	45.8%
73.9	55.5%	21.8%	81.8%	32.2%	19.6%	45.8%
74	55.5%	21.7%	81.8%	32.1%	19.6%	45.7%
74.1	55.5%	21.7%	81.8%	32.1%	19.5%	45.6%
74.2	55.5%	21.7%	81.8%	32.0%	19.5%	45.5%
74.3	55.5%	21.6%	81.8%	32.0%	19.4%	45.4%
74.4	55.5%	21.6%	81.8%	31.9%	19.4%	45.3%
74.5	55.5%	21.5%	81.8%	31.8%	19.3%	45.3%
74.6	55.5%	21.5%	81.8%	31.8%	19.2%	45.2%
74.7	55.5%	21.5%	81.8%	31.7%	19.1%	45.0%
74.8	55.5%	21.5%	81.8%	31.7%	19.0%	44.9%
74.9	55.5%	21.5%	81.8%	31.6%	19.0%	44.9%
75	55.5%	21.4%	81.8%	31.6%	18.9%	44.9%
75.1	55.5%	21.4%	81.8%	31.5%	18.9%	44.9%
75.2	55.5%	21.3%	81.8%	31.4%	18.8%	44.8%
75.3	55.5%	21.3%	81.8%	31.4%	18.7%	44.8%
75.4	55.5%	21.2%	81.8%	31.3%	18.6%	44.7%
75.5	55.5%	21.2%	81.8%	31.3%	18.5%	44.7%
75.6	55.5%	21.1%	81.8%	31.2%	18.5%	44.7%
75.7	55.5%	21.1%	81.8%	31.2%	18.4%	44.7%
75.8	55.5%	21.0%	81.8%	31.1%	18.3%	44.7%
75.9	55.5%	21.0%	81.8%	31.0%	18.2%	44.7%
76	55.5%	20.9%	81.8%	31.0%	18.1%	44.7%
76.1	55.5%	20.9%	81.8%	30.9%	17.9%	44.7%

76.2	55.5%	20.8%	81.8%	30.9%	17.8%	44.7%
76.3	55.5%	20.8%	81.8%	30.8%	17.7%	44.7%
76.4	55.5%	20.7%	81.8%	30.8%	17.6%	44.7%
76.5	55.5%	20.7%	81.8%	30.7%	17.4%	44.7%
76.6	55.5%	20.6%	81.8%	30.6%	17.3%	44.7%
76.7	55.5%	20.5%	81.8%	30.6%	17.2%	44.7%
76.8	55.5%	20.4%	81.8%	30.5%	17.2%	44.7%
76.9	55.5%	20.3%	81.8%	30.5%	17.1%	44.7%
77	55.5%	20.2%	81.8%	30.4%	17.0%	44.7%
77.1	55.5%	20.1%	81.8%	30.4%	16.9%	44.7%
77.2	55.5%	20.1%	81.8%	30.3%	16.7%	44.7%
77.3	55.5%	20.0%	81.8%	30.3%	16.7%	44.7%
77.4	55.5%	19.9%	81.8%	30.2%	16.6%	44.7%
77.5	55.5%	19.8%	81.8%	30.1%	16.6%	44.7%
77.6	55.5%	19.7%	81.8%	30.1%	16.5%	44.7%
77.7	55.5%	19.6%	81.8%	30.0%	16.4%	44.7%
77.8	55.5%	19.5%	81.8%	30.0%	16.4%	44.7%
77.9	55.5%	19.5%	81.8%	29.9%	16.4%	44.7%
78	55.5%	19.4%	81.8%	29.9%	16.3%	44.7%
78.1	55.5%	19.3%	81.8%	29.8%	16.2%	44.7%
78.2	55.5%	19.3%	81.8%	29.8%	16.2%	44.7%
78.3	55.5%	19.2%	81.8%	29.7%	16.1%	44.6%
78.4	55.5%	19.2%	81.8%	29.6%	16.0%	44.6%
78.5	55.5%	19.1%	81.8%	29.6%	16.0%	44.5%
78.6	55.5%	19.1%	81.8%	29.5%	15.9%	44.5%
78.7	55.5%	19.0%	81.8%	29.5%	15.8%	44.4%
78.8	55.5%	19.0%	81.8%	29.4%	15.7%	44.4%
78.9	55.5%	18.9%	81.8%	29.4%	15.6%	44.3%
79	55.5%	18.9%	81.8%	29.3%	15.5%	44.2%
79.1	55.5%	18.9%	81.8%	29.3%	15.4%	44.1%
79.2	55.5%	18.9%	81.8%	29.2%	15.4%	44.0%
79.3	55.5%	18.9%	81.8%	29.1%	15.4%	44.0%

79.4	55.5%	18.9%	81.8%	29.1%	15.3%	44.0%
79.5	55.5%	18.9%	81.8%	29.0%	15.3%	44.0%
79.6	55.5%	18.9%	81.7%	29.0%	15.2%	43.9%
79.7	55.5%	18.9%	81.7%	28.9%	15.1%	43.9%
79.8	55.5%	18.9%	81.7%	28.9%	15.1%	43.8%
79.9	55.5%	18.8%	81.7%	28.8%	15.0%	43.8%
80	55.5%	18.8%	81.7%	28.8%	14.9%	43.8%
80.1				28.7%	14.9%	43.8%
80.2				28.7%	14.8%	43.8%
80.3				28.6%	14.7%	43.8%
80.4				28.6%	14.6%	43.8%
80.5				28.5%	14.6%	43.8%
80.6				28.4%	14.5%	43.8%
80.7				28.4%	14.5%	43.8%
80.8				28.3%	14.4%	43.8%
80.9				28.3%	14.3%	43.8%
81				28.2%	14.2%	43.8%
81.1				28.2%	14.2%	43.8%
81.2				28.1%	14.1%	43.8%
81.3				28.1%	14.0%	43.8%
81.4				28.0%	13.8%	43.8%
81.5				28.0%	13.7%	43.7%
81.6				27.9%	13.6%	43.7%
81.7				27.9%	13.6%	43.7%
81.8				27.8%	13.5%	43.7%
81.9				27.7%	13.4%	43.7%
82				27.7%	13.3%	43.7%
82.1				27.6%	13.2%	43.7%
82.2				27.6%	13.1%	43.7%
82.3				27.5%	13.0%	43.7%
82.4				27.5%	13.0%	43.7%
82.5				27.4%	12.9%	43.7%

82.6	27.4%	12.8%	43.7%
82.7	27.3%	12.8%	43.7%
82.8	27.3%	12.7%	43.6%
82.9	27.2%	12.7%	43.6%
83	27.2%	12.6%	43.5%
83.1	27.1%	12.6%	43.4%
83.2	27.1%	12.5%	43.3%
83.3	27.0%	12.4%	43.2%
83.4	27.0%	12.4%	43.2%
83.5	26.9%	12.3%	43.2%
83.6	26.9%	12.2%	43.2%
83.7	26.8%	12.1%	43.2%
83.8	26.8%	12.0%	43.2%
83.9	26.7%	12.0%	43.2%
84	26.7%	12.0%	43.2%
84.1	26.6%	11.9%	43.2%
84.2	26.5%	11.8%	43.2%
84.3	26.5%	11.7%	43.2%
84.4	26.4%	11.7%	43.2%
84.5	26.4%	11.6%	43.2%
84.6	26.3%	11.5%	43.2%
84.7	26.3%	11.4%	43.2%
84.8	26.2%	11.3%	43.2%
84.9	26.2%	11.3%	43.2%
85	26.1%	11.2%	43.2%
85.1	26.1%	11.1%	43.2%
85.2	26.0%	11.1%	43.2%
85.3	26.0%	11.0%	43.2%
85.4	25.9%	10.9%	43.2%
85.5	25.9%	10.8%	43.2%
85.6	25.8%	10.8%	43.2%
85.7	25.8%	10.7%	43.2%

85.8	25.7%	10.6%	43.2%
85.9	25.7%	10.6%	43.2%
86	25.6%	10.5%	43.2%

Table S3 Risk of Bias Assessment for Case-Control Studies

First author's name	1	2	3	4	5	6	7	8	9	10	11	12	Rating
Altarawneh H[5]	Yes	Yes	n/a	Yes	Yes	Yes	No	NR	Yes	No	n/a	Yes	Good
Rane M[6]	Yes	Yes	n/a	Yes	Yes	Yes	NR	NR	Yes	No	n/a	Yes	Good
Wang X[24]	Yes	Yes	n/a	NR	No	No	NR	NR	Yes	No	n/a	Yes	Poor
Altarawneh H[26]	Yes	Yes	n/a	Yes	Yes	Yes	No	NR	Yes	No	n/a	No	Fair
Cerqueira-Silva T[28]	Yes	Yes	n/a	Yes	Yes	Yes	No	NR	Yes	No	n/a	Yes	Good
Carazo S[67]	Yes	Yes	n/a	Yes	Yes	Yes	Yes	NR	Yes	No	n/a	Yes	Good
Vitale J[14]	No	Yes	n/a	Yes	Yes	Yes	CD	NR	Yes	No	n/a	Yes	Fair
Willett B[25]	Yes	Yes	n/a	Yes	Yes	No	NR	NR	Yes	No	n/a	Yes	poor
Chin E[40]	Yes	Yes	n/a	Yes	Yes	Yes	No	NR	Yes	No	n/a	Yes	Good
Ayoub H[42]	Yes	Yes	n/a	Yes	Yes	Yes	No	NR	Yes	No	n/a	Yes	Good
Carazo S[29]	Yes	Yes	n/a	Yes	Yes	Yes	Yes	NR	Yes	Yes	NR	Yes	Good
Altarawneh H[68]	Yes	Yes	n/a	Yes	Yes	Yes	NR	NR	Yes	Yes	No	No	Fair
Powell A[57]	Yes	Yes	n/a	Yes	Yes	Yes	NR	NR	Yes	Yes	no	Yes	Fair
Corrao G[60]	Yes	Yes	No	Yes	Yes	Yes	yes	NR	Yes	Yes	no	Yes	Fair

n/a = not available; NR = not reported; CD, cannot determine.

Quality Assessment for Case-Control Studies (https://www.nhlbi.nih.gov/health-topics/study-quality-assessment-tools):

1. Was the research question or objective in this paper clearly stated and appropriate?
2. Was the study population clearly specified and defined?
3. Did the authors include a sample size justification?
4. Were controls selected or recruited from the same or similar population that gave rise to the cases (including the same timeframe)?
5. Were the definitions, inclusion and exclusion criteria, algorithms or processes used to identify or select cases and controls valid, reliable, and implemented consistently across all study participants?
6. Were the cases clearly defined and differentiated from controls?
7. If less than 100 percent of eligible cases and/or controls were selected for the study, were the cases and/or controls randomly selected from those eligible?
8. Was there use of concurrent controls?
9. Were the investigators able to confirm that the exposure/risk occurred prior to the development of the condition or event that defined a participant as a case?
10. Were the measures of exposure/risk clearly defined, valid, reliable, and implemented consistently (including the same time period) across all study participants?

11. Were the assessors of exposure/risk blinded to the case or control status of participants?
12. Were key potential confounding variables measured and adjusted statistically in the analyses? If matching was used, did the investigators account for matching during study analysis?

Table S4 **Risk of Bias Assessment for Cohort Studies**

First author's name	1	2	3	4	5	6	7	8	9	10	11	12	13	14	Rating
Smid M[7]	Yes	Yes	NR	Yes	n/a	No	Yes	n/a	n/a	n/a	Yes	n/a	NR	Yes	Poor
Hall V[10]	Yes	Yes	Yes	Yes	Yes	Yes	Yes	No	No	Yes	Yes	NR	Yes	Yes	Good
Lumley S[18]	Yes	Yes	Yes	Yes	No	No	Yes	n/a	n/a	n/a	Yes	n/a	Yes	Yes	Fair
Hansen C[4]	Yes	Yes	Yes	Yes	n/a	No	Yes	n/a	n/a	n/a	Yes	n/a	Yes	Yes	Fair
Pilz S[11]	Yes	Yes	Yes	Yes	n/a	No	Yes	n/a	n/a	n/a	Yes	n/a	Yes	No	Fair
Sheehan M[13]	Yes	Yes	Yes	Yes	n/a	No	Yes	n/a	n/a	n/a	Yes	n/a	Yes	No	Fair
Wilkins J[15]	Yes	Yes	Yes	Yes	No	No	Yes	n/a	n/a	n/a	Yes	n/a	No	Yes	Fair
Letizia A[16]	Yes	Yes	Yes	Yes	n/a	Yes	Yes	No	No	No	Yes	n/a	Yes	Yes	Good
Abo-Leyah H[17]	Yes	Yes	Yes	Yes	n/a	No	Yes	n/a	n/a	n/a	Yes	n/a	NR	No	Fair
Leidi A[21]	Yes	Yes	Yes	Yes	No	No	Yes	n/a	n/a	n/a	Yes	n/a	Yes	No	Poor
Starrfelt J[27]	Yes	Yes	Yes	Yes	n/a	No	Yes	n/a	n/a	n/a	Yes	n/a	NR	Yes	Fair
Nordstrom P[30]	Yes	Yes	Yes	Yes	n/a	No	Yes	n/a	n/a	n/a	Yes	n/a	Yes	Yes	Fair
Gallais F[34]	Yes	Yes	Yes	Yes	No	Yes	Yes	No	No	No	Yes	No	Yes	No	Fair
Krutikov M[35]	Yes	Yes	Yes	Yes	No	No	Yes	n/a	n/a	n/a	Yes	n/a	NR	Yes	Fair
Harvey R[36]	Yes	Yes	Yes	Yes	No	No	Yes	n/a	n/a	n/a	Yes	n/a	No	No	Poor
Maier H[37]	Yes	Yes	Yes	Yes	No	Yes	Yes	No	No	No	Yes	NR	Yes	No	Fair
Chemaitelly H[38]	Yes	Yes	Yes	Yes	n/a	No	Yes	n/a	n/a	n/a	Yes	n/a	Yes	Yes	Fair
Satwik R[39]	No	Yes	Yes	Yes	No	No	Yes	n/a	n/a	n/a	Yes	n/a	Yes	Yes	Fair
Hall V[9]	Yes	Yes	Yes	Yes	n/a	Yes	Yes	No	No	Yes	Yes	NR	Yes	Yes	Good
Rennert L[12]	Yes	Yes	Yes	Yes	No	No	Yes	n/a	n/a	n/a	Yes	n/a	Yes	Yes	Fair
Berec L[69]	Yes	Yes	Yes	Yes	n/a	No	Yes	n/a	n/a	n/a	Yes	n/a	NR	Yes	Fair
Pouwels K[22]	Yes	Yes	Yes	Yes	n/a	Yes	Yes	Yes	No	Yes	Yes	NR	Yes	Yes	Good
Pritchard E[23]	Yes	Yes	Yes	Yes	n/a	Yes	Yes	Yes	No	Yes	Yes	NR	Yes	Yes	Good
Abu-Raddad L[8]	Yes	Yes	Yes	Yes	n/a	Yes	Yes	No	No	NR	Yes	NR	Yes	Yes	Fair
Breathnach A[19]	Yes	Yes	Yes	Yes	No	No	Yes	n/a	n/a	n/a	Yes	n/a	Yes	No	Fair
Manica M[70]	No	Yes	Yes	Yes	No	No	Yes	n/a	n/a	n/a	Yes	n/a	NR	No	Poor
Jeffery-Smith A[33]	No	Yes	Yes	Yes	No	No	Yes	n/a	n/a	n/a	Yes	n/a	Yes	No	Poor

Chemaitelly H[43]	Yes	Yes	No	Yes	n/a	No	Yes	n/a	n/a	n/a	Yes	n/a	Yes	Yes	Fair
Braeye T[41]	Yes	Yes	Yes	Yes	n/a	No	Yes	n/a	n/a	n/a	Yes	n/a	Yes	Yes	Fair
Office for National Statistics[31]	Yes	Yes	Yes	Yes	Yes	No	Yes	No	No	Yes	Yes	NR	NR	Yes	Good
Petras M[44]	Yes	Yes	Yes	Yes	No	Yes	Yes	No	Yes	No	Yes	No	Yes	Yes	Good
Abu-Raddad LJ[46]	Yes	Yes	yes	Yes	n/a	Yes	Yes	No	Yes	No	Yes	NR	Yes	No	Fair
Bertollini R[47]	No	Yes	No	Yes	No	Yes	Yes	No	Yes	No	Yes	No	Yes	No	Poor
Chemaitelly H[48]	Yes	Yes	Yes	Yes	n/a	Yes	Yes	No	Yes	No	Yes	NR	Yes	Yes	Good
Vicentini M[49]	Yes	Yes	Yes	Yes	n/a	Yes	Yes	No	Yes	No	Yes	NR	Yes	Yes	Good
Abu-Raddad LJ[50]	No	Yes	No	Yes	No	Yes	Yes	No	Yes	No	Yes	No	Yes	Yes	Fair
Michlmayr D[51]	Yes	Yes	Yes	Yes	n/a	Yes	Yes	No	Yes	No	Yes	NR	Yes	Yes	Good
Lacy J[52]	Yes	Yes	Yes	Yes	No	Yes	Yes	No	Yes	No	Yes	No	NR	No	Fair
Ridgway JP[53]	Yes	CD	NR	Yes	n/a	Yes	Yes	No	No	No	Yes	NR	NR	Yes	Poor
Spicer KB[54]	Yes	Yes	Yes	Yes	n/a	Yes	Yes	No	Yes	No	yes	NR	Yes	No	Fair
Cohen C[55]	Yes	Yes	Yes	Yes	No	Yes	Yes	No	Yes	No	yes	No	Yes	Yes	Good
Sun K[56]	Yes	Yes	Yes	Yes	No	Yes	Yes	No	Yes	No	yes	No	Yes	Yes	Good
Andeweg S[66]	Yes	Yes	NR	Yes	NA	Yes	Yes	No	Yes	No	yes	NR	NR	No	Poor
Murugesan M[58]	Yes	Yes	Yes	Yes	No	Yes	Yes	No	Yes	No	yes	No	Yes	No	Fair
Dhumal S[59]	Yes	Yes	Yes	Yes	NA	Yes	Yes	No	Yes	No	yes	No	Yes	Yes	Good

Narrainen F[63]	Yes	Yes	Yes	Yes	No	Yes	Yes	No	Yes	No	yes	No	Yes	No	Fair
Reynolds S[62]	Yes	Yes	Yes	Yes	NA	Yes	Yes	No	Yes	No	yes	Yes	Yes	Yes	Good
Kubale J[64]	Yes	Yes	Yes	Yes	NA	Yes	Yes	No	Yes	No	yes	No	Yes	No	Fair
Rothberg M[65]	Yes	Yes	Yes	Yes	NA	Yes	Yes	No	Yes	No	yes	No	Yes	Yes	Good
Smolenov I[61]	Yes	Yes	Yes	Yes	No	Yes	Yes	No	Yes	No	yes	Yes	Yes	No	Fair

n/a = not available
NR = not reported

Quality Assessment Tool for Observational Cohort and Cross-Sectional Studies (https://www.nhlbi.nih.gov/health-topics/study-quality-assessment-tools):

1. Was the research question or objective in this paper clearly stated?
2. Was the study population clearly specified and defined?
3. Was the participation rate of eligible persons at least 50%?
4. Were all the subjects selected or recruited from the same or similar populations (including the same time period)? Were inclusion and exclusion criteria for being in the study prespecified and applied uniformly to all participants?
5. Was a sample size justification, power description, or variance and effect estimates provided?
6. For the analyses in this paper, were the exposure(s) of interest measured prior to the outcome(s) being measured?
7. Was the timeframe sufficient so that one could reasonably expect to see an association between exposure and outcome if it existed?
8. For exposures that can vary in amount or level, did the study examine different levels of the exposure as related to the outcome (e.g., categories of exposure, or exposure measured as continuous variable)?
9. Were the exposure measures (independent variables) clearly defined, valid, reliable, and implemented consistently across all study participants?
10. Was the exposure(s) assessed more than once over time?
11. Were the outcome measures (dependent variables) clearly defined, valid, reliable, and implemented consistently across all study participants?
12. Were the outcome assessors blinded to the exposure status of participants?
13. Was loss to follow-up after baseline 20% or less?
14. Were key potential confounding variables measured and adjusted statistically for their impact on the relationship between exposure(s) and outcome(s)?

Section 5 Reference list

1. Stevens, G. A. *et al.* Guidelines for Accurate and Transparent Health Estimates Reporting: the GATHER statement. *The Lancet* **388**, e19–e23 (2016).
2. Page, M. J. *et al.* The PRISMA 2020 statement: an updated guideline for reporting systematic reviews. *BMJ* n71 (2021) doi:10.1136/bmj.n71.
3. Berec, L. *et al.* Protection provided by vaccination, booster doses and previous infection against covid-19 infection, hospitalisation or death over time in Czechia. *PLOS ONE* **17**, e0270801 (2022).
4. Hansen, C. H., Michlmayr, D., Gubbels, S. M., Mølbak, K. & Ethelberg, S. Assessment of protection against reinfection with SARS-CoV-2 among 4 million PCR-tested individuals in Denmark in 2020: a population-level observational study. *Lancet Lond. Engl.* **397**, 1204–1212 (2021).
5. Altarawneh, H. N. *et al.* Protection against the Omicron Variant from Previous SARS-CoV-2 Infection. *N. Engl. J. Med.* **386**, 1288–1290 (2022).
6. Rane, M. S. *et al. Effectiveness of Covid-19 vaccines against symptomatic and asymptomatic SARS-CoV-2 infections in an urgent care setting*. http://medrxiv.org/lookup/doi/10.1101/2022.02.21.22271298 (2022) doi:10.1101/2022.02.21.22271298.
7. Šmíd, M. *et al.* Protection by vaccines and previous infection against the Omicron variant of SARS-CoV-2. *J. Infect. Dis.* jiac161 (2022) doi:10.1093/infdis/jiac161.
8. Abu-Raddad, L. J. *et al.* SARS-CoV-2 antibody-positivity protects against reinfection for at least seven months with 95% efficacy. *EClinicalMedicine* **35**, 100861 (2021).
9. Hall, V. J. *et al.* SARS-CoV-2 infection rates of antibody-positive compared with antibody-negative health-care workers in England: a large, multicentre, prospective cohort study (SIREN). *Lancet Lond. Engl.* **397**, 1459–1469 (2021).
10. Hall, V. *et al.* Protection against SARS-CoV-2 after Covid-19 Vaccination and Previous Infection. *N. Engl. J. Med.* **386**, 1207–1220 (2022).
11. Pilz, S. *et al.* SARS-CoV-2 re-infection risk in Austria. *Eur. J. Clin. Invest.* **51**, (2021).
12. Rennert, L. & McMahan, C. Risk of Severe Acute Respiratory Syndrome Coronavirus 2 (SARS-CoV-2) Reinfection in a University Student Population. *Clin. Infect. Dis.* **74**, 719–722 (2022).
13. Sheehan, M. M., Reddy, A. J. & Rothberg, M. B. Reinfection Rates Among Patients Who Previously Tested Positive for Coronavirus Disease 2019: A Retrospective Cohort Study. *Clin. Infect. Dis.* **73**, 1882–1886 (2021).

14. Vitale, J. *et al.* Assessment of SARS-CoV-2 Reinfection 1 Year After Primary Infection in a Population in Lombardy, Italy. *JAMA Intern. Med.* **181**, 1407 (2021).

15. Wilkins, J. T. *et al.* Serologic Status and SARS-CoV-2 Infection over 6 Months of Follow Up in Healthcare Workers in Chicago: A Cohort Study. *Infect. Control Hosp. Epidemiol.* 1–9 (2021) doi:10.1017/ice.2021.367.

16. Letizia, A. G. *et al.* SARS-CoV-2 seropositivity and subsequent infection risk in healthy young adults: a prospective cohort study. *Lancet Respir. Med.* **9**, 712–720 (2021).

17. Abo-Leyah, H. *et al.* The protective effect of SARS-CoV-2 antibodies in Scottish healthcare workers. *ERJ Open Res.* **7**, 00080–02021 (2021).

18. Lumley, S. F. *et al.* An Observational Cohort Study on the Incidence of Severe Acute Respiratory Syndrome Coronavirus 2 (SARS-CoV-2) Infection and B.1.1.7 Variant Infection in Healthcare Workers by Antibody and Vaccination Status. *Clin. Infect. Dis.* **74**, 1208–1219 (2022).

19. Breathnach, A. S. *et al.* Prior COVID-19 significantly reduces the risk of subsequent infection, but reinfections are seen after eight months. *J. Infect.* **82**, e11–e12 (2021).

20. Manica, M. *et al.* Risk of Symptomatic Infection During a Second Coronavirus Disease 2019 Wave in Severe Acute Respiratory Syndrome Coronavirus 2–Seropositive Individuals. *Clin. Infect. Dis.* **74**, 893–896 (2022).

21. Leidi, A. *et al.* Risk of Reinfection After Seroconversion to Severe Acute Respiratory Syndrome Coronavirus 2 (SARS-CoV-2): A Population-based Propensity-score Matched Cohort Study. *Clin. Infect. Dis.* **74**, 622–629 (2022).

22. Pouwels, K. B. *et al.* Effect of Delta variant on viral burden and vaccine effectiveness against new SARS-CoV-2 infections in the UK. *Nat. Med.* **27**, 2127–2135 (2021).

23. Pritchard, E. *et al.* Impact of vaccination on new SARS-CoV-2 infections in the United Kingdom. *Nat. Med.* **27**, 1370–1378 (2021).

24. Wang, X., Zein, J., Ji, X. & Lin, D.-Y. *Impact of Vaccination, Prior Infection, and Therapy on Delta and Omicron Variants*. http://medrxiv.org/lookup/doi/10.1101/2022.03.24.22272901 (2022) doi:10.1101/2022.03.24.22272901.

25. Willett, B. J. *et al.* SARS-CoV-2 Omicron is an immune escape variant with an altered cell entry pathway. *Nat. Microbiol.* **7**, 1161–1179 (2022).

26. Altarawneh, H. N. *et al.* Effects of Previous Infection and Vaccination on Symptomatic Omicron Infections. *N. Engl. J. Med.* **387**, 21–34 (2022).

27. Starrfelt, J. *et al.* Age and product dependent vaccine effectiveness against SARS-CoV-2 infection and hospitalisation among adults in Norway: a national cohort study, July–November 2021. *BMC Med.* **20**, 278 (2022).

28. Cerqueira-Silva, T. *et al.* Vaccination plus previous infection: protection during the omicron wave in Brazil. *Lancet Infect. Dis.* S1473309922002882 (2022) doi:10.1016/S1473-3099(22)00288-2.

29. Carazo, S. *et al.* Protection against omicron (B.1.1.529) BA.2 reinfection conferred by primary omicron BA.1 or pre-omicron SARS-CoV-2 infection among health-care workers with and without mRNA vaccination: a test-negative case-control study. *Lancet Infect. Dis.* S1473-3099(22)00578–3 (2022) doi:10.1016/S1473-3099(22)00578-3.

30. Nordström, P., Ballin, M. & Nordström, A. Risk of SARS-CoV-2 reinfection and COVID-19 hospitalisation in individuals with natural and hybrid immunity: a retrospective, total population cohort study in Sweden. *Lancet Infect. Dis.* **22**, 781–790 (2022).

31. Coronavirus (COVID-19) Infection Survey, characteristics of people testing positive for COVID-19, UK - Office for National Statistics. https://www.ons.gov.uk/peoplepopulationandcommunity/healthandsocialcare/conditionsanddiseases/bulletins/coronaviruscovid19infectionsurveycharacteristicsofpeopletestingpositiveforcovid19uk/25august2021.

32. Omicron largely evades immunity from past infection or two vaccine doses | Imperial News | Imperial College London. https://www.imperial.ac.uk/news/232698/omicron-largely-evades-immunity-from-past/.

33. Jeffery-Smith, A. *et al.* Antibodies to SARS-CoV-2 protect against re-infection during outbreaks in care homes, September and October 2020. *Euro Surveill. Bull. Eur. Sur Mal. Transm. Eur. Commun. Dis. Bull.* **26**, (2021).

34. Gallais, F. *et al.* Evolution of antibody responses up to 13 months after SARS-CoV-2 infection and risk of reinfection. *EBioMedicine* **71**, 103561 (2021).

35. Krutikov, M. *et al.* Incidence of SARS-CoV-2 infection according to baseline antibody status in staff and residents of 100 long-term care facilities (VIVALDI): a prospective cohort study. *Lancet Healthy Longev.* **2**, e362–e370 (2021).

36. Harvey, R. A. *et al.* Association of SARS-CoV-2 Seropositive Antibody Test With Risk of Future Infection. *JAMA Intern. Med.* **181**, 672–679 (2021).
37. Maier, H. E. *et al.* Clinical spectrum of SARS-CoV-2 infection and protection from symptomatic re-infection. *Clin. Infect. Dis. Off. Publ. Infect. Dis. Soc. Am.* ciab717 (2021) doi:10.1093/cid/ciab717.
38. Chemaitelly, H. *et al.* Protection of Omicron sub-lineage infection against reinfection with another Omicron sub-lineage. *Nat. Commun.* **13**, 4675 (2022).
39. Satwik, R., Satwik, A., Katoch, S. & Saluja, S. ChAdOx1 nCoV-19 effectiveness during an unprecedented surge in SARS COV-2 infections. *Eur. J. Intern. Med.* **93**, 112–113 (2021).
40. Chin, E. T. *et al. Protection against Omicron conferred by mRNA primary vaccine series, boosters, and prior infection*. http://medrxiv.org/lookup/doi/10.1101/2022.05.26.22275639 (2022) doi:10.1101/2022.05.26.22275639.
41. Braeye, T. *et al.* Vaccine effectiveness against onward transmission of SARS-CoV2-infection by variant of concern and time since vaccination, Belgian contact tracing, 2021. *Vaccine* **40**, 3027–3037 (2022).
42. Ayoub, H. H. *et al. Estimating protection afforded by prior infection in preventing reinfection: Applying the test-negative study design*. http://medrxiv.org/lookup/doi/10.1101/2022.01.02.22268622 (2022) doi:10.1101/2022.01.02.22268622.
43. Chemaitelly, H., Bertollini, R. & Abu-Raddad, L. J. Efficacy of Natural Immunity against SARS-CoV-2 Reinfection with the Beta Variant. *N. Engl. J. Med.* **385**, 2585–2586 (2021).
44. Petráš, M. *et al.* The Effectiveness of Post-Vaccination and Post-Infection Protection in the Hospital Staff of Three Prague Hospitals: A Cohort Study of 8-Month Follow-Up from the Start of the COVID-19 Vaccination Campaign (COVANESS). *Vaccines* **10**, 9 (2021).
45. Altarawneh, H. N. *et al.* Protective Effect of Previous SARS-CoV-2 Infection against Omicron BA.4 and BA.5 Subvariants. *N. Engl. J. Med.* **387**, 1620–1622 (2022).
46. Abu-Raddad, L. J. *et al.* Introduction and expansion of the SARS-CoV-2 B.1.1.7 variant and reinfections in Qatar: A nationally representative cohort study. *PLOS Med.* **18**, e1003879 (2021).
47. Bertollini, R. *et al.* Associations of Vaccination and of Prior Infection With Positive PCR Test Results for SARS-CoV-2 in Airline Passengers Arriving in Qatar. *JAMA* **326**, 185 (2021).

48. Chemaitelly, H. *et al.* Duration of immune protection of SARS-CoV-2 natural infection against reinfection. *J. Travel Med.* taac109 (2022) doi:10.1093/jtm/taac109.

49. Vicentini, M. *et al.* Risk of SARS-CoV-2 Reinfection by Vaccination Status, Predominant Variant, and Time from Previous Infection: A Cohort Study in Italy. *SSRN Electron. J.* (2022) doi:10.2139/ssrn.4132329.

50. Abu-Raddad, L. J., Chemaitelly, H. & Bertollini, R. Severity of SARS-CoV-2 Reinfections as Compared with Primary Infections. *N. Engl. J. Med.* **385**, 2487–2489 (2021).

51. Michlmayr, D. *et al.* Observed protection against SARS-CoV-2 reinfection following a primary infection: A Danish cohort study among unvaccinated using two years of nationwide PCR-test data. *Lancet Reg. Health - Eur.* **20**, 100452 (2022).

52. Lacy, J. *et al.* Protective effect of a first SARS-CoV-2 infection from reinfection: a matched retrospective cohort study using PCR testing data in England. *Epidemiol. Infect.* **150**, e109 (2022).

53. Ridgway, J. P., Tideman, S., Wright, B. & Robicsek, A. Rates of COVID-19 Among Unvaccinated Adults With Prior COVID-19. *JAMA Netw. Open* **5**, e227650 (2022).

54. Spicer, K. B., Glick, C., Cavanaugh, A. M. & Thoroughman, D. Protective Immunity after Natural Infection with Severe Acute Respiratory Syndrome Coronavirus-2 (SARS-CoV-2) – Kentucky, USA, 2020. *Int. J. Infect. Dis.* **114**, 21–28 (2022).

55. Cohen, C. *et al.* SARS-CoV-2 incidence, transmission, and reinfection in a rural and an urban setting: results of the PHIRST-C cohort study, South Africa, 2020–21. *Lancet Infect. Dis.* **22**, 821–834 (2022).

56. Sun, K. *et al.* SARS-CoV-2 transmission, persistence of immunity, and estimates of Omicron's impact in South African population cohorts. *Sci. Transl. Med.* **14**, eabo7081 (2022).

57. Powell, A. A. et al. *Protection against symptomatic disease with the delta and omicron BA.1/BA.2 variants of SARS-CoV-2 after infection and vaccination in adolescents: national observational test-negative case control study, August 2021 to March 2022, England*. http://medrxiv.org/lookup/doi/10.1101/2022.08.19.22278987 (2022) doi:10.1101/2022.08.19.22278987.

58. Murugesan, M. *et al.* Protective effect conferred by prior infection and vaccination on COVID-19 in a healthcare worker cohort in South India. *PLOS ONE* **17**, e0268797 (2022).

59. Dhumal, S. *et al.* SARS-CoV-2 reinfection after previous infection and vaccine breakthrough infection through the second wave of pandemic in India: An observational study. *Int. J. Infect. Dis.* **118**, 95–103 (2022).

60. Corrao, G. *et al.* Protective action of natural and induced immunization against the occurrence of delta or alpha variants of SARS-CoV-2 infection: a test-negative case-control study. *BMC Med.* **20**, 52 (2022).
61. Smolenov, I. *et al.* Impact of previous exposure to SARS-CoV-2 and of S-Trimer (SCB-2019) COVID-19 vaccination on the risk of reinfection: a randomised, double-blinded, placebo-controlled, phase 2 and 3 trial. *Lancet Infect. Dis.* **22**, 990–1001 (2022).
62. Reynolds, S. L. *et al. Duration of Protection Against SARS-CoV-2 Reinfection and Associated Risk of Reinfection Assessed with Real-World Data*. http://medrxiv.org/lookup/doi/10.1101/2022.02.25.22271515 (2022) doi:10.1101/2022.02.25.22271515.
63. Narrainen, F. *et al.* The protective effect of previous COVID-19 infection in a high-prevalence hospital setting. *Clin. Med.* **21**, e470–e474 (2021).
64. Kubale, J. *et al. Burden of SARS-CoV-2 and protection from symptomatic second infection in children*. http://medrxiv.org/lookup/doi/10.1101/2022.01.03.22268684 (2022) doi:10.1101/2022.01.03.22268684.
65. Rothberg, M. B., Kim, P., Shrestha, N. K., Kojima, L. & Tereshchenko, L. G. Protection Against the Omicron Variant Offered by Previous Severe Acute Respiratory Syndrome Coronavirus 2 Infection: A Retrospective Cohort Study. *Clin. Infect. Dis.* ciac604 (2022) doi:10.1093/cid/ciac604.
66. Andeweg, S. P. *et al.* Protection of COVID-19 vaccination and previous infection against Omicron BA.1, BA.2 and Delta SARS-CoV-2 infections. *Nat. Commun.* **13**, 4738 (2022).
67. Carazo, S. *et al. Protection against Omicron re-infection conferred by prior heterologous SARS-CoV-2 infection, with and without mRNA vaccination*. http://medrxiv.org/lookup/doi/10.1101/2022.04.29.22274455 (2022) doi:10.1101/2022.04.29.22274455.
68. Altarawneh, H. N. *et al. Protection of SARS-CoV-2 natural infection against reinfection with the Omicron BA.4 or BA.5 subvariants*. http://medrxiv.org/lookup/doi/10.1101/2022.07.11.22277448 (2022) doi:10.1101/2022.07.11.22277448.
69. Berec, L. *et al. Real-life protection provided by vaccination, booster doses and previous infection against covid-19 infection, hospitalisation or death over time in the Czech Republic: a whole country retrospective view*. http://medrxiv.org/lookup/doi/10.1101/2021.12.10.21267590 (2021) doi:10.1101/2021.12.10.21267590.

70. Manica, M. *et al. The risk of symptomatic reinfection during the second COVID-19 wave in individuals previously exposed to SARS-CoV-2*. http://medrxiv.org/lookup/doi/10.1101/2021.04.14.21255502 (2021) doi:10.1101/2021.04.14.21255502.
